# Intramolecular Hydrogen Bonding and Conformational Preferences of Arzanol—An Antioxidant Acylphloroglucinol

**DOI:** 10.3390/molecules22081294

**Published:** 2017-08-03

**Authors:** Liliana Mammino

**Affiliations:** Department of Chemistry, University of Venda, Thohoyandou 0950, South Africa; Liliana.Mammino@univen.ac.za or sasdestria@yahoo.com; Tel.: +27-(0)15-962-8147

**Keywords:** acylphloroglucinols, antioxidants, arzanol, intramolecular hydrogen bonding, polyphenolic compounds, O–H···π interaction

## Abstract

Arzanol is a naturally-occurring prenylated acylphloroglucinol isolated from *Helichrysum italicum* and exhibiting anti-oxidant, antibiotic and antiviral activities. The molecule contains an α-pyrone moiety attached to the phloroglucinol moiety through a methylene bridge. The presence of several hydrogen bond donor or acceptor sites makes intramolecular hydrogen bonding patterns the dominant stabilising factor. Conformers with all the possible different hydrogen bonding patterns were calculated at the HF/6-31G(d,p) and DFT/B3LYP/6-31+G(d,p) levels with fully relaxed geometry in vacuo and in three solvents—chloroform, acetonitrile and water (these levels being chosen to enable comparisons with previous studies on acylphloroglucinols). Calculations in solution were performed with the Polarisable Continuum Model. The results show that the lowest energy conformers have the highest number of stronger intramolecular hydrogen bonds. The influence of intramolecular hydrogen bonding patterns on the other molecular properties is also analysed.

## 1. Introduction

**Figure 1:**
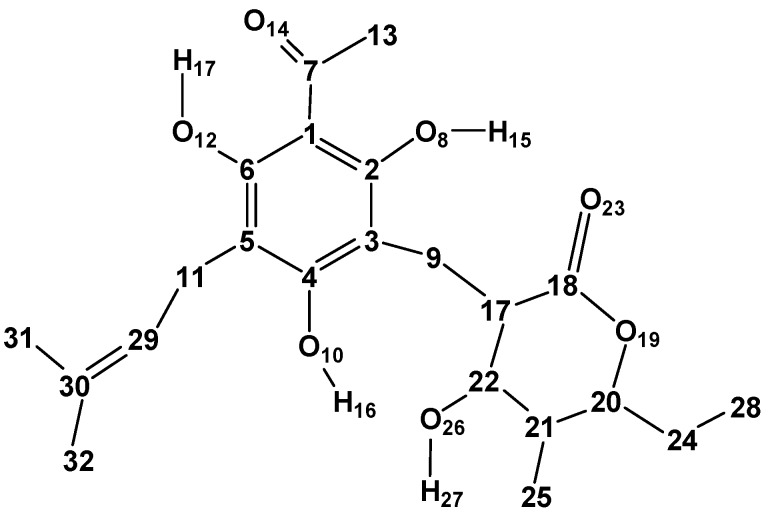
Structure of the arzanol molecule and atom numbering utilised in this work. The figure shows the carbon skeleton of the molecule, the O atoms, and the H atoms pertaining to OH groups. The other H atoms are hidden, to better highlight the molecular structure. The C atoms are denoted by their numbers.

Arzanol (C_22_H_26_O_7_, Figure 1) is a naturally occurring acylphloroglucinol (ACPL) and is the major responsible for the anti-inflammatory, anti-oxidant, antibiotic and antiviral activities of *Helichrysum italicum* [1,2,3]. Antioxidant compounds are biologically important because they protect the organism against reactive oxygen species (ROS), thus contributing to prevent damages to the central nervous system and the insurgence of neurodegenerative diseases such as ischemia, Alzheimer’s disease, Parkinson’s disease and schizophrenia [4,5].

ACPLs [6] are derivatives of phloroglucinol (1,3,5-trihydroxybenzene) characterised by the presence of a COR group (acyl group), whose sp^2^ O can form an intramolecular hydrogen bond (IHB) with one of the two ortho OHs; following a practice introduced in previous works on ACPLs [7,8,9,10,11], this IHB (which is present in nearly all ACPLs) is here termed ‘first IHB’. In arzanol, R is a methyl group; an α-pyrone ring is attached to one position *meta* to the COR group (C3) and a prenyl chain is attached to the other *meta* position (C5). For the sake of conciseness, arzanol will be hereafter denoted as ARZ, the acylphloroglucinol moiety as PHL, the α-pyrone moiety as PYR and the prenyl chain as PRN.

The computational study of antioxidant ACPLs responds to the known tight relationships between the molecular properties of polyphenolic compounds and their biological activities [12,13,14]. The study of the ARZ molecule is also interesting in view of new information on ACPLs, as the fact that the PHL moiety is bonded to a ring of a different nature (PYR) makes ARZ different from the ACPLs considered so far [7,8,9,10,11], including the dimeric ACPLs in which both units are acylphloroglucinol moieties [15].

Thanks to the presence of the sp^2^ O and of three phenol OH in the PHL moiety, and an H-bond donor and two acceptors in the PYR moiety (H27, O23 and O26), the ARZ molecule can form up to three O–H···O IHBs simultaneously: the first IHB and two IHBs between the two moieties (one on each side of the methylene bridge); the latter will be termed IMHBs (for ‘intermoiety hydrogen bonds’) when it is expedient to emphasise that they join the two moieties (whereas IHBs may be used comprehensively when all the IHBs are considered). Furthermore, H16 or H17, when suitably oriented, can interact with the π bond in the PRN chain (O–H···π interaction, which is a type of H-bond). Thus, IHB patterns are the major features characterising conformers. The current work focuses mainly on ARZ IHB patterns. Because of this, high energy conformers have been calculated to obtain indications on the stabilising effect of each IHB, or set of IHBs, by considering the effect of their removal, although such conformers are largely unpopulated and not interesting as potential responsible of biological activities (for which a cautious threshold would consider only those with relative energy ≤3.5 kcal/mol in vacuo or in suitable solvents).

**Figure 2:**
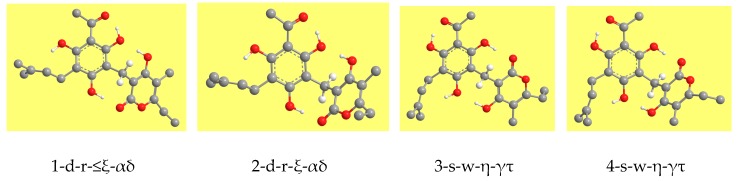
The four mutual orientations of the two rings in the arzanol molecule. The H atoms attached to C atoms are hidden to better highlight the geometry of the molecular skeleton. Only the H atoms attached to C9 are shown because they are functional to highlighting the mutual orientations of the two rings and the orientation of the methylene bridge.

The mutual orientations of the PHL and PYR rings determine four sets of conformers, here denoted by different numbers (Figure 2). The set denoted by the number 3 had been the object of a preliminary study [16] exploring the main features to be considered in a thorough conformational study of the molecule. Orientations 1 and 2 differ only by the fact that the PYR ring is symmetrical with respect to the plane of the benzene ring; the same is true for orientations 3 and 4. IMHB patterns are different for the two sets (those of orientations 1 and 2 are different from those of orientations 3 and 4).

Conformers were calculated in the gas phase and in solution, considering the same three solvents as in the previous studies of ACPLs (chloroform, acetonitrile and water, [7,8,9,10,11]) to enable informative comparisons. These solvents cover the ranges of polarities and H-bonding abilities of the media in which a biologically active molecule may preferably be present within a living organism. An approximate estimation [17] of the octanol/water partition coefficient of ARZ yields 4.47673, suggesting that it may mostly be present in non-polar media, which would be satisfactorily modelled by chloroform. However, water always needs to be considered because it is by far the dominant medium in living organisms (with a proportion of ~70% in the human body); furthermore, the presence of several H-bond donor or acceptor sites in the ARZ molecule increases interest in considering its behaviour in water solution. Acetonitrile is the most commonly utilised non-protic polar solvent, both in calculations and in experimental studies.

The results confirm the stabilising effects of IHBs. The four lowest energy conformers contain three O–H···O IHBs and also the O–H···π interaction involving the π bond of the PRN chain.

## 2. Computational Details

Calculations were performed with the same levels of theory as in previous studies of ACPLs [7,8,9,10,11], to enable informative comparisons: Hartree Fock (HF) with the 6-31G(d,p) basis set and Density Functional Theory (DFT) with the B3LYP functional and the 6-31+G(d,p) basis set, both with fully relaxed geometry, and MP2 single-point (SP) calculations on the HF-optimised conformers. The general studies of ACPLs [7,8,9,10,11] had shown that HF gives reasonable results for this class of compounds and enables realistic trend-identifications with the reasonably cheap 6-31G(d,p) basis set. Inputs were prepared by considering all the possible IHB patterns for each mutual orientation of the two moieties; for each pattern, features already identified as relevant or non-negligible, such as the orientations of the phenol OHs not engaged in IHBs [7,8,9,10,11], were also considered.

DFT calculations were performed with fully relaxed geometry, utilising the HF-optimised geometries as inputs. DFT takes into account part of the correlation energy (whereas HF takes into account only the part related to Pauli’s exclusion principle). The B3LYP functional [18,19,20] is the most widely utilised functional in molecular calculations [21]. The 6-31+G(d,p) basis set was selected for DFT calculations because previous studies [7,8,9,10,11] had shown that the performance of DFT without diffuse functions on the heavy atoms is rather poor for ACPLs.

Vibrational frequencies (harmonic approximation) were calculated to verify that the identified stationary points are true minima, to obtain the zero-point energy (ZPE) corrections and to obtain an idea of the strength of the various IHBs through the red shifts of the frequency of the donor OHs. They were calculated at the DFT/B3LYP/6-31+G(d,p) level and scaled by 0.964, a factor recommended for this level [22].

The Møller–Plesset perturbation theory (MP2) takes into account both electron correlation and dispersion effects, which both play significant roles in H-bonding. However, MP2 calculations with fully relaxed geometry are very expensive for a molecule of this size (55 atoms). SP MP2 calculations were performed on the HF optimised geometries (the choice of HF results as the ones on which to perform MP2 calculations is natural, because an HF calculation constitutes the first step—providing the unperturbed results—in the MP2 algorithm). Although they do not improve the IHB parameters because of lack of geometry flexibility, SP MP2 results are useful for an additional comparison of energy trends.

Calculations in solution were performed with the Polarisable Continuum Model (PCM, [23,24,25,26,27,28]), in which the solvent is modelled by a continuous isotropic dielectric and the solute molecule is viewed as embedded in a cavity surrounded by the continuum solvent, with the geometry of the cavity following the geometry of the solute molecule consistently with its accessible surface for the given solvent. The default settings of Gaussian03 [29] for PCM were utilised, with the only exception of the SCFVAC non-standard input option to obtain more thermodynamic data. The calculations were performed at the DFT/B3LYP/6-31+G(d,p) level, both with full reoptimisation starting from the in-vacuo-optimised geometries and as SP calculations. A comparison of the two sets of results enables an assessment of the performance of SP calculations—relevant in view of the occurrence of PCM optimisation failure in a number of calculations with fully relaxed geometry.

All the calculations were performed with Gaussian 03, revision D01 [29].

All the energy values reported are in kcal/mol and all the length values are in Å. For conciseness sake, acronyms are utilised for the calculation methods and for the media, on reporting values: HF for HF/6-31G(d,p), DFT for DFT/B3LYP/6-31+G(d,p), MP2 for MP2/HF/6-31G(d,p), ‘vac’ for vacuum, ‘chlrf’ for chloroform, ‘actn’ for acetonitrile and ‘aq’ for water.

Detailed information about the data obtained, such as figures showing all the calculated conformers, tables reporting all the values of the computed quantities and also tables showing comparisons of relevant molecular properties, are included in the Supplementary Materials.

## 3. Results

### 3.1. Results In Vacuo

The atom numbering here utilised for the ARZ molecule (Figure 1) maintains the same numbering utilised for the acylphloroglucinol moiety in other studies of ACPLs (7–11), to facilitate comparisons and cross-references.

Given the number of energy-influencing factors, it is important to keep track of relevant geometry features across conformers in a systematic manner, to enable straightforward comparisons [7,8,9,10,11]. Following a practice introduced in previous works [7,8,9,10,11], conformers are denoted by acronyms in which each symbol (letter or number) specifies a certain characteristic, so that the acronym contains complete information about the combination of relevant features specific to the given conformer. The acronyms start with the number denoting the mutual orientation of the moieties (Figure 2). The other characteristics are denoted by letters, whose meanings are listed in Table 1. For the characteristics related to the PHL moiety, the letters are the same as in previous works [7,8,9,10,11]; these characteristics include the position of the first IHB (whether it engages H15 or H17), the orientation of the phenol OHs and the presence of O–H···π interactions between O10H16 or O12H17 and the π-bond of the PRN chain. New letters are introduced to denote each of the possible IMHBs.

**Figure 3:**
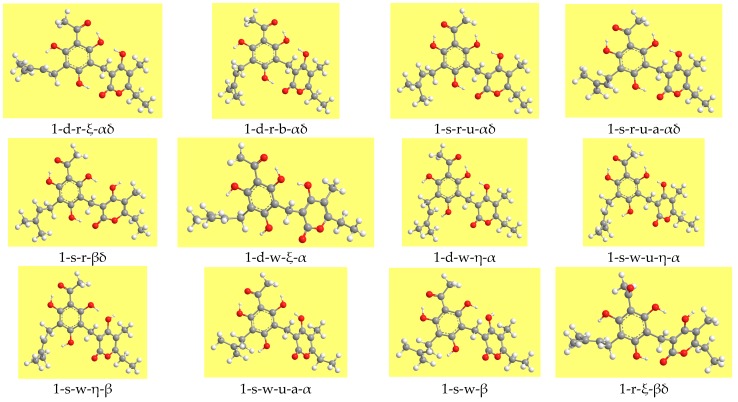
Illustrative images of different types of conformers of the arzanol molecule, considering relevant conformers of the series denoted by the number 1.

Table 2 reports the relative energies of the calculated conformers and Figure 3 shows representative geometries. The ZPE correction is very close for all the conformers and, therefore, the increasing-energy sequence is basically the same for ZPE-corrected relative energies, with only marginal differences for few pairs of conformers. The corrected relative Gibbs free energies (sums of electronic and thermal free energies) show an analogous trend.

Most molecular properties show clear dependence on the characteristics of individual conformers: the position of the first IHB, the orientations of the other phenol OHs in the PHL moiety, and the types and combinations of IMHBs (which are related also to the orientation of the PYR moiety with respect to the PHL moiety). Tables showing the ranges of the values of relevant properties for each conformer-type are included in this text, whereas tables with all the individual values of each property are included in the Supplementary Materials.

In corresponding conformers of the 1 & 2 series, or in corresponding conformers of the 3 & 4 series, the geometry of the PYR moiety is symmetrical with respect to the plane identified by the benzene ring. Although previous studies of ACPLs [30] had shown that the difference between the relative energies of such corresponding conformers is marginal, it was opted to calculate most pairs both to confirm the similarity of the relative energies of corresponding conformers for ARZ, and in view of the fact that—given the flexibility of substituents on the two rings—symmetric orientations of the PYR moiety may not imply symmetric geometries of its substituents; furthermore, the PRN at C5 does not lie on the plane of the benzene ring and, therefore, symmetric orientations of the PYR moiety with respect to this plane do not imply symmetry with respect to PRN. The results show that the energy difference is marginal for all the lowest energy pairs of conformers, but may be non-negligible for some pairs of higher energy conformers. The ranges (kcal/mol) of the energy difference between the two conformers of each pair are 0.0–1.3/HF, 0.0–1.6/MP and 0.0–1.9/DFT. The values of the C3-C9-C17-C18 torsion angle are mostly close-to-opposite (opposite signs and similar absolute values) for the conformers of each pair.

IHBs are the dominant stabilizing factors. Considering the conformers in order of increasing relative energy, the 12 lower energy conformers (with relative energies from 0.0 to 9.3 kcal/mol/DFT) have three IHBs—the first IHB and two IMHBs. The four lowest energy conformers (with relative energies from 0.0 to 2.1 kcal/mol/DFT) also have the O-H···π interaction besides the three O–H···O IHBs. Because of their importance, the characteristics of the various types of IHBs will be analysed in detail in this text.

The mutual orientation of the PHL and PYR moieties determines the types of IMHBs that can be present in a conformer. In the 1 & 2 series, the H27···O8, H15···O26 and H16···O23 IMHBs are possible. In the 3 & 4 series, the H15···O23, H16···O26 and H27···O10 IMHBs are possible.

The possibility of formation of IMHBs influences the preference for the position of the first IHB. While conformers with the H15···O14 first IHB (d-conformers) have lower energy than conformers with the H17···O14 first IHB (s-conformers) in ACPLs that do not form additional IHBs [7,8,9,10,11], in the case of ARZ d-conformers are preferred only in the 1 & 2 series, where the simultaneous presence of H15···O14 and H27···O8 is possible, and s-conformers are preferred in the 3 & 4 series, where O8H15 can form an IMHB (H15···O23) only if it is not engaged in H15···O14. Furthermore, while, for ACPLs that do not form additional IHBs, conformers in which the ortho OH not engaged in the first IHB is oriented towards the acyl group (u-conformers) have higher energy than those in which it is oriented away from the acyl group (non-u conformers), s-u-conformers are preferred to s-conformers in the 1 & 2 series of ARZ, because the ‘upward’ orientation of H15 enables the formation of the H27···O8 IMHB.

An IHB length (H···O distance) is an indication of its strength. Table 3 provides an overview of the ranges of the length of the various IHBs in different conformer-types. The specification of the conformer-type is important because the rest of the molecular geometry influences the characteristics of a given IHB (the IHB length depends on the type of IHB and on the molecular context). For the H15···O14 first IHB, the shortest length pertains to conformers in which it is consecutive and cooperative with the H27···O8 IMHB. Like in ACPLs in general, the length of H15···O14 is somewhat shorter than the length of H17···O14; although this difference is usually more marked when there is a substituent at C3 and no substituent at C5 [7], in the case of ARZ the length difference might be due to the bulkier size of PYR with respect to PRN. The length of H17···O14 shows another trend typical of ACPLs, as it is longer for u-conformers, although these conformers have better energy than non-u ones when H27···O8 is also present (αδ and α conformers).

The length of IMHBs is shorter when two IMHBs are present and increases significantly when only one is present, likely because the presence of two simultaneous IMHBs, besides strengthening the overall bonding between the two moieties, contributes to keep them in a mutual orientation more suitable for IMHB formation. The IMHBs with shortest lengths are those in which the acceptor is sp^2^ O23 (H15···O23 and H16···O23). H15···O23 is shorter in s-w-conformers and longest in s-r conformers; H16···O23 is shorter in d-r and s-r-u-conformers than in s-w conformers. H27···O8 is shorter in d-conformers, confirming that H27···O8 and H15···O14 are cooperative, as they strengthen each other when they are present simultaneously. IMHBs in which O26H27 is the donor (H27···O8 and H27···O10) have shorter lengths than IMHBs in which O26 is the acceptor (H15···O26 and H16···O26).

The calculated IHB length values show the known phenomenon for which DFT tends to overestimate H-bond energies, thus yielding shorter H-bond lengths than experimental ones, while HF tends to underestimate them and might yield lengths slightly longer than experimental values. For ARZ, the difference between HF and DFT results seems somewhat greater for the first IHB (0.130–0.150 Å) than for IMHBs (mostly 0.080–0.130 Å). However, the identification of trends through comparisons of the results of the same method remains valid and the trends highlighted by the HF results and DFT results are similar.

The O-H···π interaction has non-negligible stabilizing effect for ACPLs [9]. This interaction cannot be specified by two atoms (as in the case of IHBs between specific pairs of atoms) and, therefore, it is not possible to speak in terms of H-bond length. However, the distance of the H atom from the two sp^2^ C atoms of the π bond may offer an indication of the interaction strength. Considering the C atom closer to the H of the OH, the H16···C29 distance (Å) in η-conformers is 2.241–2.416/HF and 2.054–2.200/DFT and the H17···C29 distance (Å) in ξ-conformers is 2.264–2.422/HF and 2.086–2.185/DFT.

Additional information on the strength of IHBs can be obtained from the red-shifts (lowering of the vibrational frequency of the donor O-H) that they cause. Table 4 shows the ranges of the red shifts of the four OHs present in the ARZ molecule, considering the type of IHB formed and the molecular context. The first IHBs are by far the strongest. H15···O14 causes the largest red-shifts in conformers in which it is cooperative with H27···O8 (αδ and α conformers). The red shifts of H15···O14 in the absence of cooperativity are mostly comparable with those caused by H17···O14. The trends are consistent with those identified for the first IHB in ACPLs [7], according to which H15···O14 is the stronger first IHB, and the strength of either first IHB decreases for u-conformers with respect to corresponding non-u ones.

The red shift caused by H15···O23 is greatest for γτ and γ conformers and smaller for γε conformers. H15···O26 causes the smallest red shift on the frequency of O8-H15, consistently with the fact that, in this case, the acceptor is an sp^3^ O, whereas it is an sp^2^ O in the previous cases.

The red shifts caused by H27···O8 in αδ and α conformers confirm the cooperativity of H15···O14 and H27···O8; among these, the red shift is greater when a second IMHB is also present (αδ conformers) and smaller for conformers in which it is the only IMHB (α conformers). In the absence of cooperativity (s-u conformers), the red-shift of O26H27 is considerably smaller, remaining however greater when a second IMHB is also present (s-r-u-αδ conformers) and smallest when no other IMHB is present.

The red shift caused by H16···O23 is greatest for αδ conformers, smaller for δ conformers and smallest for βδ conformers. The red shifts caused by H16···O26 are the smallest, suggesting that this IMHB is weaker than the other IMHBs. The O–H···π interaction also causes significant red-shifts (greater than those caused by H16···O26), which confirms its considerable stabilising effect.

The evaluation of the influence of individual factors (including IHBs) through conformers’ comparisons is complicated by the impossibility of isolating one effect at the time. For instance, while the comparison of corresponding d-conformers and s-conformers is straightforward for monomeric ACPLs [7,11], it is not straightforward for ARZ, because the two positions of the first IHB imply different combinations of IMHBs (i.e., significant changes in the molecular context).

Ideally, the energy of IHBs should be evaluated by comparing the energies of the conformer with the IHB and the conformer resulting from its removal through 180° rotation of the donor [31,32,33,34,35,36,37,38,39,40]. However, this evaluation is often complicated by changes in the molecular geometry and intramolecular interactions, brought about by an IHB removal. In the case of ARZ, the changes accompanying the removal of IHBs engaging O8H15 or O10H16 are major, as they often imply changes in the overall IHB pattern. For instance, in d-αδ conformers it is impossible to remove the first IHB by 180° rotation of O8H15 because of the presence of an IMHB in which O8 is the acceptor; in d-conformers in which O8 is not engaged in an IHMB, a 180° rotation of O8H15 would imply the formation of an IMHB between H15 and O23 or O26—a major energy-influencing change preventing the evaluation of the energy of H15⋅⋅⋅O14 as the difference between the energies of the two conformers (with and without H15···O14).

It is possible to obtain some indications on the energy increase caused by the removal of the H17···O14 first IHB because O12H17 is not involved in IMHBs and it is therefore possible to compare conformers with the same IMHB patterns (although they might differ by the presence, or absence, or type of the O-H···π interaction). The removal of H17···O14 causes the following energy increases (kcal/mol): 8.8–9.7/HF, 8.9–9.7/MP2 and 11.3–12.3/DFT when removed from s-r-βδ conformers to give r-ξ-βδ conformers; ≈11/HF,MP2 and 13.5/DFT when removed from s-r-γε conformers to give r-ξ-γε conformers; ≈15.3/HF, 16.1/MP2 and 18.7/DFT when removed from s-w-η-γτ conformers to give w-ξ-γτ conformers; ≈12.9/HF, 13.0/MP2 and 15.4/DFT when removed from s-w-a-γτ conformers to give w-ξ-γτ conformers. The energy increase from s-w-η-γτ to w-ξ-γτ conformers corresponds to lowest interference from other factors, as the only accompanying change is the shift from η-type to ξ-type O–H···π interaction, whose energy difference is marginal (in the other cases, the O–H···π interaction is not present in the conformer with H17···O14 but is present in the conformer resulting from its removal, resulting in greater energy impact).

The removal of an IMHBs causes substantial energy increase, whose magnitude depends on the type of IMHB and on the molecular contexts of the starting conformer and the conformer resulting from its removal. Table 5 shows the ranges of the energy increase on removal of each of the IMHBs, considering the cases in which no other IHBs are formed and analysing the increase according to the type of initial and resulting conformers. The removal of H15···O23 and H15···O26 is possible only if the resulting conformer is a u-conformer; the change from non-u to u-conformer implies an additional energy increase, which, however, is considerably smaller than the increase caused by the removal of the IMHB. The ranges are consistent with the other parameters providing indications on IHB strengths (Table 4 and Table 5) and indicate that the IMHB in which sp^2^ O23 is the acceptor are the strongest and H16···O26 is the weakest.

The removal of both IMHBs causes substantial geometry changes, as the two ring systems, no more bound by the IMHBs, change their mutual orientation so that the planes identified by the two rings become close to perpendicular. Although this mutual orientation is likely to be energetically favourable, the stabilising effect of the IMHBs is much greater, and conformers without IMHBs have high relative energy (the lowest-energy one among them, 2-d-w-η, has a relative energy of 21.5/HF, 23.4/MP2 and 22.3/DFT kcal/mol).

Table 6 reports the ranges of the energy increase caused by the removal of the O-H···π interaction. The removal of H17···π (ξ conformers) causes very close energy-increases, independently of the other characteristics of the conformer, whereas the energy increase for the removal of H16···π (η conformers) shows some variations according to the type of conformer. A further comparison of the strength of the two interactions is offered by d-w conformers, which may form either H17···π or H16···π. The comparison of corresponding conformers (e.g., d-w-ξ-α and d-w-η-α) shows no preference pattern (the ξ conformer may have slightly lower or slightly higher energy than the η conformer); the absolute value of the energy difference is 0.0–1.0/HF, 0.1–1.7/MP2 and 0.0–0.6/DFT.

The effect of the orientation of the phenol OHs cannot be singled out, as changes in the orientation of O8H15 or O10H16 lead to changes in the IHB patterns. For instance, the change of O10H16 from ‘to the right’ (r-conformers) to ‘to the left’ (w-conformers) implies the removal of H16···O23 from 1(2)-d-r-αδ or 1(2)-s-r-u-αδ conformers and the replacement of H16···O26 by H27···O10 in 3(4)-s-r-γε and 3(4)-s-r-ε conformers; thus, the energy difference between conformers with different orientations of H16 is largely due to changes in IHB patterns. Similarly, the orientation of H15, when not engaged in H15···O14, determines the types of IMHB present: H15···O23 or H15···O26 in s-r and s-w-conformers, H27···O8 in 1(2)-s-r-u or 1(2)-s-w-u conformers, and the absence of an IMHB involving O8H15 in 3(4)-s-r-u or 3(4)-s-w-u conformers. A comparison between non-u and u-conformers is only possible for O12H17 in d-conformers with no H17···π interaction (e.g., d-r-αδ and d-r-u-αδ), because then the shift of O12H17 to ‘upward’ orientation does not bring about other energy-influencing changes. The energy increase caused by this shift is 4.1–4.4/HF, 3.1–3.5/MP2 and 3.2–3.5/DFT for d-r-αδ and d-r-δ conformers; 3.2–3.5/HF, 2.1–2.5/MP2 and 2.4–2.6/DFT for d-r-ε conformers; 2.2–2.5/HF, 1.1–1.4/MP2 and 1.0–1.3/DFT for d-w-η-α and d-w-η conformers; and 1.1–1.5/HF, 0.0–0.3/MP2 and 0.3–0.4/DFT for d-w-η-τ conformers.

The dipole moment may play some roles in biological activities; for instance, anthracyclines exert anticancer activity only if their dipole moment is comparatively low, and the activity decreases as the dipole moment increases [41]. It may also be a pertinent QSAR descriptor when the activity depends on a molecule’s polarity. Table 7 shows the ranges of the dipole moment of the various types of ARZ conformers in vacuo. The orientation of the OH groups has major influence on the dipole moment. The highest dipole moments pertain to s-r-βδ conformers, in which three of the four OHs are roughly oriented towards the same direction. The lowest dipole moments pertain to d-r-αδ conformers, in which the OHs are oriented in a way that largely cancels their contributions.

**Figure 4:**
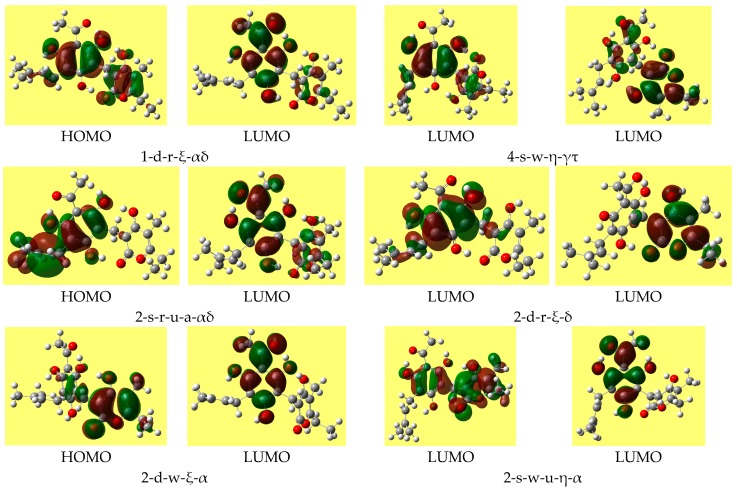
Representative shapes of the frontier molecular orbitals of arzanol, from DFT/B3LYP/6-31+G(d,p) results.

The energy difference between the frontier orbitals (HOMO, highest occupied molecular orbital, and LUMO, lowest unoccupied molecular orbital) is an important descriptor (including a QSAR descriptor), as it is related to molecular reactivity. DFT may give HOMO-LUMO gaps that differ considerably from those of other methods and that are often non-realistic. Therefore, the HF values of the gap are here considered as reference. Table 8 reports the ranges of the values of the HOMO-LUMO energy gap for the various types of conformers; the values do not differ substantially for different conformer types. Trend-identification is similar in the HF and DFT results. The shapes of the frontier orbitals (very similar in the HF and DFT results) vary with the types of conformers, and have similar features for conformers of analogous types. Figure 4 shows representative shapes, differing by the distribution over different regions of the molecule, according to the conformers’ charactering features.

The mutual orientation of the two moieties is largely determined by the IMHB patterns. The simultaneous presence of two IMHBs (on the two sides of the methylene bridge) determines a geometry in which the situation of the rings of the two moieties could be roughly matched by a large-radius cylindrical shape. When only one IMHB is present, there is a distortion of the orientation of the plane of the α-pyrone ring with respect to that of the benzene ring. When no IMHB is present, the planes of the two rings tend to become close-to-perpendicular, as is the usual tendency of aromatic rings.

The C3-C9-C17 bond angle is somewhat greater than the expected tetrahedral value, likely because of the bulk of the two moieties joint by the methylene bridge. For the 14 lower-energy conformers, the angle is 117.0–117.1°/HF and 116.2–117.0°/DFT. Greater fluctuations are observed for some higher energy conformers, still remaining within the 114.0–117.1°/HF and 113.9–117.0°/DFT ranges.

### 3.2. Results in Solution

Of the three solvents considered (chloroform, acetonitrile and water), acetonitrile has the highest dipole moment. However, its effect on a number of molecular properties of the solute (relative energies, dipole moment, etc.) is intermediate between that of chloroform and that of water, as if its polarity were intermediate. In the rest of the text, wordings of the type “as the solvent polarity increases” refer to the chloroform-acetonitrile-water sequence in terms of the effects of these solvents on the properties considered, and not in terms of the actual values of the dipole moments of acetonitrile and water.

Calculations in solution were performed both with fully relaxed geometry and as single point (SP) calculations on the in-vacuo-optimised geometries. A number of calculations with fully relaxed geometry did not converge. The results of those that converged provide indications on the effect of the solvent on the conformers’ geometries and enable comparisons to assess the SP results. Since more SP results are available than full re-optimisation results, the ranges provided in the next sections refer to SP results, unless otherwise specified. All the results of full reoptimisation calculations (as well as those of SP calculations) are included in the Supplementary Materials.

The conformers’ relative energy decreases in solution. The decrease is greater for conformers with higher relative energy, because the stabilisation by the solvent is usually greater for higher-energy conformers. Thus, the energy gap between conformers decreases as the solvent polarity increases and decreases to a greater extent for higher energy conformers. Table 9 shows a panoramic of the decrease-ranges analysed in terms of conformers’ type and referred to the relative energies in vacuo. Besides the six conformers with relative energy ≤3.5 kcal/mol in vacuo (1-d-r-ξ-αδ, 2-d-r-ξ-αδ, 3-s-w-η-γτ, 4-s-w-η-γτ, 2-d-r-αδ, 1-d-r-b-αδ), two more conformers (4-s-w-a-γτ and 3-s-w-a-γτ) have relative energy ≤3.5 kcal/mol in water solution and can, therefore, be included among those that might be potentially responsible for the molecule’s biological activity.

A comparison of the parameters of the IHBs for the conformers whose reoptimisation in solution converged shows that the changes in the H-bond length are mostly small. The length of the first IHB often shows a slight decrease as the solvent polarity increases: e.g., 1.564/vac, 1.549/chlrf, 1.541/actn, 1.536/aq for H15···O14 for 4-d-w-η-τ or 1.553/vac, 1.545/chlrf, 1.541/actn, 1.540/aq for H17···O14 in 2-s-r-a-βδ. In some cases, the length decreases in the other two solvents and slightly increases in water (e.g., 1.507/vac, 1.499/chlrf, 1.496/ actn, 1.502/ aq for H15⋅⋅⋅O14 in 1-d-w-ξ-α) or increases as the solvent polarity increases (e.g., 1.482/vac, 1.486/chlrf, 1.488/actn, 1.499/aq for H15···O14 in 1-d-r-ξ-αδ). Changes in the O···O distance and in the bond angle are marginal.

The bond length of the IMHBs shows marginal fluctuations or decreases as the solvent polarity increases for those IMHBs in which the donor pertains to the PHL moiety. Examples of decrease are 1.691/vac, 1.680/chlrf, 1.675/actn, 1.680/aq for H15···O23 in 4-s-w-a-γτ; 1.751/vac, 1.713/chlrf, 1.701/actn, 1.691/aq for H16···O23 in 1-s-r-βδ; 1.939/vac, 1.917/chlrf, 1.905/actn, 1.882/aq for H15···O26 in 2-s-r-a-βδ; 1.954/vac, 1.926/chlrf, 1.916/actn, 1.894/aq for H16⋅⋅⋅O26 in 4-s-r-a-γε. For IMHBs in which the donor is H27, the bond length may decrease (e.g., 1.769/vac, 1.747/chlrf, 1.739/actn, 1.730/aq for H27···O8 in 1-d-w-ξ-α, or 1.842/vac, 1.809/chlrf, 1.793/actn, 1.777/aq for H27···O10 in 4-d-w-η-τ) or, less frequently, increase (e.g., 1.760/vac, 1.762/chlrf, 1.763/actn, 1.766/aq for H27⋅⋅⋅O8 in 1-d-r-ξ-αδ or 1.797/vac, 1.912/actn, 1.939/aq for H27···O10 in 3-s-w-η-γτ). 

The PCM optimisation algorithm is not designed to break IHBs. However, in two cases the bond length, which decreases slightly in the other two solvents, increases dramatically in water solution and the bond angle decreases dramatically, suggesting that the given IMHB does likely break. It is the case of H16···O26 in 4-d-r-ξ-ε (bond length 1.963/vac, 2.727/aq; O···O distance 2.830/vac, 3.119/aq; bond angle 147.4/vac, 104.6/aq) and in 4-d-r-ε (bond length 1.981/vac, 2.657/aq; O···O distance 2.841/vac, 3.075/aq; bond angle 146.4/vac, 106.2/aq). An intermediate situation appears with H27···O8 in 2-s-w-u-a-α, where the greatest change concerns the bond angle (bond length 1.885/vac, 2.120/aq; O···O distance 2.771/vac, 2.812/aq; bond angle 149.7/vac, 127.4/aq). All the other values suggest that the other IHBs are maintained in water solution. Previous studies on ACPLs [8,42] suggest that the first IHB is maintained in solution, including water solution. As for the IMHBs of ARZ, their permanence is suggested not only by their parameters in solution, but also by the fact that the geometry of several conformers does not favour solvent molecules’ access to the atoms forming them. A study of adducts of ARZ with explicit water molecules can be expedient to verify whether its IMHBs are maintained in water solution. Given the high number of conformers and the high number of centres in the ARZ molecule to which water molecules can bind, the number of adducts to be considered for a reasonably informative study is high; therefore, such study will be the object of a separate work.

When the O-H···π interaction is present, the distance between the donor H16 or H17 and the C29 and C30 atoms of the π bond in PRN mostly decreases as the solvent polarity increases (e.g., 2.104/vac, 2.079/chlrf, 2.071/actn, 2.054/aq for H17···C29 in 1-d-r-ξ-αδ and 2.110/vac, 2.370/chlrf, 2.019/actn, 2.033/aq for H16···C29 in 4-d-w-η-τ). It increases only in the case of 3-s-w-η-γτ, where the H16···C29 distance is 2.071/vac, 2.245/actn, 2.270/aq. The values would suggest the permanence of the O-H···π interaction in all the solvents considered, including water. The geometries of the η and ξ-conformers would also appear compatible with a permanence of the interaction. The consideration of adducts with explicit water molecules would be critical to provide concluding information about the fate of the O-H···π interaction in water solution, within QM approaches.

The free energy of solvation (ΔG_solv_) has small magnitude in chloroform, with positive values (kcal/mol) for the 12 lower energy conformers (0.6–2.0) and negative values for the higher energy ones (−2.6 to −0.1, mostly −1.9 to −0.1). It has only positive values in acetonitrile (6.6–8.2 for the 12 lower energy conformers and 2.0–6.5 for the higher energy ones) and only negative values in water, with their magnitude mostly increasing as the conformers’ relative energy increases (−15.1 to −11.8 for the 12 lower energy conformers and −23.9 to −16.7 for the higher energy ones). Table 10 provides an overview of the ranges of ΔG_solv_ analysed in terms of conformer types. Comparison of the values from full optimisation PCM calculations with the SP results shows that the former are smaller by 0.3–0.7/chlrf, 0.3–1.3/actn and 0.3–1.3/aq. Trend identification is similar in the two sets of results. The values suggest that ARZ might have a certain solubility in water, likely because of its numerous H-bond donor and acceptor sites. In the PCM method, ΔG_solv_ is the sum of an electrostatic and a non-electrostatic components (ΔG_solv_ = G_el_ + G_non-el_). G_el_ is negative for all the conformers and in all the solvents, and its magnitude is greater for polar solvents and mostly increases with the conformer’s relative energies; the ranges in the SP results, considered from lower to higher energy conformers, are (−3.8 to −9.2)/chlrf, (−5.3 to −12.7)/actn and (−11.8 to −23.9)/aq.

The dipole moment increases as the solvent polarity increases, and the increase is greater for conformers with greater dipole moment in vacuo. For SP PCM calculation, the increase is due solely to the solvent effect, because the geometry of the conformers remains the same as in vacuo. The percent increase with respect to in vacuo (SP results) is 12.3–23.0%/chlrf, 15.3–37.3%/actn, 14.8–48.0%/aq. The values from full optimisation calculations are slightly greater than those from SP calculations, with few exceptions in water for high energy conformers. Full optimisation takes into account the geometry adjustment of the solute molecule in response to the solvent (polarisation effect of the solvent on the solute molecule). The difference (debye) between the two sets of results is 0.07–0.69/chlrf, 0.11–1.02/actn, 0.11–1.27/aq; the percentage by which the SP value is smaller than the full-optimisation value is 2.75–6.02%/chlrf, 1.23–9.70%/actn, 3.97–14.65%/aq. Trend identification is similar in the two sets of results.

**Figure 5:**
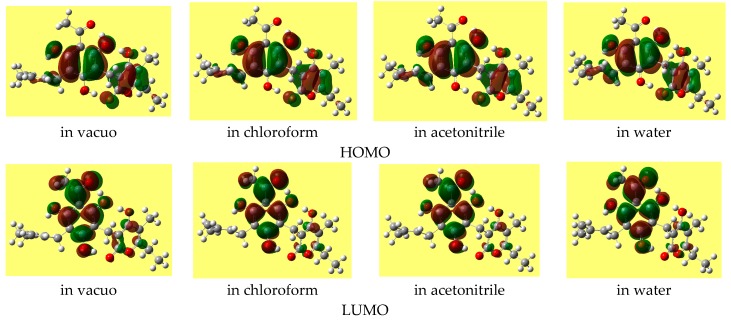
Frontier orbitals of the lowest energy conformer (1-d-r-ξ-αδ) in different media. DFT/B3LYP/6-31+G(d,p) results from calculations with fully relaxed geometry in all the media.

The HOMO-LUMO energy gap often increases as the solvent polarity increases (except for some of the lowest energy conformers, for which it decreases slightly). The shape of the HOMO of a given conformer remains fairly similar in all the media, whereas the shape of the LUMO may show noticeable differences. Figure 5 shows the shapes of the frontier orbitals of the lowest energy conformer in different media.

## 4. Discussion and Conclusions

This study aimed at providing detailed information on the molecular properties of ARZ and on the factors influencing its conformational preferences. The importance of detailed information about biologically active molecules relates to the fact that their biological activity may be associated with the finest details of their molecular structure and properties [41]. Although only conformers with sufficiently low relative energy may be responsible for the biological activity, it was decided to also calculate higher energy conformers in order to assess the effect of individual energy-influencing factors, above all intramolecular H-bonding. 

The results highlight the stabilizing effect of the first IHB, of the IMHBs, of the cooperativity between an IHB and an IMHB, and of the O–H···π interaction, thus showing that IHB patterns are the most important stabilizing factors. The trends of other factors are consistent with the general trends identified for ACPLs [7,9,10,11] when they do not interplay with IHB patterns; e.g., the effect of the orientation of the phenol OHs is analogous to that identified for ACPLs for those OHs that are not engaged in IMHBS.

The results in solution are also consistent with the trends identified for ACPLs [8,42] and suggest that the first IHB and most of the IMHBs do not break in a water solution. A separate study of adducts of arzanol with explicit water molecules is planned to verify this inference. 

## Figures and Tables

**Table 1:** Symbols utilised to specify geometrical characteristics in the acronyms denoting the conformers. The symbols d, s, r, w, u, η and ξ have the same meanings as in other studies on ACPLs [7,8,9,10,11].

Symbol	Meaning
d	The H15···O14 first IHB is present
s	The H17···O14 first IHB is present
r	H16 is oriented to the side of the α-pyrone ring
w	H16 is oriented to the side of the prenyl chain
u	H15 or H17, not engaged in the first IHB, is oriented toward the acyl chain
η	H16 forms O-H···π interaction with the double bond of the prenyl chain
ξ	H17 forms O-H···π interaction with the double bond of the prenyl chain
a	There is no O-H···π interaction, and the prenyl chain is oriented ‘upwards’
b	There is no O-H···π interaction, and the prenyl chain is oriented ‘downwards’
α	The H27···O8 intermoiety hydrogen bond is present
β	The H15···O26 intermoiety hydrogen bond is present
γ	The H15···O23 intermoiety hydrogen bond is present
δ	The H16···O23 intermoiety hydrogen bond is present
ε	The H16···O26 intermoiety hydrogen bond is present
τ	The H27···O10 intermoiety hydrogen bond is present

**Table 2:** Relative energies of the calculated conformers of arzanol from the results of different calculation methods in vacuo. The calculation methods are denoted with the following acronyms: HF for HF/6-31G(d,p), MP2 for MP2/6-31G(d,p)/HF/6-31G(d,p) and DFT for DFT/B3LYP/6-31+G(d,p). The table lists all the conformers that optimise to geometries with the same IHB patterns with both HF and DFT. The conformers are listed in order of increasing energy referred to the DFT results.

Conformer	Relative Energy (kcal/mol)			Conformer	Relative Energy (kcal/mol)		
	HF	MP2	DFT		HF	MP2	DFT
1-d-r-ξ-αδ	0.0	0.0	0.0	2-s-w-η-β	16.4	17.6	17.5
2-d-r-ξ-αδ	0.1	0.0	0.0	4-d-w-u-η-τ	17.1	17.0	17.7
3-s-w-η-γτ	1.3	1.5	2.0	1-s-w-η-β	16.3	17.3	17.8
4-s-w-η-γτ	1.6	1.7	2.1	1-s-r-u-δ	17.3	17.9	17.9
2-d-r-αδ	2.1	2.3	2.5	2-s-r-u-b-δ	17.4	17.8	18.0
1-d-r-b-αδ	2.1	2.3	2.5	2-s-w-u-η-α	17.2	17.7	18.3
4-s-w-a-γτ	3.9	4.6	5.3	3-d-r-ξ-ε	17.1	18.1	18.4
3-s-w-a-γτ	3.9	4.6	5.3	3-d-w-η-τ	16.5	17.7	18.7
2-d-r-u-αδ	6.5	5.8	6.0	3-s-w-u-a-τ	17.6	17.8	18.9
1-d-r-u-b-αδ	6.5	5.8	6.0	1-s-w-u-a-α	18.1	18.6	18.9
1-s-r-u-αδ	9.0	8.5	9.2	4-d-r-ξ-ε	17.4	18.4	18.9
2-s-r-u-a-αδ	9.0	8.5	9.2	3-d-w-u-η-τ	17.6	17.7	19.0
2-s-r-u-b-αδ	9.0	8.4	9.2	3-d-w-ξ-τ	17.1	18.5	19.3
1-s-r-u-a-αδ	9.1	8.6	9.3	2-s-w-a-β	17.9	19.3	19.7
1-d-r-ξ-δ	10.3	11.7	11.2	1-s-w-β	18.2	19.5	20.2
2-d-r-ξ-δ	10.5	11.9	11.2	2-s-w-u-a-α	18.7	19.3	20.6
3-s-w-η-γ	10.5	12.0	11.6	4-w-ξ-γτ	16.7	17.7	20.7
4-s-w-η-γ	10.8	12.4	11.9	3-w-ξ-γτ	16.9	17.7	20.7
4-s-r-b-γε	10.4	11.5	12.0	3-d-r-b-ε	19.2	20.4	20.7
3-s-r-γε	10.5	11.5	12.0	4-d-r-ε	19.4	20.7	21.4
4-s-r-a-γε	10.5	11.6	12.1	3-d-w-u-a-τ	20.3	21.0	22.0
3-s-r-b-γε	10.5	11.6	12.1	2-d-w-η	21.5	23.4	22.3
2-s-r-a-βδ	11.5	12.5	12.8	1-d-w-η	22.1	23.8	22.7
1-s-r-βδ	11.6	12.5	12.8	1-d-w-ξ	22.0	23.5	22.7
2-s-r-b-βδ	11.5	12.4	12.9	2-d-w-ξ	22.3	23.9	22.9
1-s-r-βδ′	11.6	12.5	13.0	3-d-r-u-b-ε	22.3	22.5	23.1
2-d-r-δ	12.5	14.1	13.6	1-d-w-η′c	22.6	24.2	23.1
1-d-r-b-δ	12.5	14.0	13.7	2-d-w-u-η	23.8	24.5	23.3
1-d-w-ξ-α	12.8	13.5	13.7	2-s-w-u-η	23.7	24.5	23.4
2-d-w-ξ-α	12.9	13.6	13.8	4-d-w-η	22.9	24.6	23.6
1-d-w-η-α	13.2	13.9	14.0	4-d-w-ξ	23.0	24.5	23.6
3-s-w-γ	12.6	14.2	14.0	3-d-w-ξ	23.2	24.8	23.9
2-d-w-η-α	13.9	15.3	14.0	1-d-w-u-η	24.5	25.0	24.0
4-s-w-γ	12.5	14.3	14.1	4-d-r-u-ε	22.8	23.1	24.0
1-d-w-u-η-α	15.5	15.1	15.2	3-d-w-η	23.3	25.0	24.0
2-d-w-u-η-α	16.4	16.7	15.3	2-r-ξ-βδ	20.3	21.4	24.2
4-s-w-u-η-τ	15.9	15.9	15.6	4-d-w-u-η	24.7	25.2	24.3
3-s-w-u-η-τ	15.3	15.2	15.7	1-r-ξ-βδ	21.2	22.3	25.1
1-s-w-u-η-α	16.6	16.2	16.4	4-r-ξ-γε	21.4	22.6	25.5
1-d-r-u-b-δ	16.6	17.2	16.9	3-r-ξ-γε	21.6	22.7	25.6
2-d-r-u-δ	16.6	17.2	16.9	1-s-w-u	25.2	26.0	25.6
4-d-w-η-τ	15.6	16.6	17.3	1-d-w-u	26.1	26.9	26.1
4-d-w-ξ-τ	15.8	16.6	17.5				

**Table 3:** Ranges of the length of the IHBs in the calculated conformers of arzanol. HF/6-31G(d,p) and DFT/B3LYP/6-31+G(d,p) results in vacuo, respectively denoted as HF and DFT in the column headings. The types of conformers are characterised by the features that prove relevant in relation to the ranges of the given IHB, i.e., the first IHB and the orientations of the other phenol OHs (under the PHL heading) and the types and combinations of IMHB (under the IMHB heading). When only two values are available for a given type, they are both reported, separated by a comma, and in the same sequence in the HF and DFT columns.

IHB and Conformer Type		Length Range (Å)		IHB and Conformer Type		Length Range (Å)	
PHL	IMHB	HF	DFT	PHL	IMHB	HF	DFT
H15···O14				H15···O26			
d-r	αδ	1.631–1.633	1.482––1.483	s-r, r	βδ	2.013–2.063	1.936–1.969
d-r-u	αδ	1.657, 1.662	1.501, 1.502	s-w	β	2.032–2.050	1.934–1.947
d-r	δ	1.669–1.673	1.542–1.543	H15···O23			
d-r-u	δ	1.700, 1.705	1.554, 1.556	s-w	γτ	1.792–1.796	1.691–1.700
d-r	ε	1.673–1.692	1.546–1.562	s-w	γ	1.808–1.812	1.676–1.684
d-r-u	ε	1.713, 1.726	1.560, 1.574	w-ξ	γτ	1.817, 1.823	1.720, 1.733
d-w	α	1.638–1.655	1.498–1.507	s-r	γε	1.831–1.835	1.730–1.734
d-w-u	α	1.671, 1.677	1.523, 1.524	r	γε	1.860, 1.872	1.754, 1.767
d-w	τ	1.685–1.700	1.555–1.571	H16···O23			
d-w-u	τ	1.728–1.738	1.574–1.586	d-r, d-r-u, s-r-u	αδ	1.805–1.811	1.697–1.704
d-w	none	1.670–1.697	1.546–1.566		δ	1.823–1.832	1.680–1.694
d-w-u	none	1.713–1.727	1.564–1.578	s-r	βδ	1.847–1.851	1.745–1.752
H17···O14				r	βδ	1.854, 1.874	1.752, 1.760
s-w	γτ	1.658–1.663	1.528–1.533	H16···O26			
s-w	γ	1.661–1.667	1.530–1.538	s-r	γε	2.031–2.040	1.952–1.958
s-w-u	α	1.704–1.713	1.557–1.565	r	γε	2.036, 2.056	1.954, 1.957
s-r	γε	1.673–1.676	1.545–1.548	d-r	ε	2.007–2.078	1.924–1.982
s-r	βδ	1.677–1.680	1.553–1.557	H27···O10			
s-r-u	αδ	1.722–1.724	1.576–1.582	s-w, w	γτ	1.897–1.917	1.794–1.822
H27···O8				s-w-u	τ	1.951–2.017	1.820–1.884
d-r	αδ	1.786–1.881	1.760–1.767	d-w, d-w-u, w	τ	1.952–2.049	1.840–1.931
d-r-u	αδ	1.884, 1.887	1.774, 1.774				
s-r-u	αδ	1.907–1.909	1.813–1.814				
d-w	α	1.812–1.942	1.761–1.776				
d-w-u	α	1.913, 1.953	1.884, 1.786				
s-w-u	α	1.951–2.072	1.837–1.885				

**Table 4:** Ranges of the red-shifts of the calculated vibrational frequencies (harmonic approximation) of the O-H bonds when they are engaged in intramolecular hydrogen bonds (IHB). DFT/B3LYP/6-31+G(d,p) results in vacuo. The frequency values have been scaled by the factor 0.964, recommended for DFT/B3LYP/6-31+G(d,p) calculations [22]. The red shifts are evaluated with reference to the average frequency of the same OH when not engaged in IHBs, taken from the conformers in which it is free. The types of conformers are characterised by the features that prove relevant in relation to the ranges of the given IHB, i.e., the first IHB and the orientations of the other phenol OHs (under the PHL heading) and the types and combinations of IMHBs (under the IMHB heading). When only two values are available for a given type, they are both reported, separated by a comma. The meaning of a letter being in parentheses in the acronym denoting a conformer, or of the presence of two letters corresponding to the same feature (e.g., ξ/η) is explained in detail in the caption of Table 5.

OH Group and IHB Concerned	Conformer Type		Red Shift Range (cm^−1^)	OH Group and IHB Concerned	Conformer Type		Red Shift Range (cm^−1^)
	PHL	IMHB			PHL	IMHB	
**O8–H15**				**O10–H16 (cont)**			
H15···O14	d-r	αδ	1130–1139	H16···π	s-w-η	γτ	273, 357
	d-r-u	αδ	1016, 1020		s-w-η	γ	165–183
	d-r	δ	841–844		s-w-η	β	177, 187
	d-r-u	δ	766, 769		s-w-u-η	α	196, 204
	d-r	ε	782–824		s-w-u-η	τ	242, 268
	d-r-u	ε	707, 741		s-w-u-η	none	182–187
	d-w	α	1012–1041	**O12–H17**			
	d-w-u	α	912, 916	H17···O14	s-w-(η)	γτ	841–855
	d-w	τ	746–780		s-w-η	γ	851–852
	d-w-u	τ	668–681		s-w	γ	828, 837
	d-w	none	759–815		s-w-η	β	812, 813
	d-w-u	none	694–730		s-w	β	799, 803
H15···O23	s-w	γτ	578–586		s-w-u	α	747–771
	s-w	γ	520–531		s-w-u-(η)	τ	765–784
	w-ξ	γτ	492, 512		s-w-u-(η)	none	773–784
	s-r	γε	431–435		s-r	γε	769–788
	r	γε	366, 381		s-r	βδ	741–752
H15⋅⋅⋅O26	s-r	βδ	113–114		s-r-u	αδ	697–706
	r	βδ	89, 99		s-r-u	δ	704, 705
	s-w	β	127–132	H17···π	d-r-ξ	αδ	154, 210
**O10–H16**					d-r-ξ	δ	186, 188
H16···O23	d-r	αδ	565–570		d-r-ξ	ε	177, 180
	d-r	δ	492–502		d-w-ξ	α	172, 178
	d-r-u	αδ	558, 561		d-w-ξ	τ	153, 162
	d-r-u	δ	490, 499		d-w-ξ	none	136–151
	s-r	βδ	357–365		r-ξ	βδ	149, 156
	s-r-u	αδ	521–526		r-ξ	γε	127, 133
	s-r-u	δ	465, 478		w-ξ	γτ	106, 116
	r	βδ	345, 356	**O26–H27**			
H16···O26	s-r	γε	66–71	H27···O8	d-r	αδ	411–421
	r	γε	61, 63		d-r-u	αδ	389, 392
	d-r	ε	85–104		s-r-u	αδ	279–283
	d-r-u	ε	82, 103		d-w-ξ/η	α	322–341
H16···π	d-w-η	α	142, 146		d-w-u-η	α	302, 316
	d-w-η	τ	174, 182		s-w-u	α	156–213
	d-w-η	none	118–124	H27···O10	s-w, w	γτ	264–298
	d-w-u-η	α	185, 193		s-w-u	τ	200–248
	d-w-u-η	τ	250, 254		d-w	τ	125–188
	d-w-u-η	none	165–171		d-w-u	τ	134–202

**Table 5:** Ranges of the energy increase caused by the removal of one intermoiety hydrogen bond (IMHB), according to the type of starting and resulting conformers. The calculation methods are denoted with the following acronyms: HF for HF/6-31G(d,p), MP2 for MP2/6-31G(d,p)/HF/6-31G(d,p) and DFT for DFT/B3LYP/6-31+G(d,p). When only two values are available for a given type, they are both reported, separated by a comma, and in the same sequence in the HF, MP2 and DFT columns. When the two values of a range are identical, it indicates that at least three coinciding values were determined. When a symbol within the acronym denoting a conformer is inserted in parentheses, it means that the range is valid both when the characteristic denoted by that symbol is present and when it is not; for instance, the acronym d-r-(ξ)-αδ indicates that the given range applies both to the d-r-ξ-αδ conformers and to the d-r-αδ conformers. An acronym with both symbols referred to a given characteristics informs that the range is valid for both types of conformers; e.g., d-w-ξ/η-τ indicates that the given range applies both to the d-w-ξ-τ conformers and to the d-w-η-τ conformers.

IMHB Considered	Type of Starting Conformer	Type of Resulting Conformer	Range of Energy Increase (kcal/mol)		
			HF	MP2	DFT
H27···O8	d-r-(ξ)-αδ	d-r-(ξ)-δ	10.3–10.4	11.7–11.8	11.1–11.2
	d-r-u-αδ	d-r-u-δ	10.0, 10.1	11.4, 11.5	10.9, 10.9
	d-w-ξ-α	d-w-ξ	9.2, 9.4	10.1, 10.3	9.0, 9.1
	d-w-η-α	d-w-η	7.7, 9.0	8.1, 9.8	8.3, 8.8
	d-w-u-η-α	d-w-u-η	7.4, 9.1	7.8, 9.9	8.0, 8.7
	s-r-u-αδ	s-r-u-δ	8.3, 8.4	9.4, 9.4	8.7, 8.8
	s-w-u-η-α	s-w-u-η	6.5	6.8	5.1
H15···O23	s-r-γε	s-r-u-ε	11.1–11.9	10.3–10.4	
	s-w-(η)-γτ	s-w-u-(η)-τ	13-7–14.5	13.3–14.5	13.5–13.8
	3-s-w-γ	3-s-w-u	12.3	11.4	
H15···O26	s-r-βδ	s-r-u-δ	5.8, 5.8	5.3, 5.4	5.0, 5.2
	s-w-(η)-β	s-w-u-(η)	6.9–7.3	6.5–7.2	5.3–5.9
H16···O23	d-r-ξ-αδ	d-w-ξ-α	12.8–12.8	13.5–13.6	13.8–13.8
	d-r-αδ	d-w-η-α	11.1–11.8	11.6–13.0	11.5–14.0
	d-r-u-αδ	d-w-u-η-α	9.0	9.3	9.2
	s-r-u-αδ	s-w-u-α	9.1, 9.7	10.0, 10.9	9.6, 11.3
	s-r-βδ	s-w-β	6.4, 6.7	6.8, 7.0	7.0, 7.4
H16···O26	d-r-ξ-ε	d-w-ξ	5.6, 6.1	6.1, 6.6	4.7, 5.5
	s-r-γε	s-w-γ	2.0–2.1	2.6–2.7	1.9–2.0
	s-r-u-ε	s-w-u	2.5–3.2	3.2–3.8	
H27···O10	s-w-(η)-γτ	s-w-(η)-γ	8.7–9.3	9.6–10.5	8.7–9.6
	d-w-ξ/η-τ	d-w-ξ/η	6.1–6.8	6.2–7.3	4.6–5.3

**Table 6:** Ranges of the energy increase caused by the removal of the O-H···π interaction, according to the type of starting and resulting conformers. The calculation methods are denoted with the following acronyms: HF for HF/6-31G(d,p), MP2 for MP2/6-31G(d,p)/HF/6-31G(d,p) and DFT for DFT/B3LYP/6-31+G(d,p). When only two values are available for a given type, they are both reported, separated by a comma, and in the same sequence in the HF, MP2 and DFT columns.

O-H···π Interaction	Type of Starting Conformer	Type of Resulting Conformer	Range of Energy Increase (kcal/mol)		
			HF	MP2	DFT
H17···π	d-r-ξ	d-r	2.0–2.1	2.2–2.3	2.4–2.5
H16···π	s-w-η-β	s-w-β	1.5, 2.0	≈2.0	2.5, 2.6
	s-w-η-γτ	s-w-a-γτ	2.3, 2.6	3.0, 3.1	3.2, 3.3
	s-w-η-γ	3-s-w-a-γ	2.0	2.2	2.4
	s-w-u-η-τ	s-w-u-a-τ	2.3, 2.5	2.6, 3.1	≈3.1
	s-w-u-η-α	s-w-u-a-α	1.4, 1.6	1.6, 2.0	2.3–2.6
	d-w-u-η-(τ)	d-w-u-a-(τ)	1.6–2.6	1.9–3.3	2.2–3.1

**Table 7:** Ranges of the dipole moment (debye) of the calculated conformers of arzanol. HF/6-31G(d,p) and DFT/B3LYP/6-31+G(d,p) results in vacuo, respectively denoted as HF and DFT in the column headings. The types of conformers are characterised by the features that prove relevant in relation to the ranges of the given dipole moment, i.e., the first IHB and the orientations of the other phenol OH (under the PHL heading) and the types and combinations of IMHB (under the IMHB heading). When only two values are available for a given type, they are both reported, separated by a comma, and in the same sequence in the HF and DFT columns.

Conformer Type		Dipole Moment Range (debye)		Conformer Type		Dipole Moment Range (debye)	
PHL	IMHB	HF	DFT	PHL	IMHB	HF	DFT
d-r	αδ	1.71–2.91	2.10–3.06	s-w	γ	10.63–11.29	11.07–11.89
d-r	δ	4.22–5.14	4.50–5.70	s-w	β	7.33–8.62	7.47–8.43
d-r	ε	9.16–9.93	9.45–10.33	s-w-u-η	α	3.08, 3.41	2.88, 2.98
d-r-u	αδ	3.59, 3.93	3.80, 3.57	s-w-u	α	3.81, 4.05	4.25, 3.95
d-r-u	δ	6.82, 7.02	6.97, 7.13	s-w-u	τ	3.37–4.06	3.37–4.56
d-r-u	ε	7.18, 7.18	7.08, 7.40	s-w-u	none	6.07–7.33	5.76–6.69
d-w-η	α	4.63, 6.79	7.22, 7.10	s-r	γε	12.42–12.81	12.78–13.25
d-w-ξ	α	6.94, 7.51	7.70, 8.07	s-r	βδ	13.29–13.59	13.39–13.76
d-w	τ	9.87–10.84	10.51–11.54	s-r-u	αδ	7.80–7.96	7.81–8.01
d-w	none	7.34–11.14	8.35–11.12	s-r-u	δ	11.59, 11.73	11.88, 12.06
d-w-u-η	α	3.89, 6.17	6.47, 6.33	s-r-u	ε	8.21–8.51	
d-w-u	τ	6.77–8.24	7.11–9.03	w-ξ	γτ	9.21, 10.66	9.07 10.49
d-w-u	none	7.29–8.10	7.46–8.66	r-ξ	βδ	7.12, 10.02	6.87, 9.75
s-w-η	γτ	7.27, 7.66	7.83, 8.06	r	γε	12.17, 14.21	12.35, 14.21
s-w	γτ	6.74, 6.77	6.92, 7.00				

**Table 8:** Ranges of the HOMO-LUMO energy difference (kcal/mol) of the calculated conformers of arzanol. HF/6-31G(d,p) and DFT/B3LYP/6-31+G(d,p) results in vacuo, respectively denoted as HF and DFT in the column headings. The types of conformers are characterised by the features that prove relevant in relation to the ranges of the energy gap, i.e., the first IHB and the orientations of the other phenol OHs (under the PHL heading) and the types and combinations of IMHBs (under the IMHB heading). When only two values are available for a given type, they are both reported, separated by a comma, and in the same sequence in the HF and DFT columns.

Conformer Type		HOMO-LUMO Gap Range (kcal/mol)		Conformer Type		HOMO-LUMO Gap Range (kcal/mol)	
PHL	IMHB	HF	DFT	PHL	IMHB	HF	DFT
d-r	αδ	256.1–256.7	101.1–101.8	d-w-η/ξ	τ	248.3–253.7	93.1–98.7
d-r-u	αδ	249.0, 248.7	93.4, 93.8	d-w-u-(η)	τ	242.2–247.6	87.4–92.4
s-r-u	αδ	248.5–249.1	93.2–93.7	s-w-u-(η)	τ	243.9–247.9	89.7–89.8
d-r	δ	247.0, 247.3	92.9, 93.7	s-r	γε	232.3–232.9	78.2–78.7
d-r-u	δ	245.5, 245.5	91.4, 92.0	r-ξ	γε	237.5, 238.9	82.0, 83.0
s-r-u	δ	244.2, 244.5	88.3, 88.7	d-r-(ξ)	ε	248.5–252.7	95.2–100.5
d-w-ξ	α	246.2, 246.7	89.5, 89.7	d-r-u	ε	248.2, 248.3	95.3, 95.0
d-w-η	α	246.4, 249.8	89.3, 89.6	s-r	βδ	231.4–231.6	78.0–78.2
d-w-u-η	α	240.6, 244.1	84.4, 84.2	r-ξ	βδ	237.0, 237.8	82.4, 83.3
s-w-u-(η)	α	243.6, 248.8	88.8, 93.4	s-w-(η)	β	246.0–250.7	93.9–97.4
s-w-(η)	γτ	251.0, 251.5	97.5, 98.1	d-w-η/ξ	none	253.1–256.2	100.1–101.9
w-ξ	γτ	255.9, 257.1	101.4, 102.3	s-w-u-(η)	none	248.3–250.7	94.8–95.9
s-w-(η)	γ	244.1–244.7	89.4, 89.6	d-w-u-(η)	none	247.4–249.6	94.2–96.1

**Table 9:** Ranges of the relative energy decrease (kcal/mol) in solution with respect to in vacuo. DFT/B3LYP/6-31+G(d,p) results from full optimisation calculations in vacuo and from single point PCM calculations in solution. The types of conformers are characterised by the position of the first IHB and the orientations of the other phenol OH (under the PHL heading) and the types and combinations of IMHB (under the IMHB heading). When only two values are available for a given type, they are both reported, separated by a comma. When the two values of a range are identical, it indicates that at least three coinciding values were determined.

Conformer Type		Relative Energy in Vacuo (kcal/mol)	Relative Energy Lowering in Solution (kcal/mol)		
PHL	IMHB		Chloroform	Acetonitrile	Water
d-r	αδ	0.0–2.5	0.0–0.3	0.0–0.5	0.0–2.1
s-w-η	γτ	2.0, 2.1	0.5, 0.5	0.7, 0.7	1.0, 1.1
s-w	γτ	5.3, 5.3	1.0, 1.0	1.5, 1.6	3.7, 3.8
d-r-u	αδ	6.0, 6.0	0.4, 0.3	0.6, 0.5	1.5, 1.6
s-r-u	αδ	9.2–9.3	1.1–1.3	1.7–1.9	3.3–3.3
d-r	δ	11.2–13.7	2.2–2.3	2.9–3.3	5.1–7.0
s-w-η	γ	11.6, 11.9	2.5, 2.6	3.5, 3.6	5.6, 5.8
s-r	γε	12.0–12.1	3.4–3.7	4.7–5.1	7.5–7.7
s-r	βδ	12.8–13.0	3.8–4.0	5.2–5.5	8.1–8.2
d-w-ξ	α	13.8, 13.8	3.1, 3.3	4.3, 4.4	7.6, 7.6
d-w-η	α	14.0, 14.0	2.8, 2.8	4.2	7.3, 7.5
s-w	γ	14.0, 14.1	2.9	4.1, 4.0	7.7, 7.5
d-w-u-η	α	15.2, 15.3	2.0, 2.0	2.8, 2.8	4.7, 4.7
s-w-u-η	τ	15.6, 15.8	1.8, 1.8	2.5, 2.5	4.5, 4.4
s-w-u-η	α	16.4, 18.3	1.9, 1.7	2.7, 2.5	4.9, 4.9
d-r-u	δ	16.9, 16.9	2.4, 2.4	3.3, 3.4	6.3, 6.3
d-w-η/ξ	τ	17.3–19.3	3.7–4.2	5.6–5.9	9.1–9.8
s-w-η	β	17.5, 17.8	3.1, 3.1	4.3, 4.4	7.3, 7.5
d-w-u-η	τ	17.7, 19.0	2.8, 2.5	3.9, 3.6	5.9, 5.6
s-r-u	δ	17.9, 18.0	3.1, 3.0	4.5, 4.2	7.6, 7.5
d-r-ξ	ε	18.4, 19.0	3.7, 3.7	5.2, 5.3	8.1, 8.3
s-w-u	α	18.9, 20.6	2.5, 1.7	3.7, 2.8	7.3, 6.7
s-w	β	19.7, 20.2	3.2, 3.6	4.6, 5.3	8.9, 9.8
w-ξ	γτ	20.7, 20.7	4.2, 4.5	6.0, 6.2	9.8, 10.2
d-r	ε	20.7, 21.4	3.9, 3.9	5.8, 5.9	10.1, 10.4
d-w	none	22.7–24.0	3.1–4.7	4.5–6.8	8.3–12.1
d-r-u	ε	23.1, 24.0	3.5, 3.7	4.8, 5.3	8.7, 9.2
r-ξ	βδ	24.2, 25.1	4.7, 5.4	6.6, 7.4	10.3, 11.2

**Table 10:** Ranges of the free energy of solvation (ΔG_solv_) in the solvents considered DFT/B3LYP/6-31+G(d,p) results from single point PCM calculations in solution. The types of conformers are characterised by the features that prove relevant in relation to the ranges of the given dipole moment, i.e., the first IHB and the orientations of the other phenol OH (under the PHL heading) and the types and combinations of IMHB (under the IMHB heading). When only two values are available for a given type, they are both reported, separated by a comma. Ranges between negative numbers are indicated by reporting the two end-values in brackets, separated by a comma; e.g., [−4.4, −2.8] indicates a range whose lowest value is −4.4 and whose highest value is −2.8.

Conformer Type		ΔG_solv_ (kcal/mol)			Conformer Type		ΔG_solv_ (kcal/mol)		
PHL	IMHB	chlrf	actn	aq	PHL	IMHB	chlrf	actn	aq
d-r	αδ	1.5–2.0	8.0–8.2	[−4.4, −2.8]	s-w	γ	−0.2	4.9, 5.3	−9.6, −7.7
s-r-u	αδ	0.6–1.4	6.6–6.8	[−5.6, −5.0]	d-w-η	τ	−1.8, −0.6	3.0, 4.1	−11.7, −10.1
d-w-ξ	α	−1.0, −0.9	4.6, 4.6	−9.5, −9.5	d-w	τ	−1.7, −1.6	3.2, 3.2	−11.3, −10.9
d-w-η	α	−0.4, −0.2	5.0	−9.3, −9.0	s-r	γε	[−1.8, −0.9]	3.5–4.3	[−9.9, −9.4]
s-w-u-η	α	0.8, 0.8	6.5, 6.5	−6.8, −6.5	d-r-ξ	ε	−1.3, −1.2	3.6, 3.8	−10.2, −9.8
s-w-u	α	0.4, 1.1	5.8, 6.6	−8.6, −6.8	d-r	ε	−1.5, −0.1	3.3, 4.3	−12.0, −10.7
d-r	δ	−0.1, −0.1	5.5–5.6	[−8.9, −7.4]	s-r	βδ	−1.9–−1.3	3.1–3.8	[−10.4, −9.8]
s-r-u	δ	−0.9	4.4	−9.6	r-ξ	βδ	−2.6, −1.9	2.0, 2.9	−12.5, −11.5
s-w-η	γτ	1.4, 1.6	7.7, 7.8	−3.5, −3.3	s-w-η	β	−0.5, −0.4	4.7, 4.9	−9.2, −8.9
s-w	γτ	0.8, 1.1	6.9, 7.0	−6.2, −5.9	s-w	β	−0.8, −0.1	4.0, 5.1	−11.2, −9.8
w-ξ	γτ	−1.7, −1.7	3.2, 3.3	−11.5, −11.4	d-w	none	[−1.9, 2.6]	2.6–5.1	[−13.3, −9.3]

## Supplementary Materials

The supplementary materials are available online.
