# Supplementary material for: Intramolecular Hydrogen Bonding and Conformational Preferences of Arzanol—An Antioxidant Acylphloroglucinol
Source: Molecules. 2017 Aug 3;22(8):1294. doi: 10.3390/molecules22081294 (PMC6152391; doi:10.3390/molecules22081294)
Supplement: Supplementary file 1 [file molecules-22-01294-s001.pdf]

Figure S 1
Calculated conformers of arzanol.
Geometries and relative energies (kcal/mol, shown on the right under each image) from DFT/B3LYP/6-31+G(d,p) results in vacuo.
The structures are all shown with the phloroglucinol moiety oriented in the same way, to facilitate comparisons.

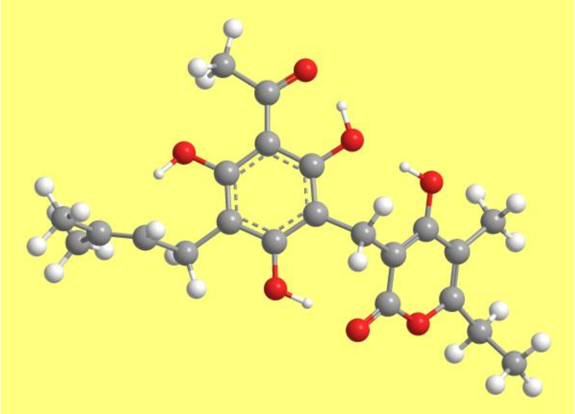
1-d-r-ξ-αδ 0.0000

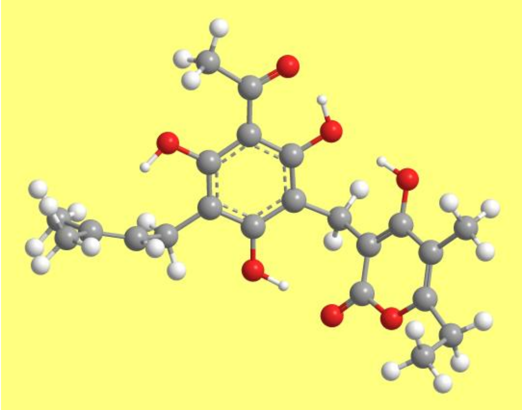
2-d-r-ξ-αδ 0.0056

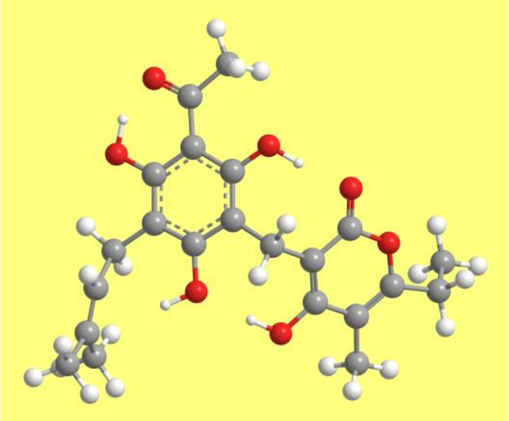
3-s-w-η-γτ 1.9751

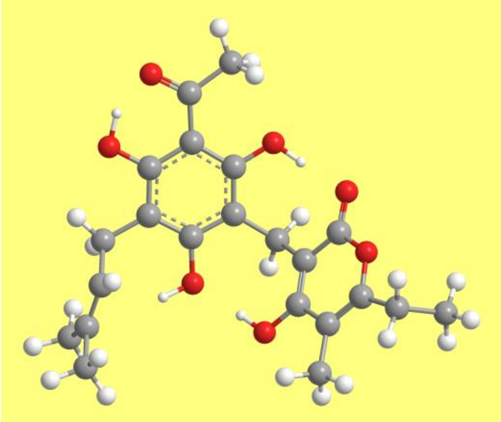
4-s-w-η-γτ 2.0946

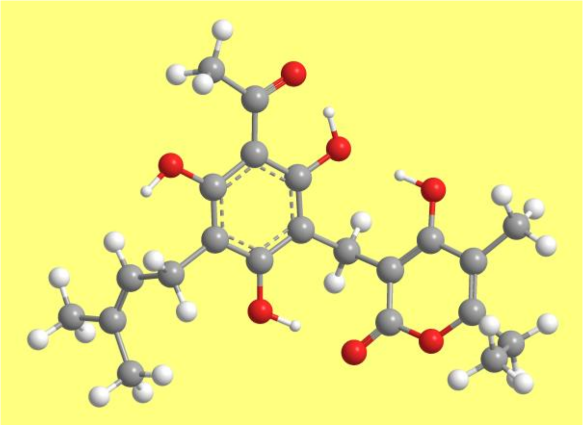

2-d-r-αδ 2.4743

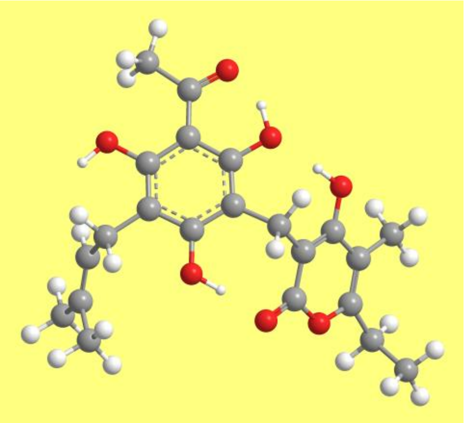

1-d-r.b-αδ 2.5019

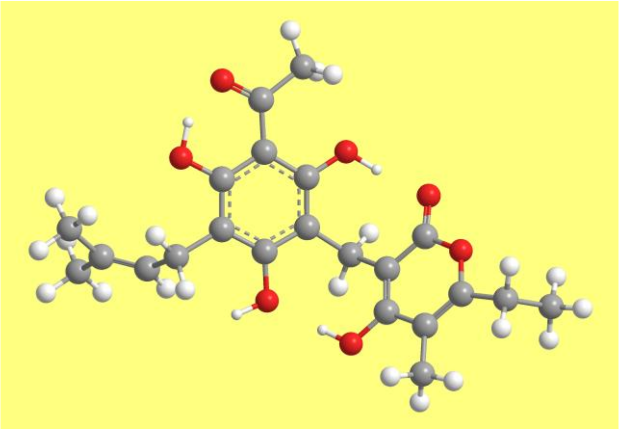

4-s-w-a-γτ 5.2593

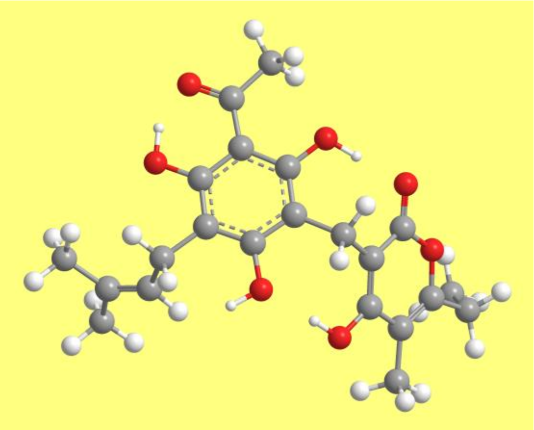

3-s-w-a-γτ 5.2980

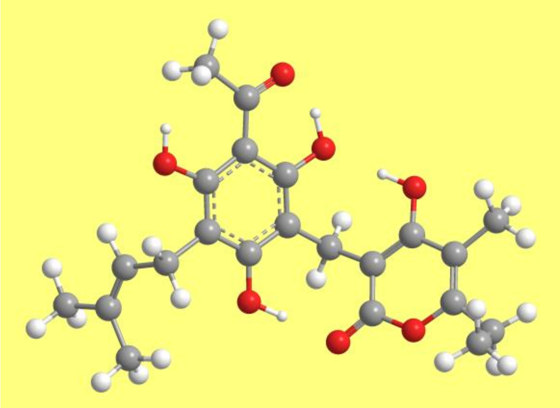

2-d-r-u-αδ 6.0121

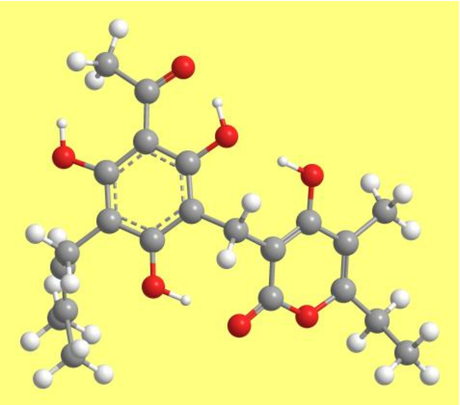

1-d-r-u-b-αδ 6.0206

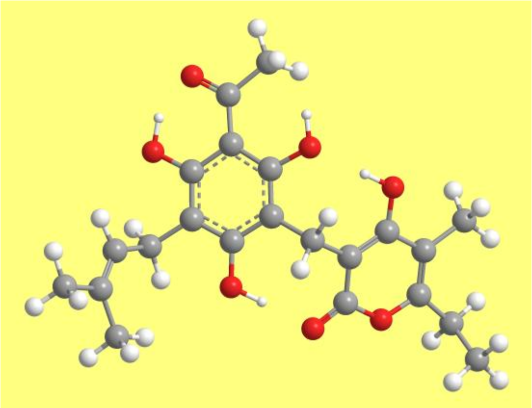

1-s-r-u-αδ 9.1833

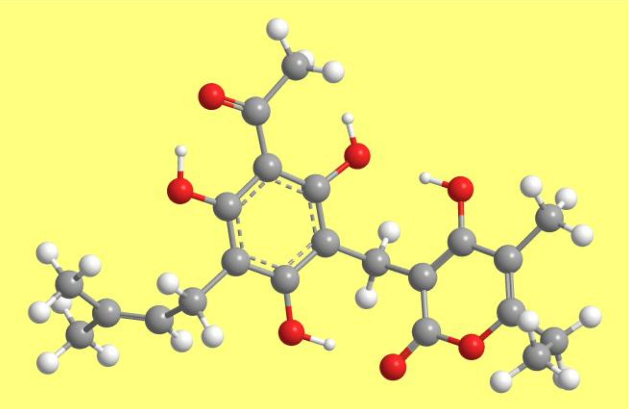

2-s-r-u-a-αδ 9.2157

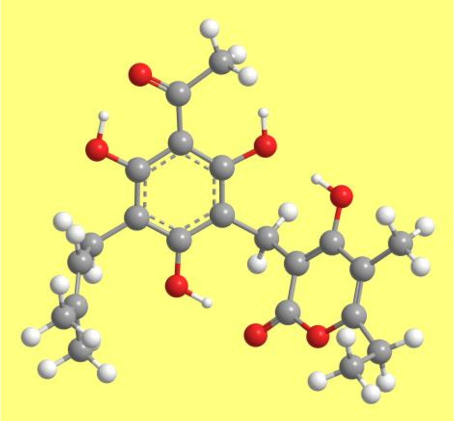

2-s-r-u-b-αδ 9.2246

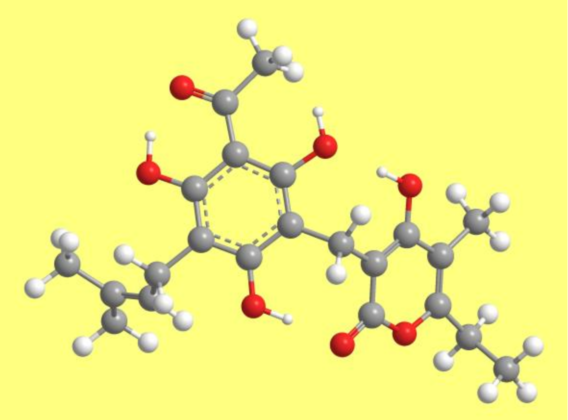

1-s-r-u-αδ 9.3065

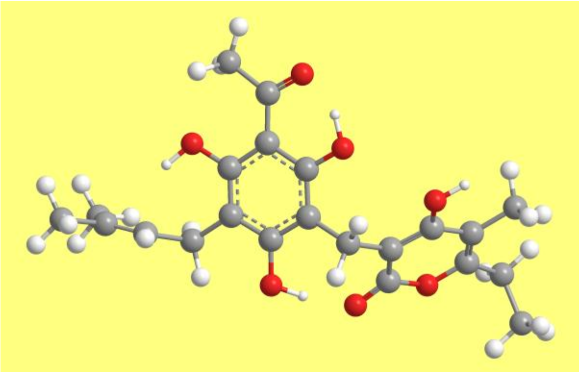

1-d-r-ξ-δ 11.1736

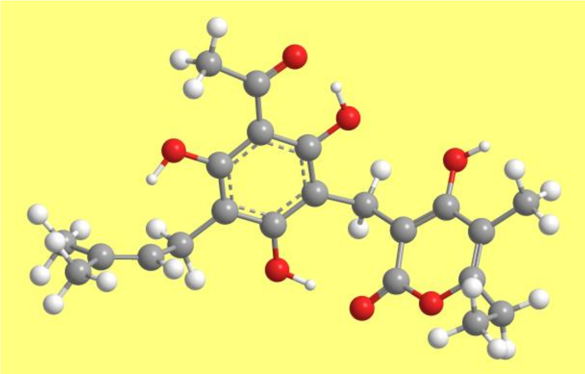

2-d-r-ξ-δ 11.2378

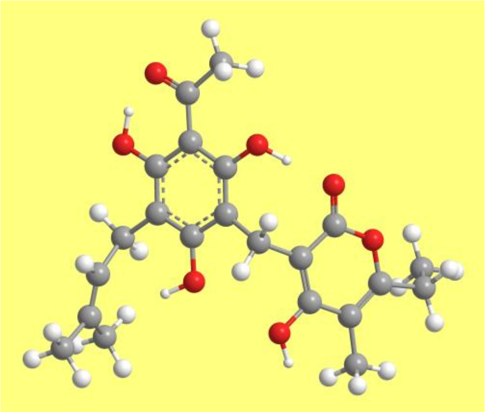

3-s-w-η-γ 11.5954

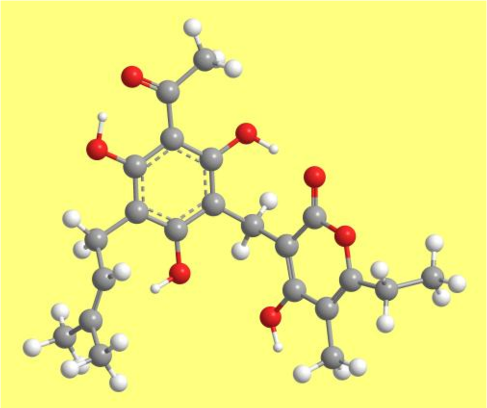

4-s-w-η-γ 11.8686

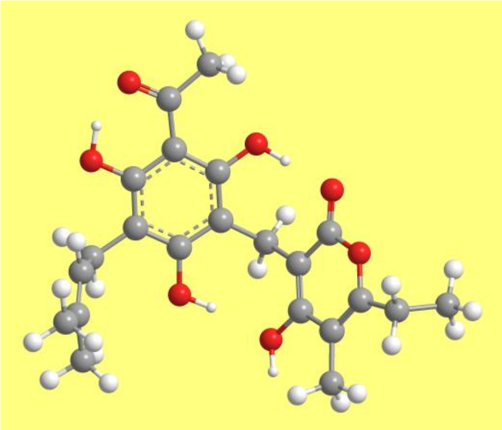

4-s-r-b-γε 11.9847

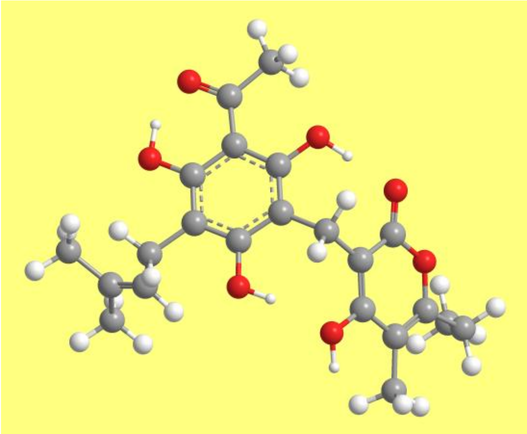

3-s-r-γε 12.0313

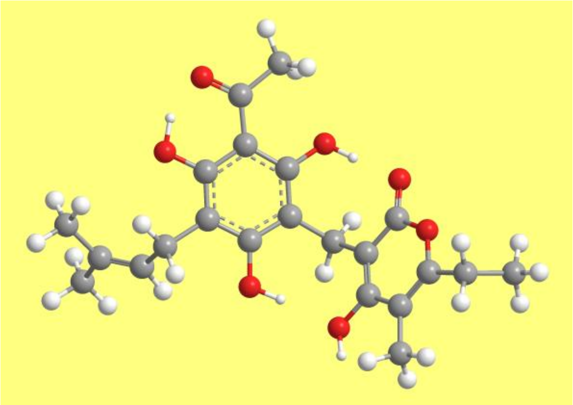

4-s-r-a-γε 12.0678

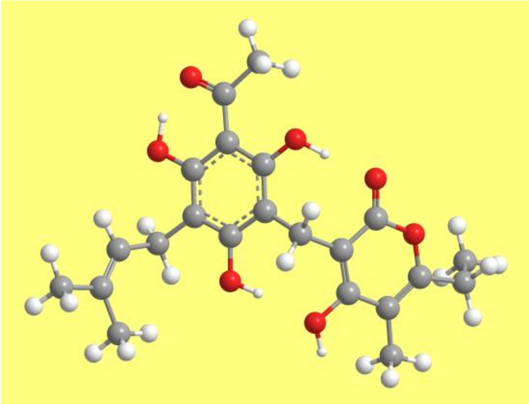

3-s-r-b-γε 12.0749

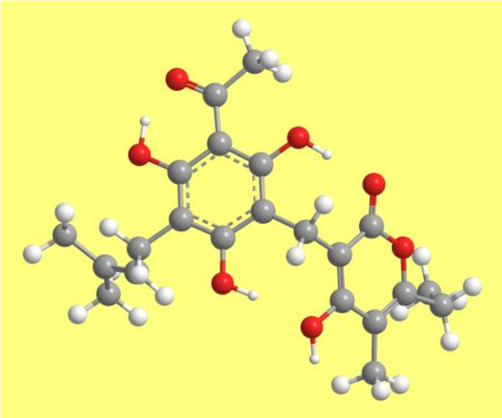

2-s-r-a-βδ 12.7916

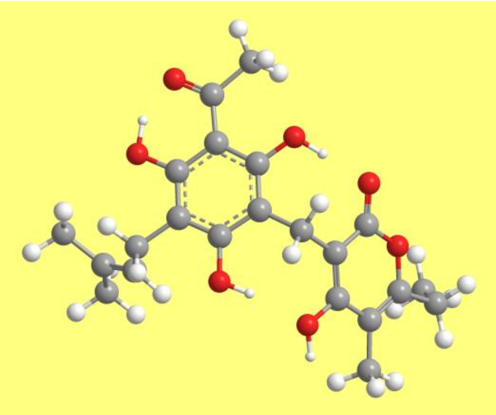

1-s-r-βδ 12.8348

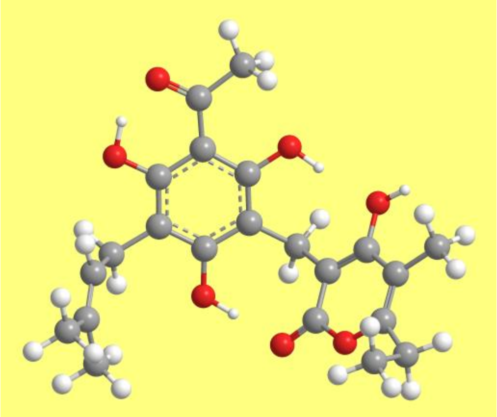

2-s-r-b-βδ 12.8659

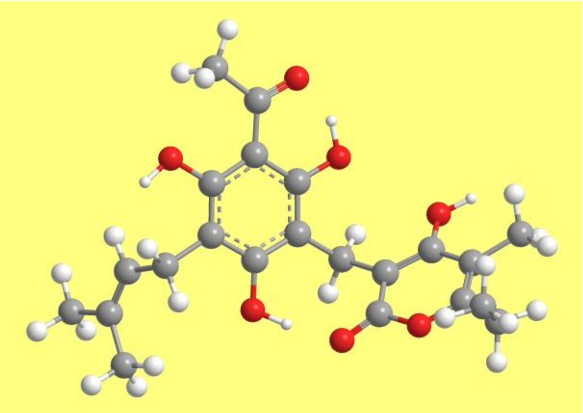

2-d-r-δ 13.6027

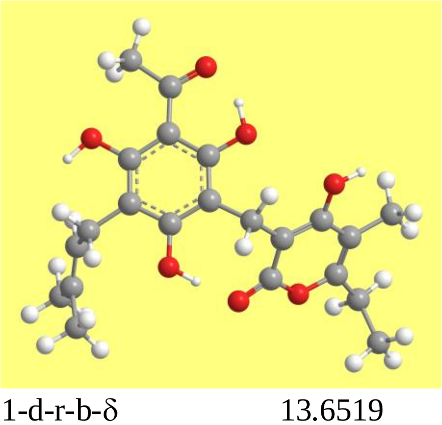

1-d-r-b-δ 13.6519

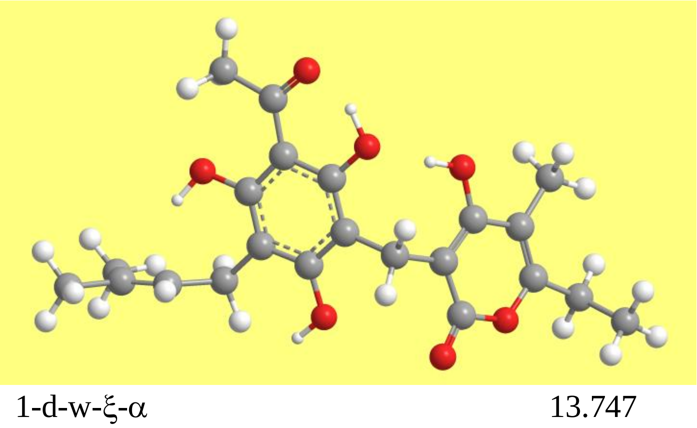

1-d-w-ξ-α 13.747

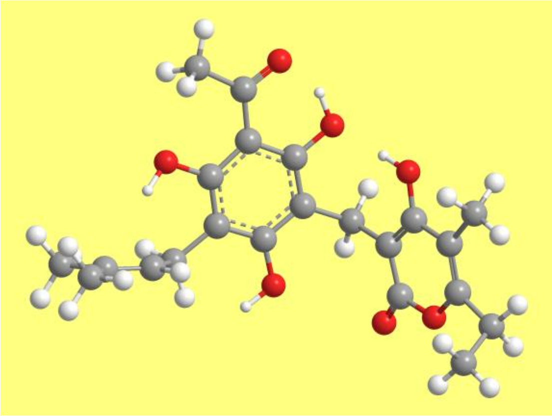

2-d-w-ξ-α 13.8252

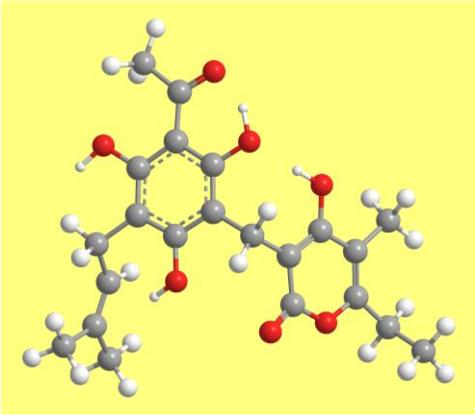

1-d-w-η-α 13.9766

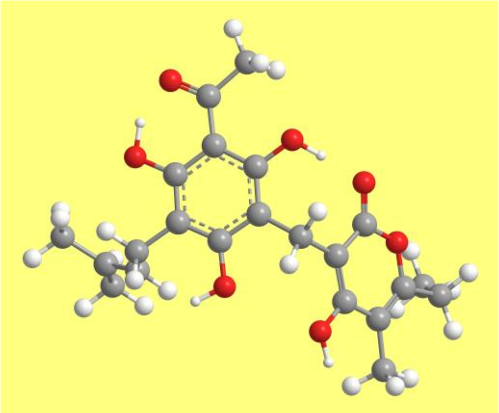

3-s-w-γ 12.5754

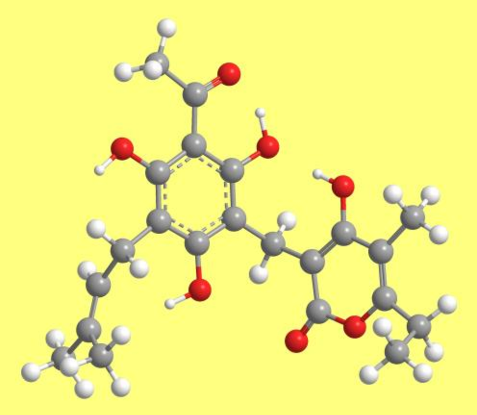

2-d-w-η-α 13.9971

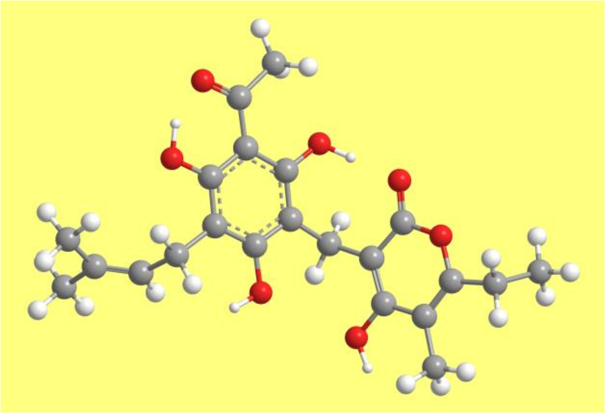

4-s-w-γ 14.0726

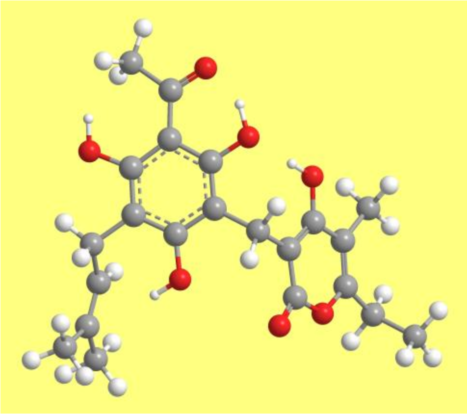

1-d-w-u-η-α 15.2303

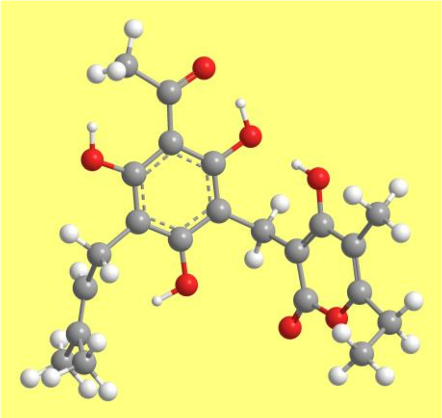
2-d-w-u-η-α 15.2641

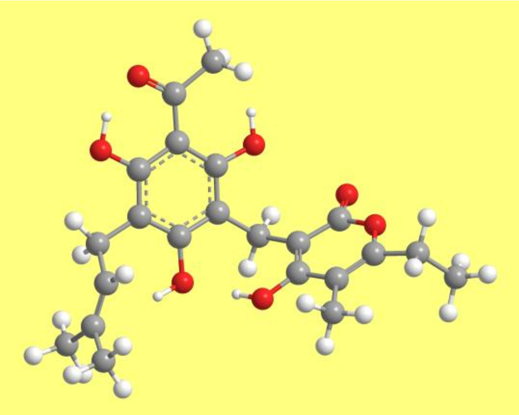
4-s-w-u-η-τ 15.6294

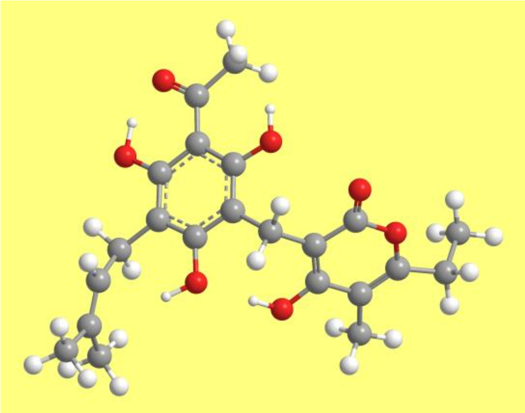
3-s-w-u-η-τ 15.7472

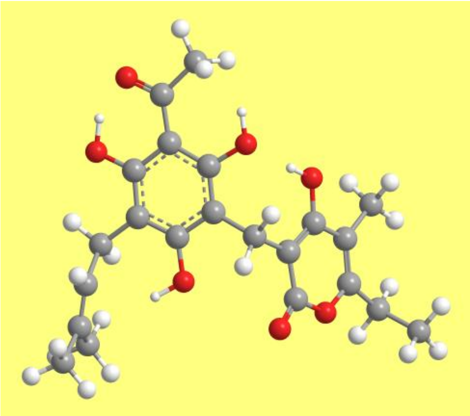
1-s-w-u-η-α 16.3870

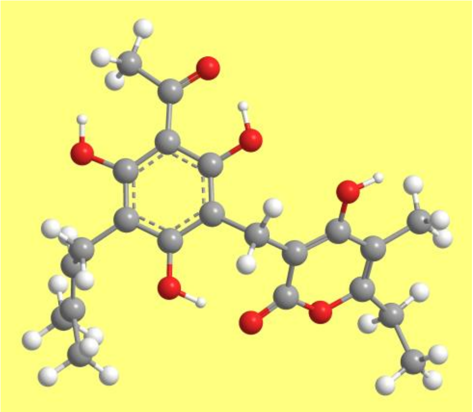
1-d-r-u-b-δ 16.8861

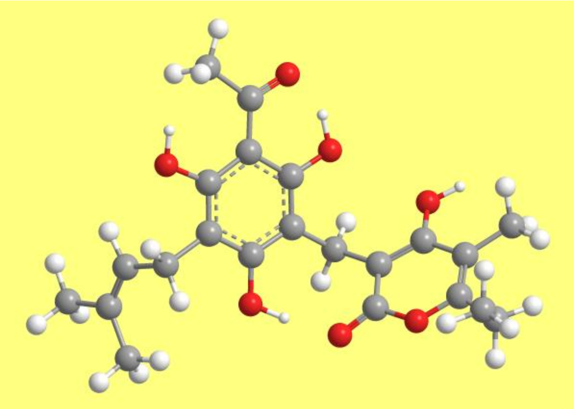
2-d-r-u-δ 17.4668

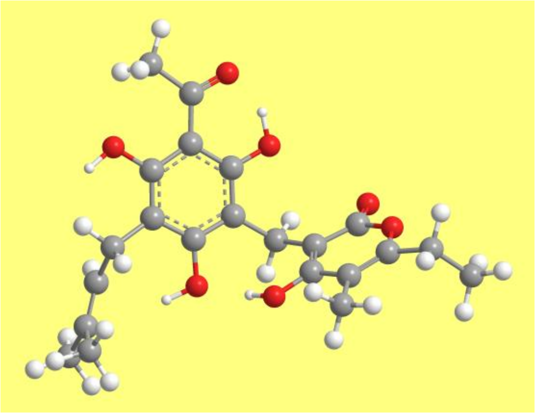

4-d-w-η-τ 17.2674

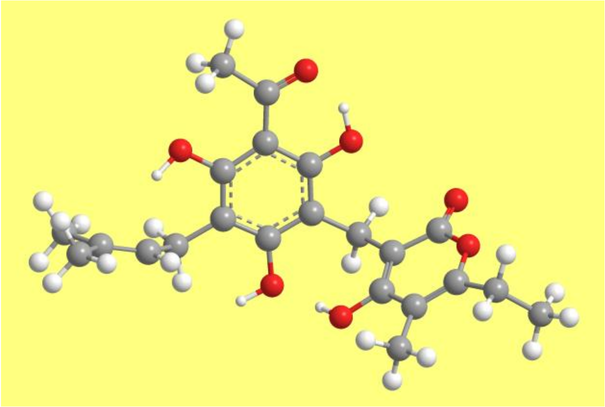

4-d-w-ξ-τ

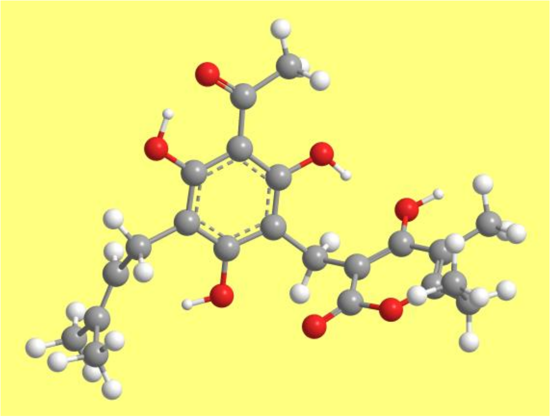

2-s-w-η-β 17.4782

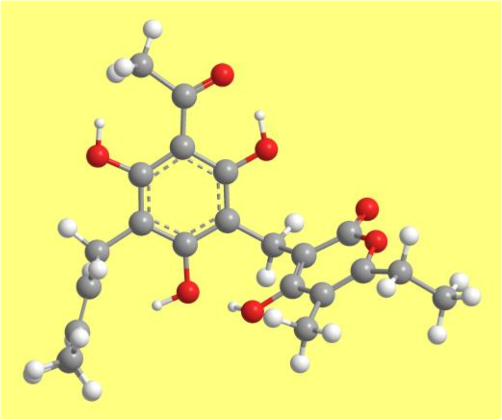

4-d-w-u-η-τ 17.6715

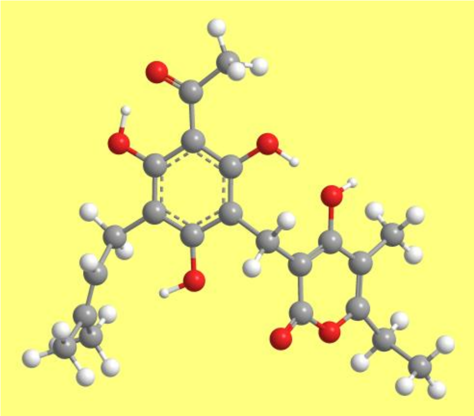

1-s-w-η-β 17.7503

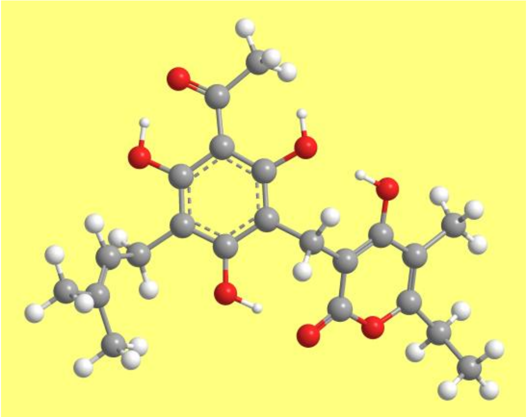

1-s-r-u-δ 17.8873

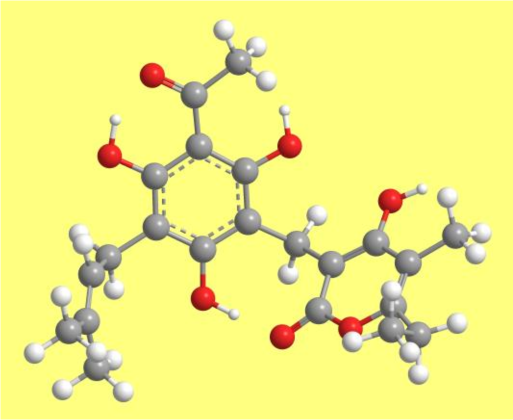

2-s-r-u-b-δ 18.0168

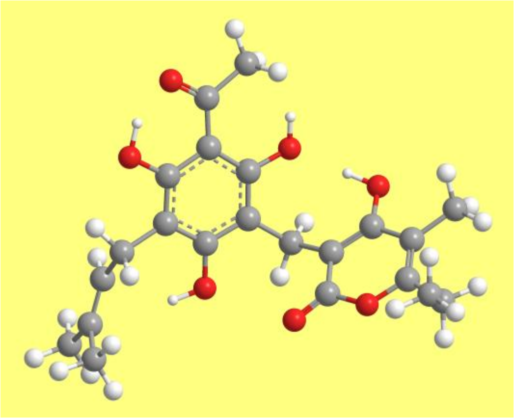

2-s-w-u-η-α 18.3159

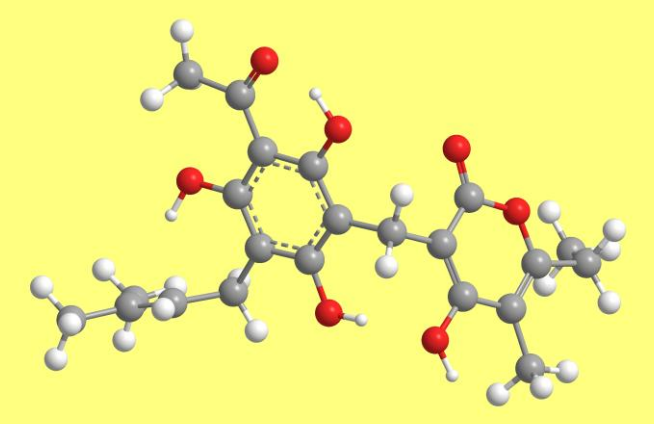

3-d-r-ξ-ε 18.3595

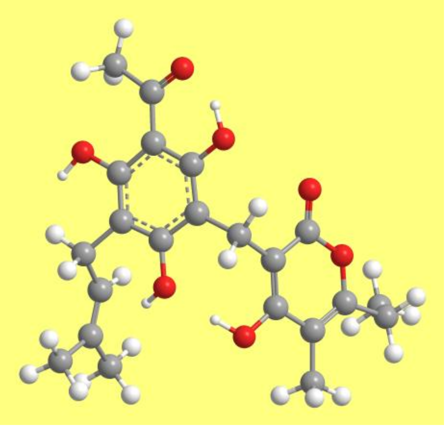

3-d-w-η-τ 18.6644

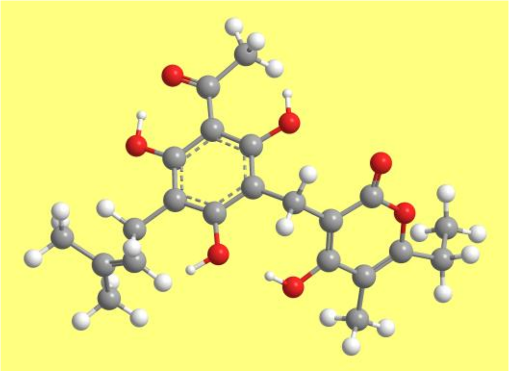

3-s-w-u-a-τ 18.8511

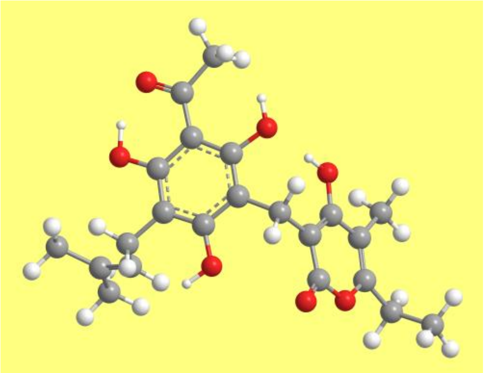

1-s-w-u-a-α 18.9152

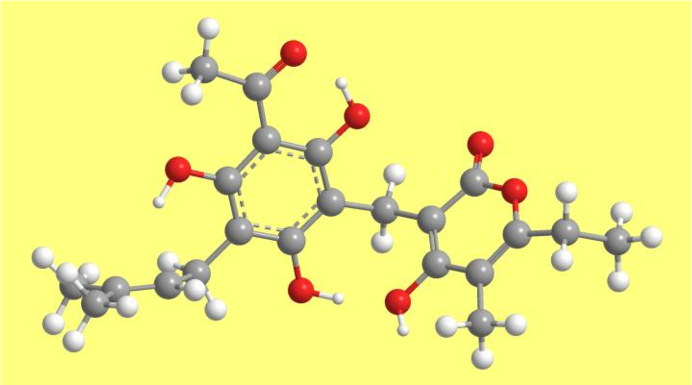

4-d-r-ξ-ε 18.9490

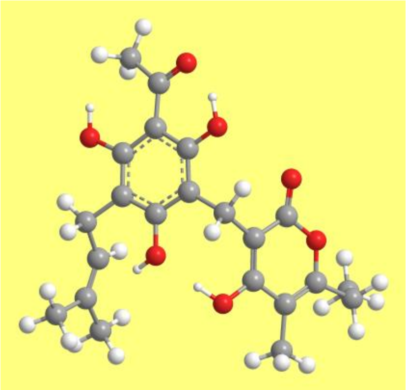

3-d-w-u-η-τ 18.9549

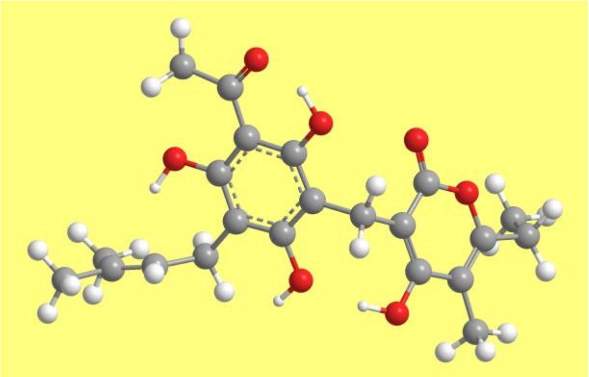

3-d-w-ξ-τ 19.2834

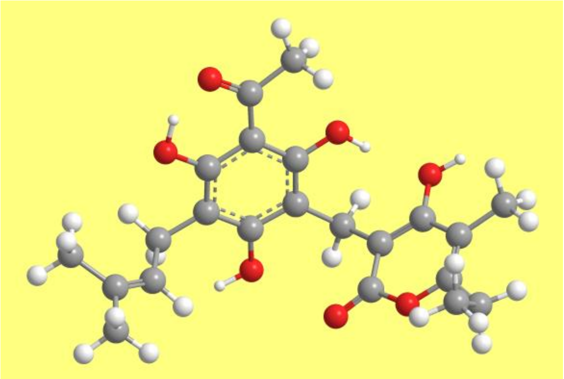

2-s-w-a-β 19.7416

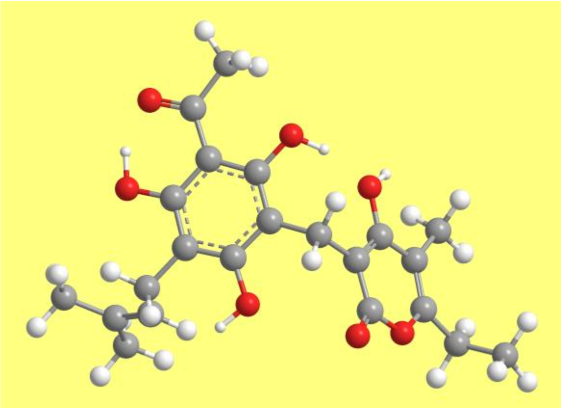

1-s-w-β 20.2229

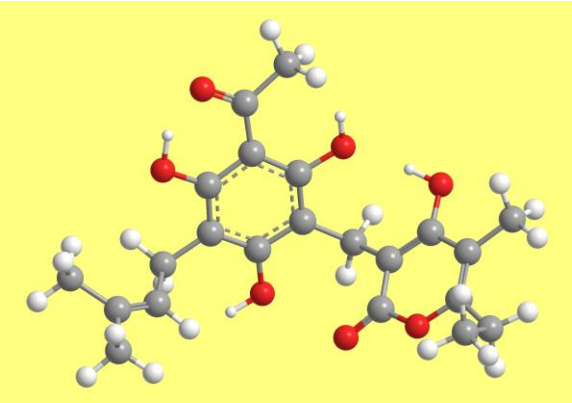

2-s-w-u-a-α 20.5679

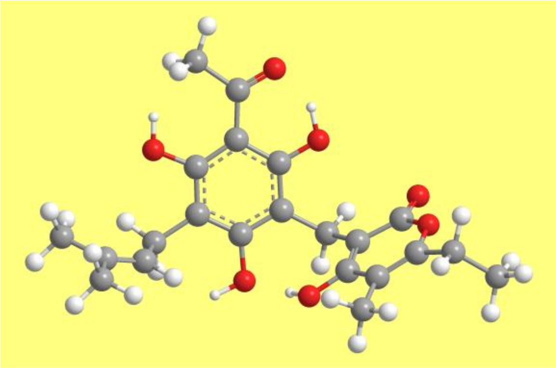

4-d-w-u-τ 20.5947

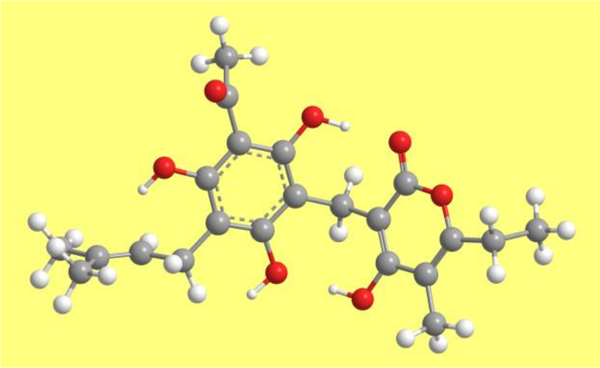

4-w-ξ-γτ 20.6825

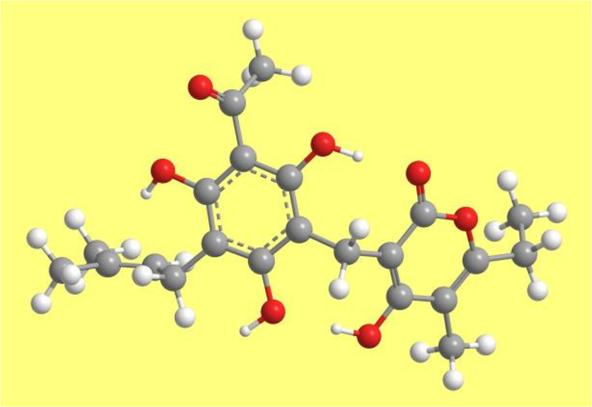

3-w-ξ-γτ 20.7340

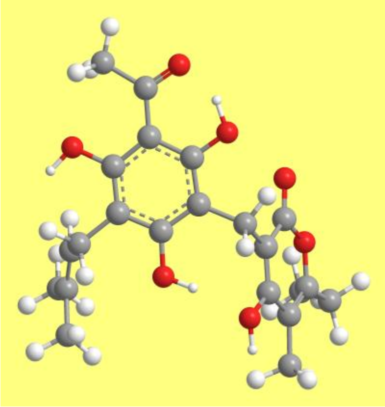

3-d-r-b-ε 20.7350

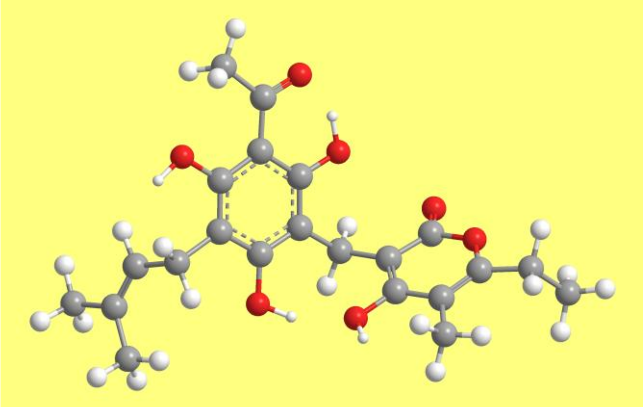

4-d-r-a-ε 21.3578

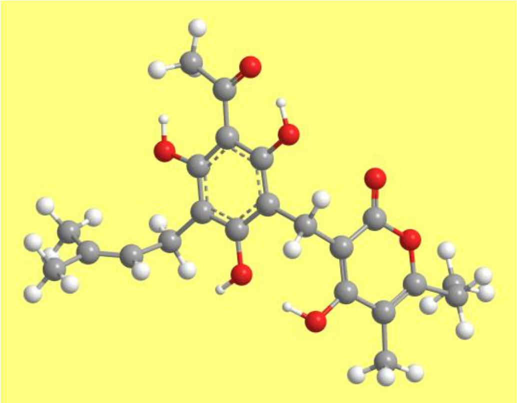

3-d-w-u-a-τ 22.0232

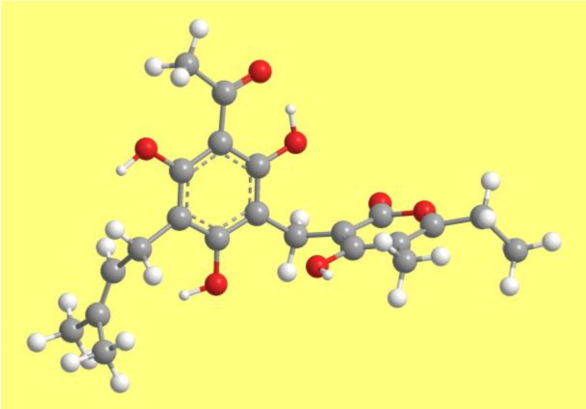

2-d-w-η 22.2956

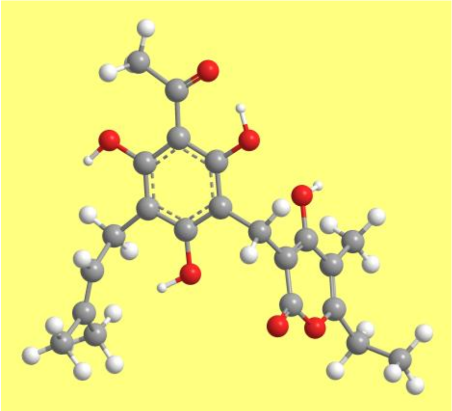

1-d-w-η 22.7227

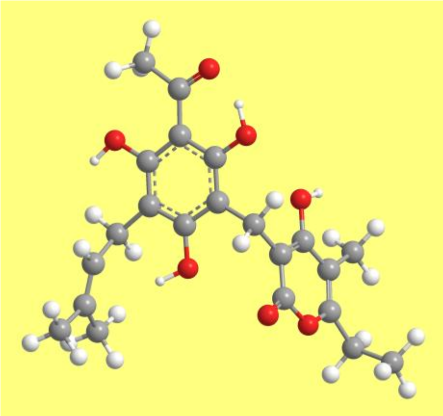

1-d-w-u-η 22.7227

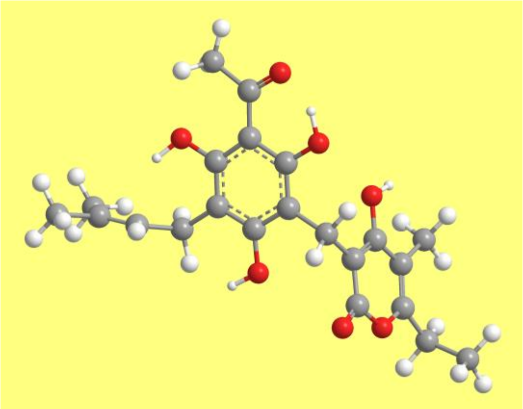

1-d-w-ξ 22.7404

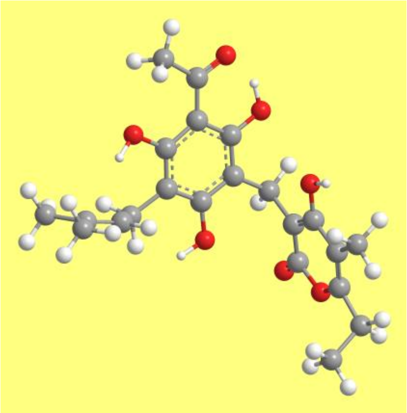

2-d-w-ξ 22.8871

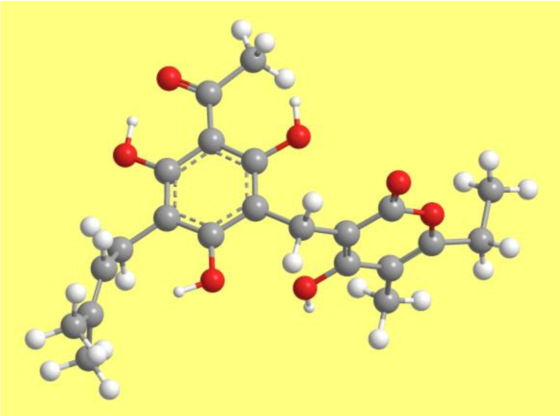

3-s-w-u-η 22.9203

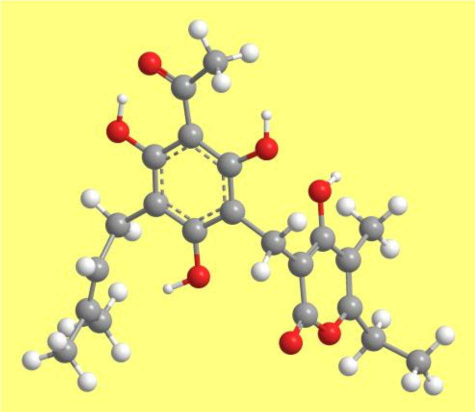

1-s-w-u-η 23.0548

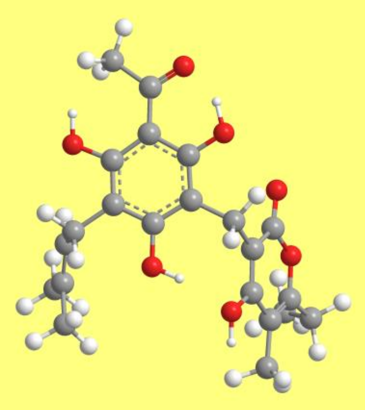

3-d-r-u-b-ε 23.1199

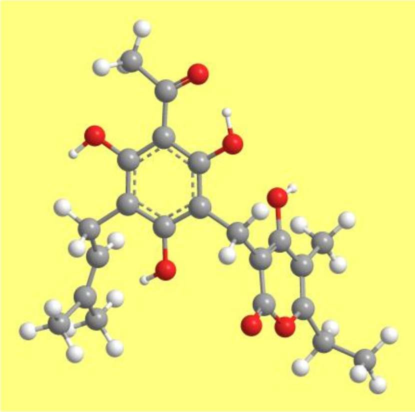

1-d-w-η’ 23.1482

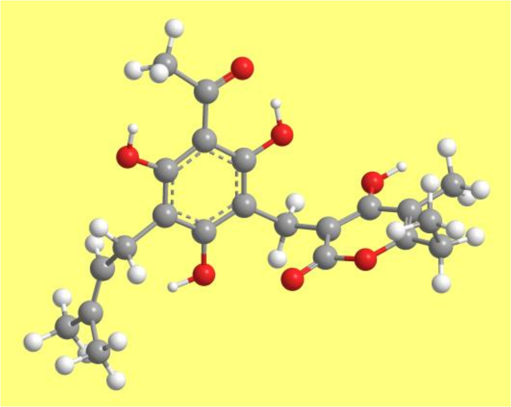

2-d-w-u-η 23.2891

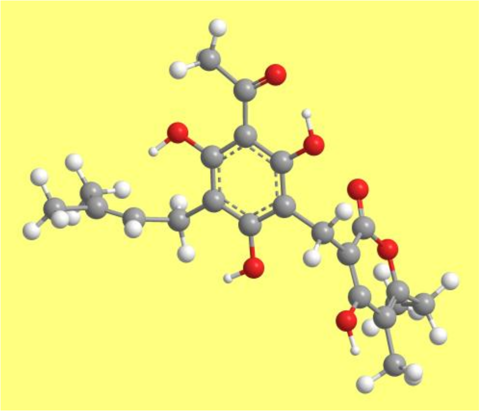

3-d-w-ξ 23.8842

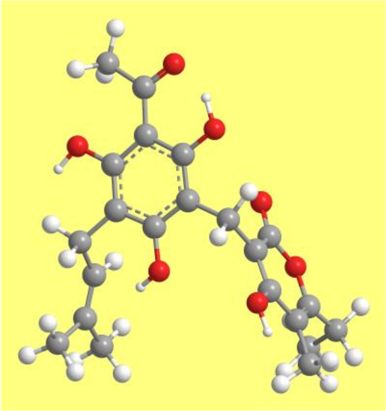

3-d-w-η 24.0045

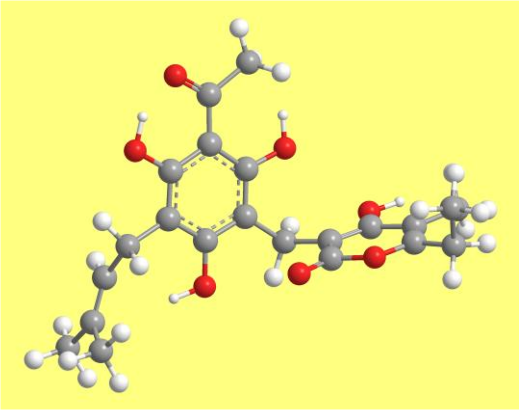

2-s-w-u-η 23.3787

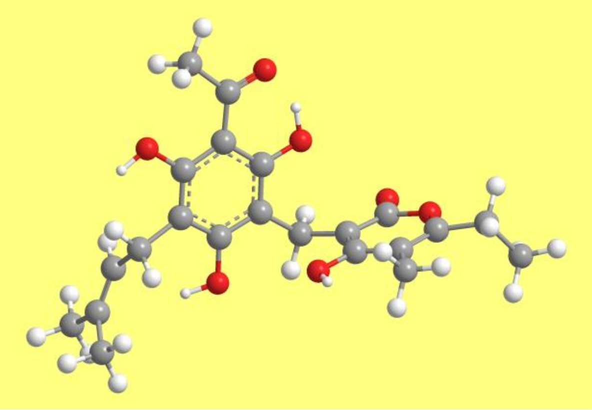

4-d-w-η 23.5982

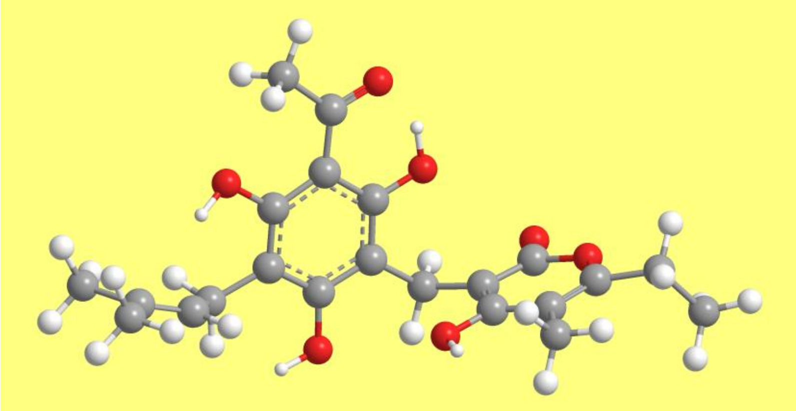

4-d-w-ξ 23.6187

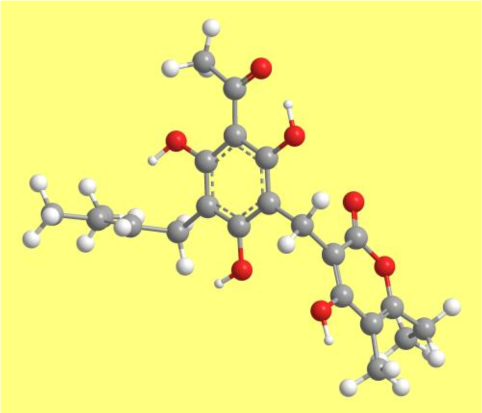

3-d-w-ξ 23.8842

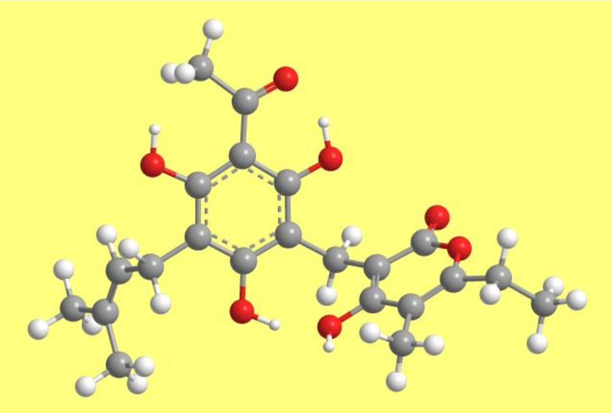

4-d-r-u-ε 23.9884

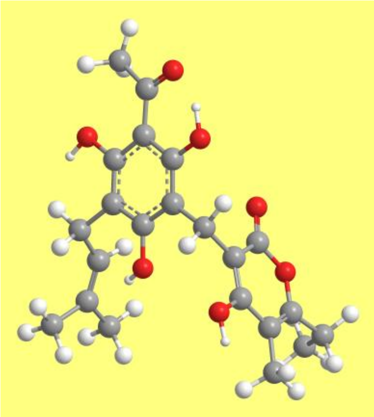

3-d-w-η 24.0045

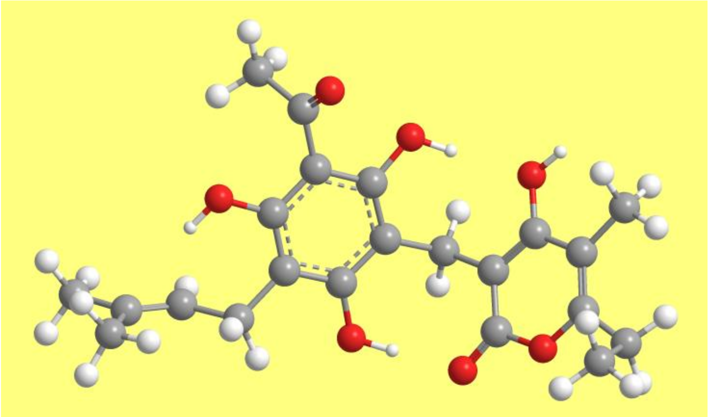

2-r-ξ-βδ 24.2020

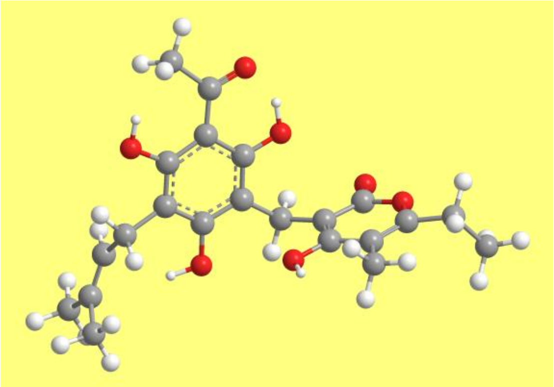

4-d-w-u-η 24.2847

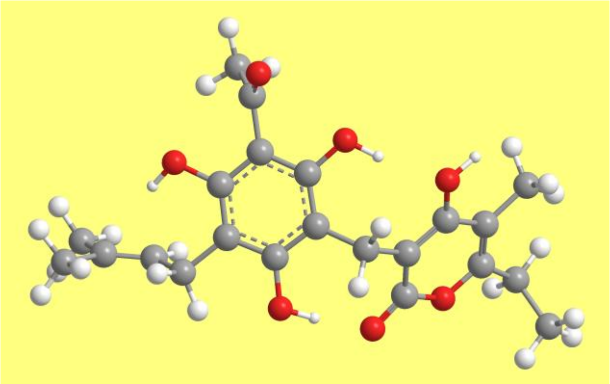

1-r-ξ-βδ 25.0941

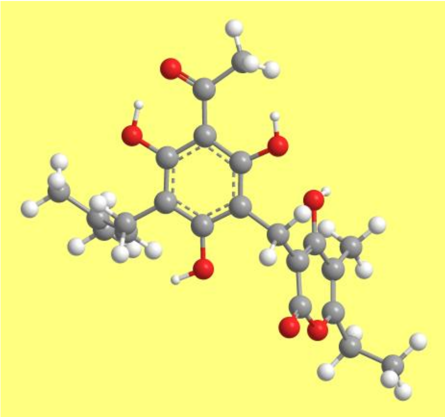

1-s-w-u 25.6076

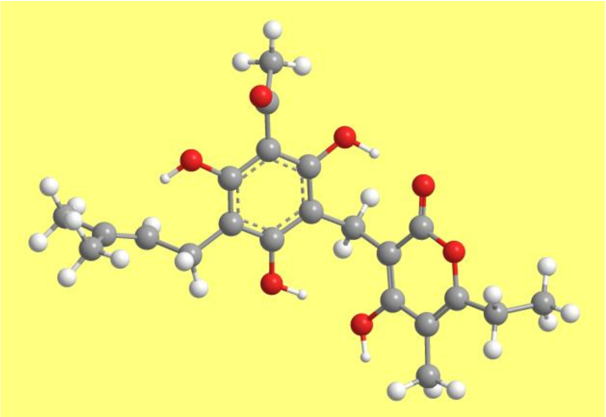

4-r-ξ-γε 25.5121

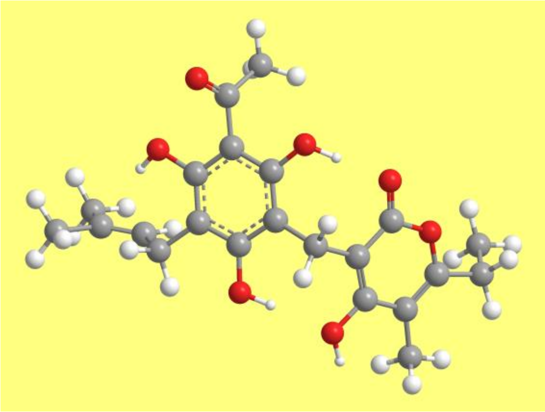

3-r-ξ-γε 25.5930

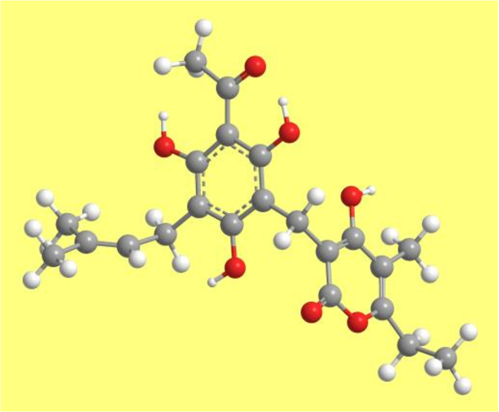

1-d-w-u 26.1093

2-d-r-αδ	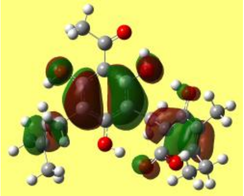	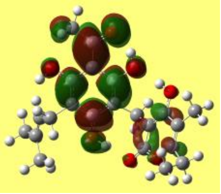	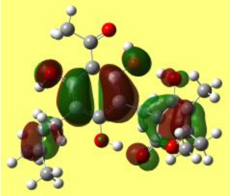	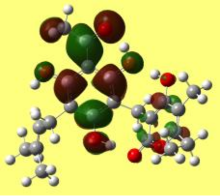
1-d-r-b-αδ	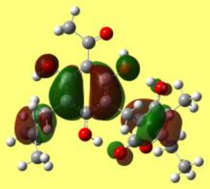	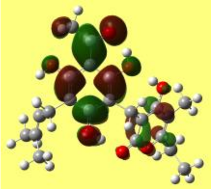	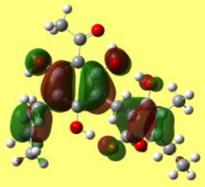	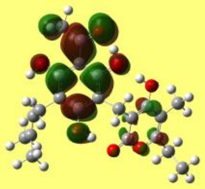
4-s-w-a-γτ	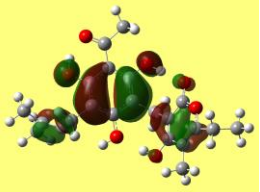	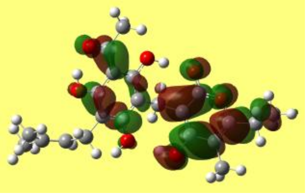	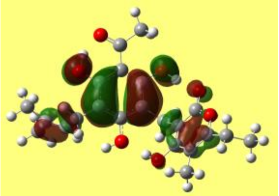	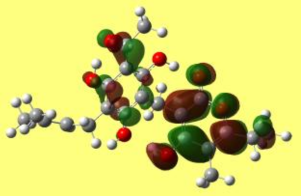
3-s-w-a-γτ	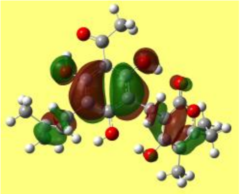	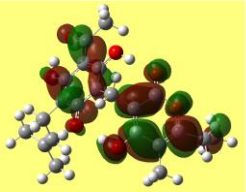	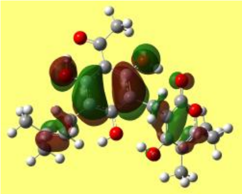	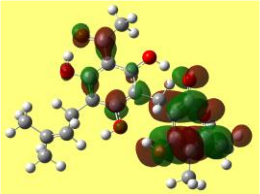
1-s-r-u-αδ	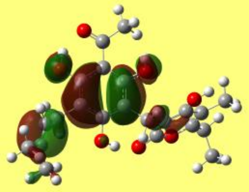	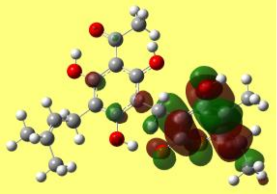	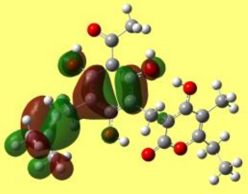	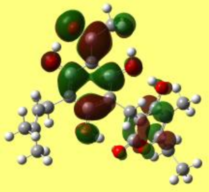
2-s-r-u-a-αδ	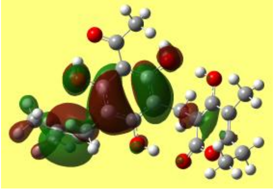	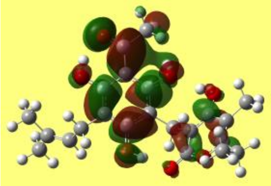	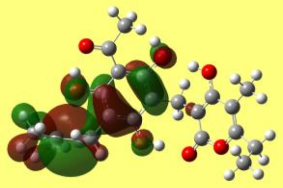	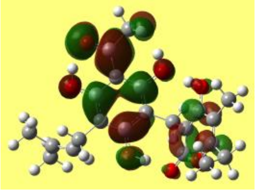

2-s-r-u-b-αδ	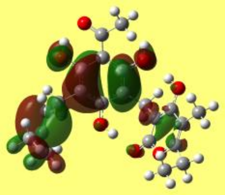	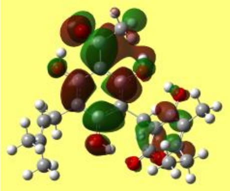	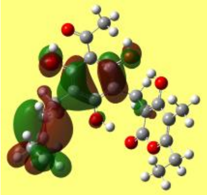	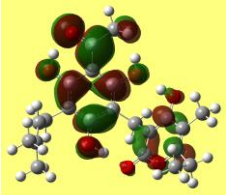
1-s-r-u-a-αδ	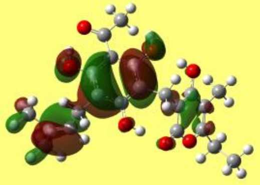	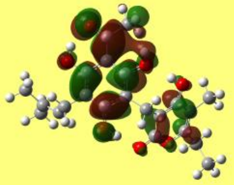	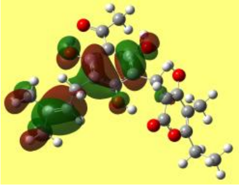	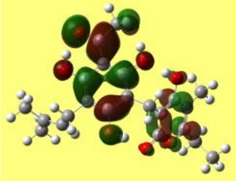
2-d-r-ξ-δ	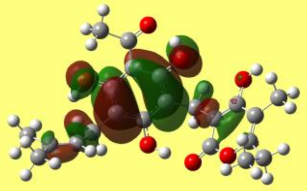	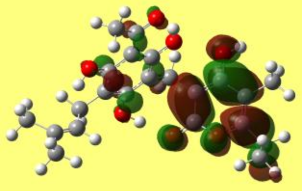	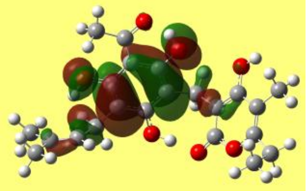	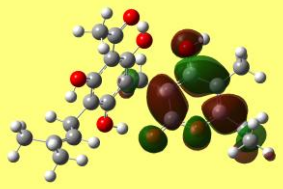
3-s-w-η-γ	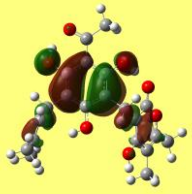	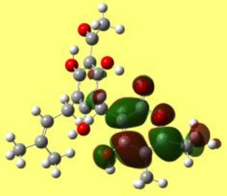	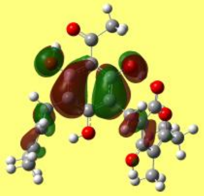	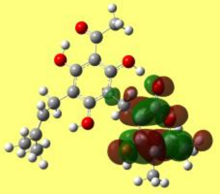
4-s-r-b-γε	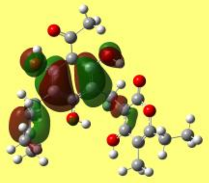	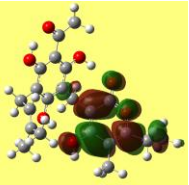	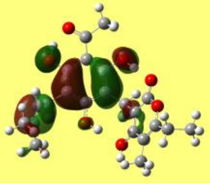	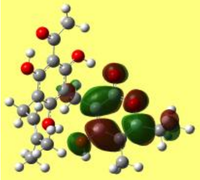
3-s-r-γε	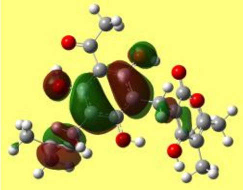	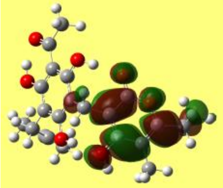	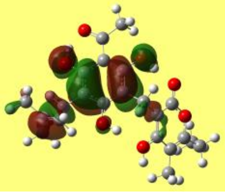	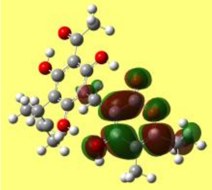

4-s-r-a-γε	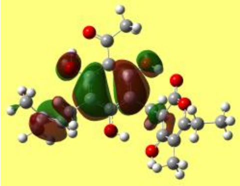	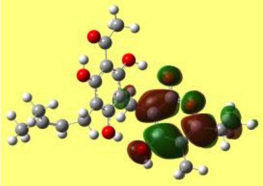	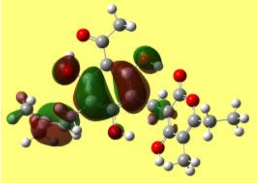	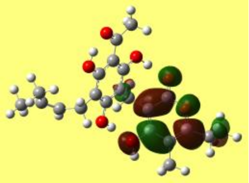
3-s-r-b-γε	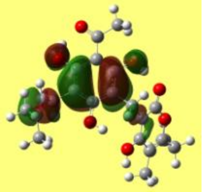	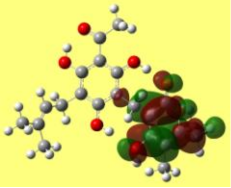	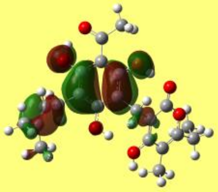	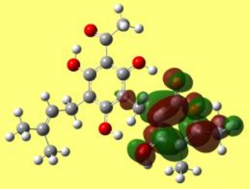
2-s-r-a-βδ	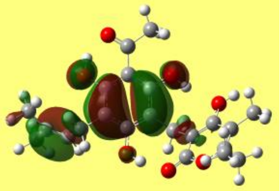	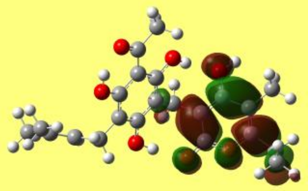	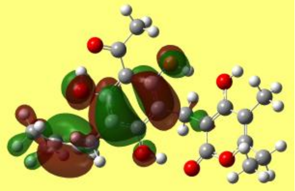	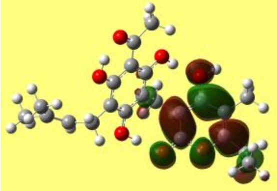
1-s-r-βδ	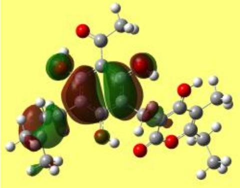	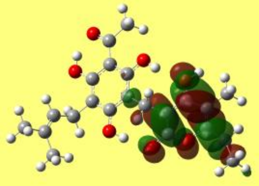	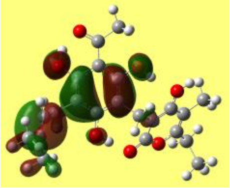	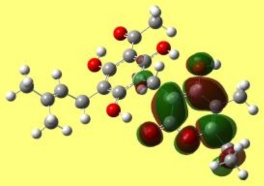
2-s-r-b-βδ	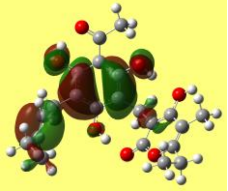	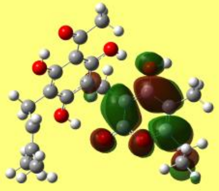	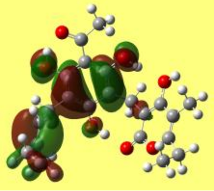	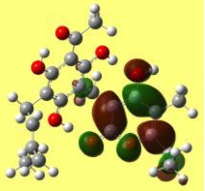
1-s-r-βδ’	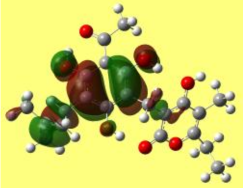	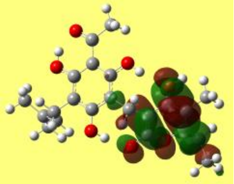	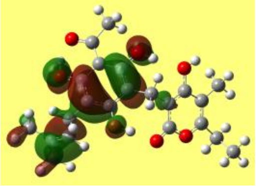	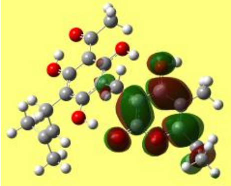

2-d-r-δ	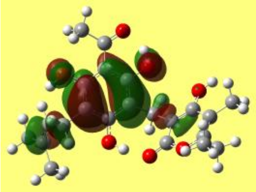	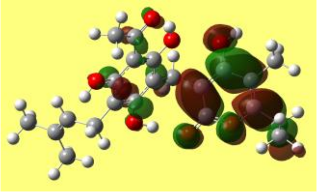	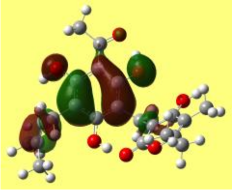	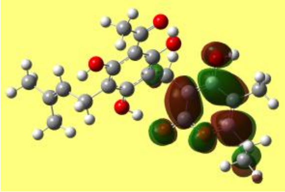
1-d-w-ξ-α	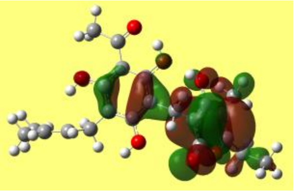	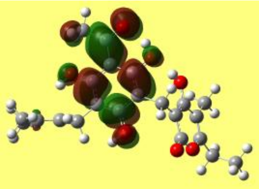	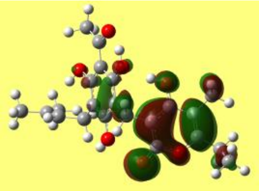	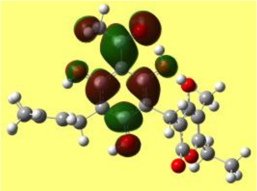
2-d-w-ξ-α	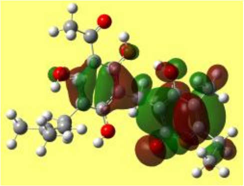	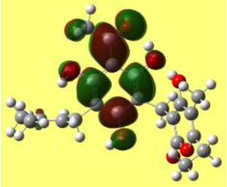	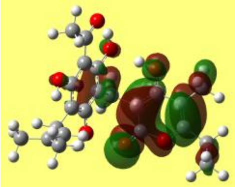	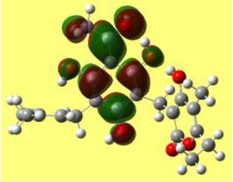
1-d-w-η-α	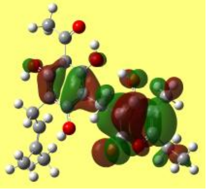	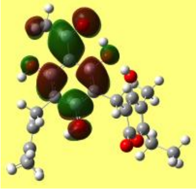	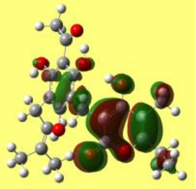	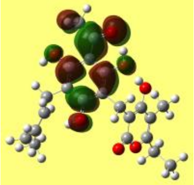
3-s-w-γ	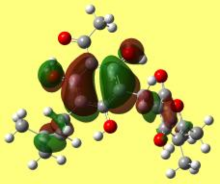	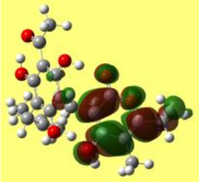	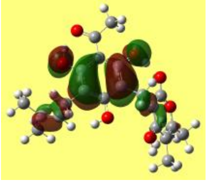	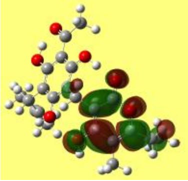
2-d-w-η-α	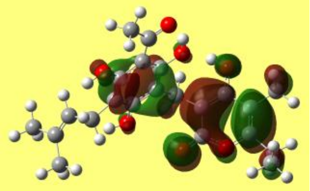	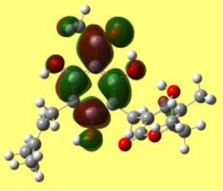	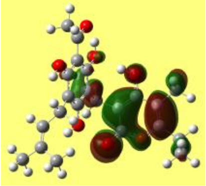	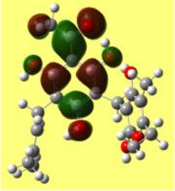

1-s-w-u-η-α	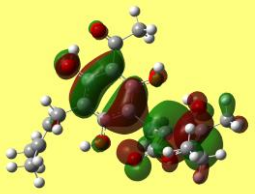	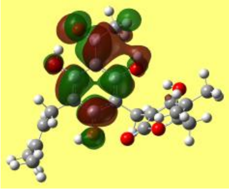	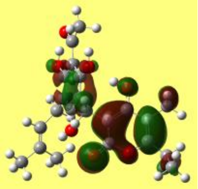	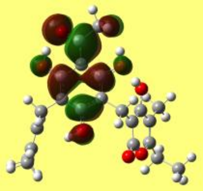
4-d-w-η-τ	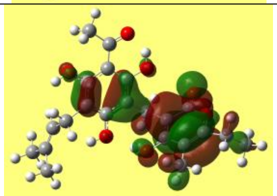	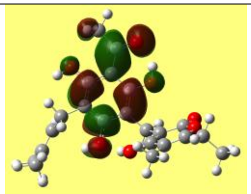	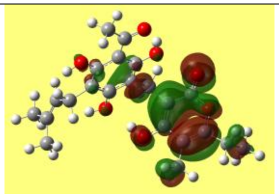	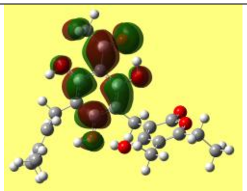
4-d-w-ξ-τ	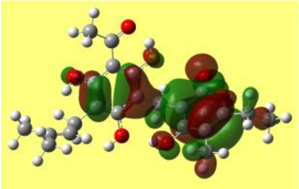	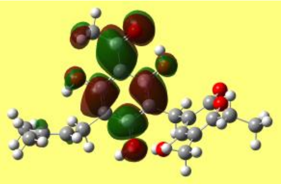	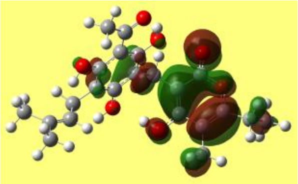	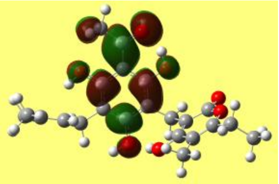
2-s-w-η-β	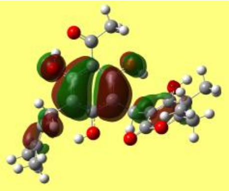	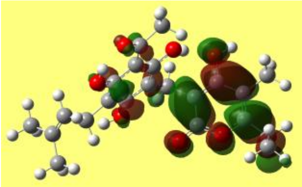	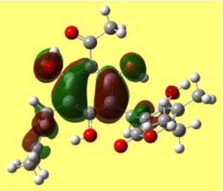	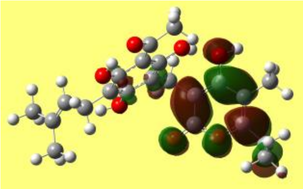
1-s-w-η-β	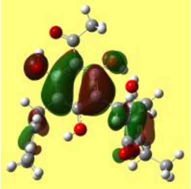	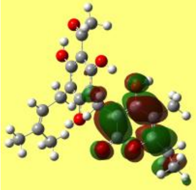	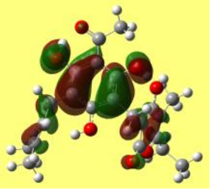	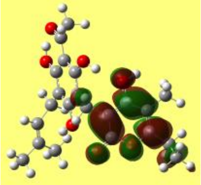
1-s-r-u-δ	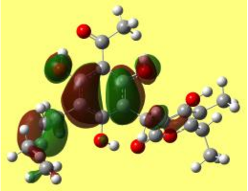	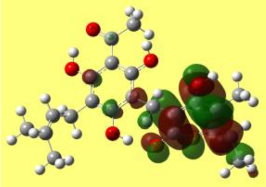	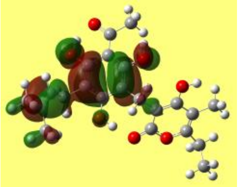	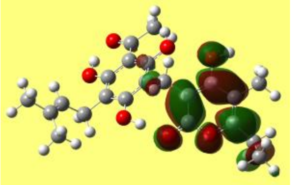

2-s-w-u-η-α	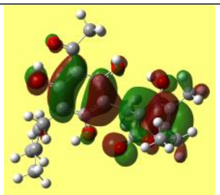	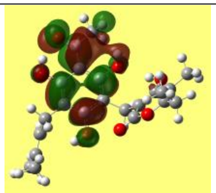	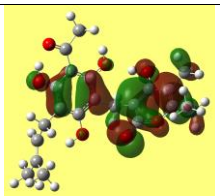	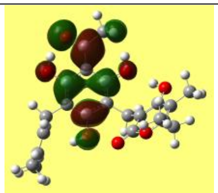
3-d-r-ξ-ε	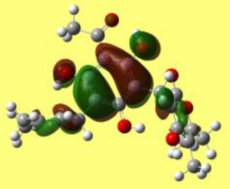	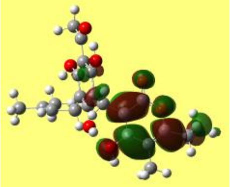	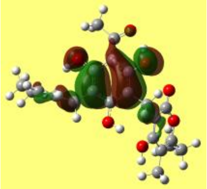	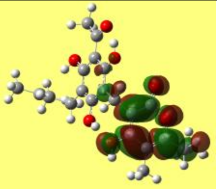
3-d-w-η-τ	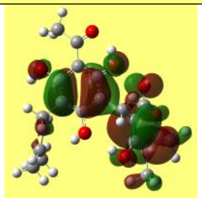	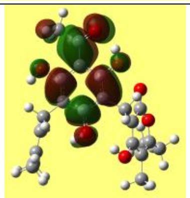	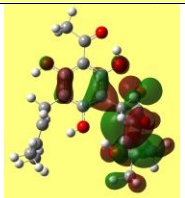	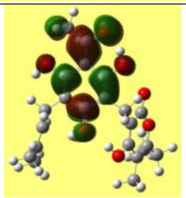
1-s-w-u-a-α	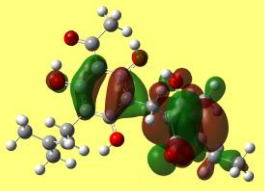	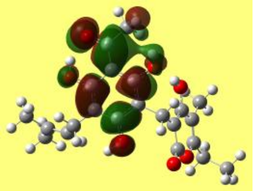	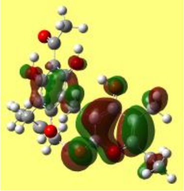	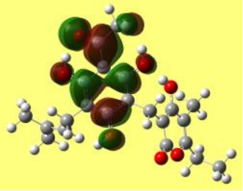
4-d-r-ξ-ε	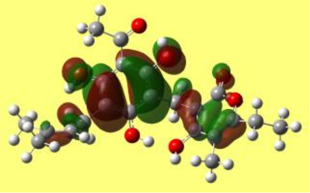	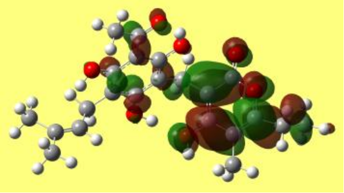	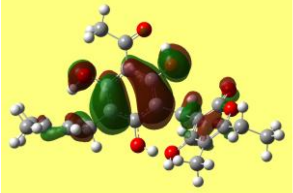	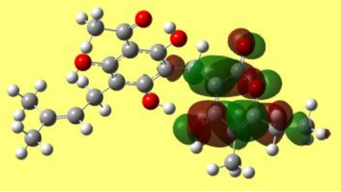

3-d-w-ξ-τ	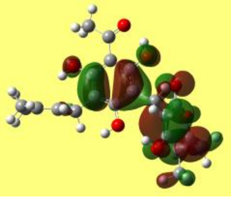	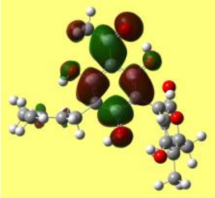	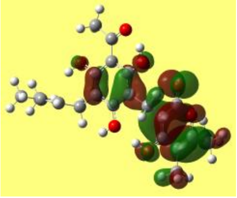	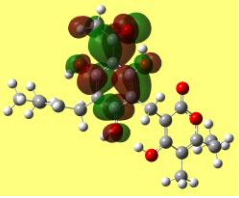
2-s-w-a-β	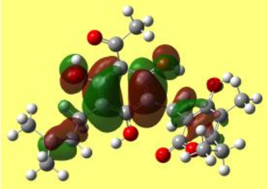	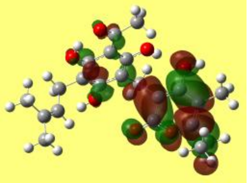	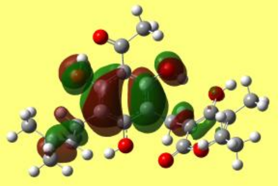	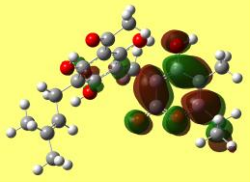
1-s-w-β	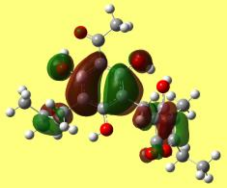	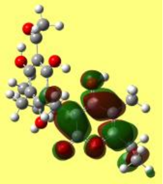	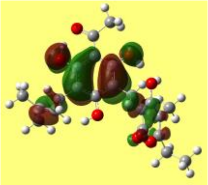	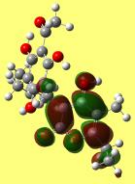
2-s-w-u-a-α	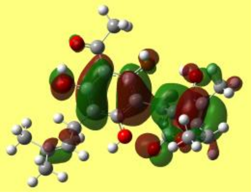	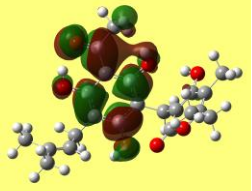	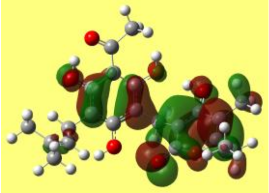	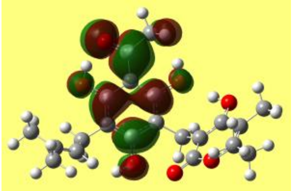
4-w-ξ-yτ	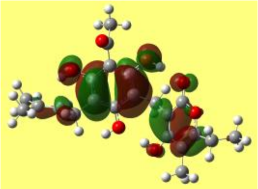	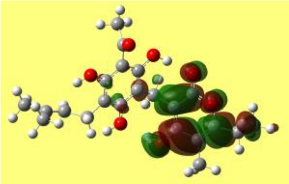	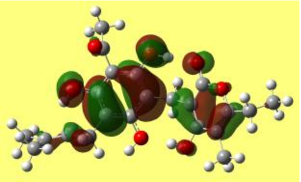	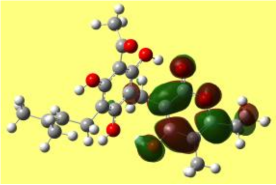
3-w-ξ-γτ	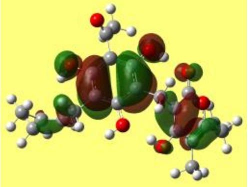	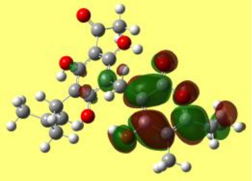	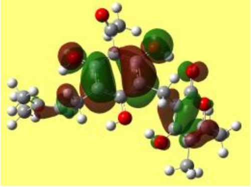	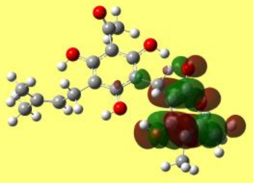

3-d-r-b-ε	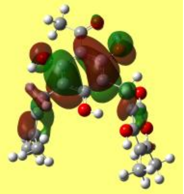	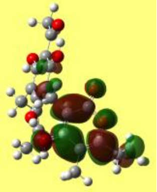	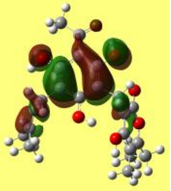	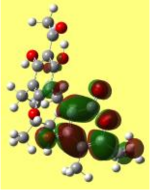
4-d-r-ε	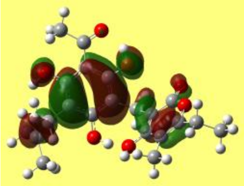	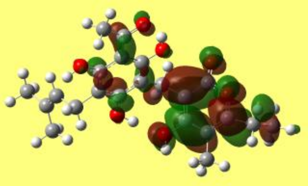	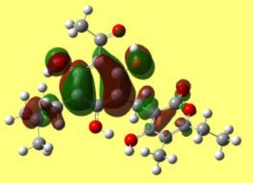	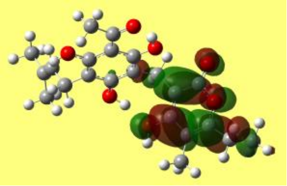
1-d-w-η	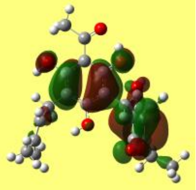	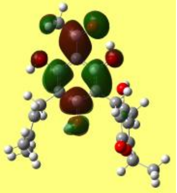	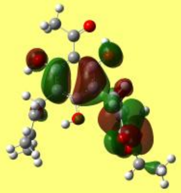	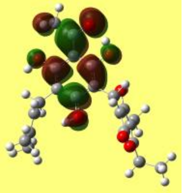
1-d-w-ξ	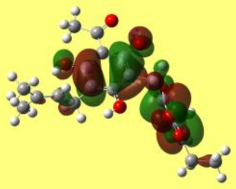	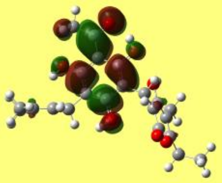	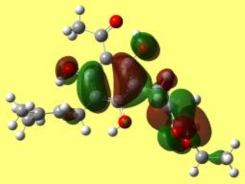	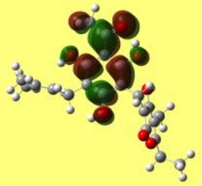
1-s-w-u-η	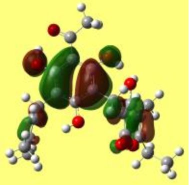	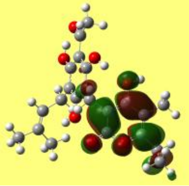	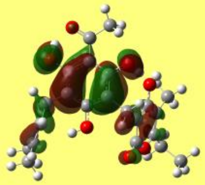	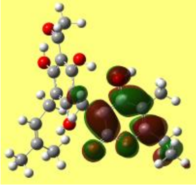
1-d-w-η’	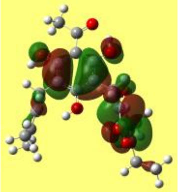	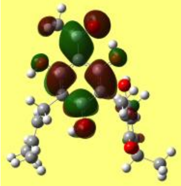	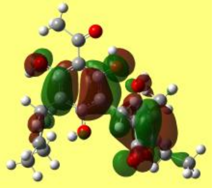	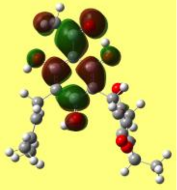

3-d-w-ξ	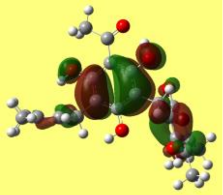	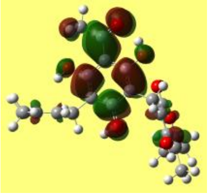	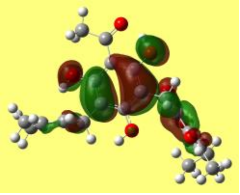	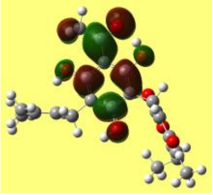
3-d-w-η	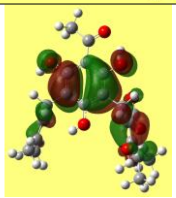	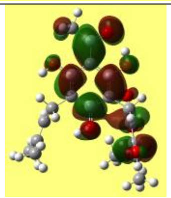	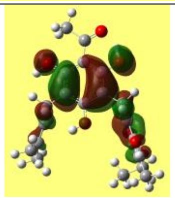	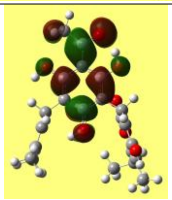
2-r-ξ-βδ	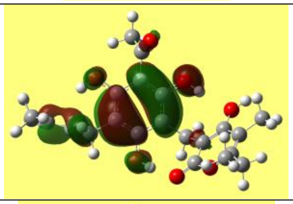	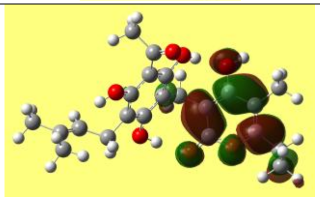	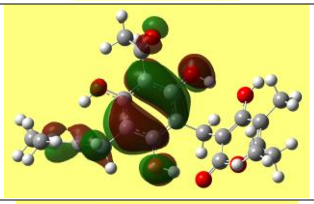	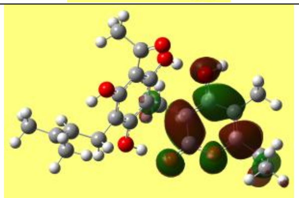
1-r-ξ-βδ	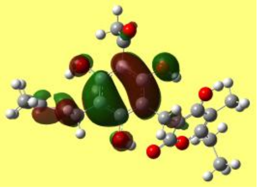	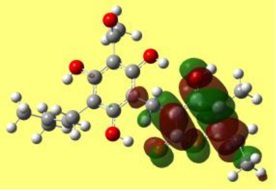	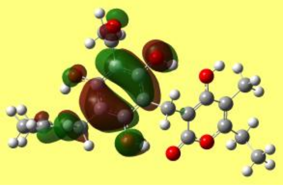	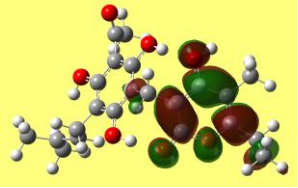

1-d-w-u-η-α	15.4795	15.1379	15.2303
2-d-w-u-η-α	16.3892	16.7274	15.2641
4-s-w-u-η-τ	15.9070	15.9382	15.6294
3-s-w-u-η-τ	15.2688	15.2406	15.7472
1-s-w-u-η-α	16.5636	16.1813	16.3870
1-d-r-u-b-δ	16.6171	17.1811	16.8861
2-d-r-u-δ	16.5753	17.2488	16.8873
4-d-w-η-τ	15.6105	16.6234	17.2674
4-d-w-ξ-τ	15.8319	16.5531	17.4668
2-s-w-η-β	16.4055	17.6186	17.4782
4-d-w-u-η-τ	17.0874	16.9623	17.6715
4-d-w-u-η-τ′	16.9469	16.4283	–[b]
1-s-w-η-β	16.2860	17.3093	17.7503
1-s-w-η-β	16.2859	17.3101	–[c]
1-s-r-u-δ	17.3410	17.8787	17.8873
2-s-r-u-b-δ	17.3588	17.7872	18.0168
2-s-w-u-η-α	17.2421	17.7025	18.3159
3-d-r-ξ-ε	17.1206	18.1408	18.3595
3-d-w-η-τ	16.5426	17.6572	18.6644
3-s-w-u-a-τ	17.5923	17.8487	18.8511
1-s-w-u-a-α	18.1246	18.6021	18.9152
4-d-r-ξ-ε	17.3845	18.3894	18.9490
3-d-w-u-η-τ	17.6398	17.6661	18.9549
3-d-w-ξ-τ	17.1062	18.5303	19.2834
2-s-w-a-β	17.9341	19.3124	19.7416
1-s-w-β	18.2414	19.5177	20.2229
2-s-w-u-a-α	18.6787	19.3197	20.5679
4-d-w-u-τ	–[b]	–[b]	20.5947
4-w-ξ-yτ	16.6961	17.6619	20.6825
3-w-ξ-γτ	16.9014	17.6648	20.7340
4-s-w-u-a-τ	18.3888	19.0776	–[d]
3-d-r-b-ε	19.1743	20.3618	20.7350
4-d-r-a-ε	19.3939	20.6685	21.3578
3-d-w-u-a-τ	20.2518	21.0081	22.0232
2-d-w-η	21.5385	23.4237	22.2956
4-s-r-u-b-ε	21.6026	21.8345	–[d]
3-s-r-u-b-ε	21.6422	21.9520	–[d]
3-s-r-u-ε	21.6561	21.8423	–[d]
1-d-w-u-η	22.1174	23.7542	22.7227
1-d-w-ξ	21.9923	23.5416	22.7404
2-d-w-ξ	22.3379	23.8765	22.8871
4-s-w-u-η	22.9262	23.5248	–[a]
3-s-w-u-η	–[e]	–[e]	22.9203
1-s-w-u-η	–[c]	–[c]	23.0548
3-d-r-u-b-ε	22.3306	22.4702	23.1199

1-d-w-η	22.5801	24.2017	23.1482
2-d-w-u-η	23.7754	24.5323	23.2891
2-s-w-u-η	23.7082	24.4540	23.3787
4-d-w-η	22.9417	24.5711	23.5982
4-d-w-ξ	22.9764	24.4531	23.6187
3-d-w-ξ	23.2021	24.7659	23.8842
1-d-w-u-η′	24.5263	25.0076	23.9560
4-d-r-u-ε	22.8092	23.1476	23.9884
3-d-w-η	23.3455	24.9720	24.0045
2-r-ξ-βδ	20.3235	21.3558	24.2020
4-d-w-u-η	24.6798	25.2325	24.2847
1-r-ξ-βδ	21.2174	22.2692	25.0941
3-s-w-u	24.8554	25.6292	–[d]
1-s-w-u	25.1636	25.9749	25.6076
4-r-ξ-γε	21.3780	22.6368	25.5121
3-r-ξ-γε	21.6273	22.7035	25.5930
1-d-w-u	26.0847	26.9187	26.1093

[a] During DFT optimisation of this input, O8H5 rotates ‘downwards’ to form H15···O23, yielding the corresponding γ conformer

[b] During HF optimisation of this input, the prenyl rotate to form the O-H···π interaction, yielding the corresponding η conformer

[c] During HF optimisation of this input, O8H5 rotates ‘downwards’ to form H15···O26, yielding the corresponding β conformer

[d] During DFT optimisation of this input, O12H17 rotates ‘downwards’ yielding the corresponding non-u conformer.

[e] During HF optimisation of this input, O26H27 rotates to the right yielding the corresponding τ conformer.

Table S 2

Comparison of the relative energies for pairs of conformers whose substituents at C3 have symmetric orientations with respect to the plane of the benzene ring

The calculation methods are denoted with the following acronyms: HF for HF/6-31G(d,p), MP2 for MP2/6-31G(d,p)/HF/6-31G(d,p) and DFT for DFT/B3LYP/6-31+G(d,p). The conformers are listed in order of increasing energy referred to the DFT/B3LYP/6-31+G(d,p) results.

conformer	relative energy (kcal/mol)			conformer	relative energy (kcal/mol)		
	HF	MP2	DFT		HF	MP2	DFT
1-d-r-ξ-αδ	0.0000	0.0000	0.000	2-d-r-ξ-αδ	0.0808	0.0455	0.0056
3-s-w-η-γτ	1.2613	1.5050	1.9751	4-s-w-η-γτ	1.5509	1.6652	2.0946
1-d-r-b-αδ	2.0975	2.2841	2.5019	2-d-r-αδ	2.1090	2.3409	2.4743
3-s-w-a-γτ	3.8789	4.5905	5.2980	4-s-w-a-γτ	3.8552	4.6243	5.2593
1-d-r-u-b-αδ	6.5324	5.7952	6.0206	2-d-r-u-αδ	6.4719	5.8000	6.0121
1-s-r-u-a-αδ	9.0545	8.5695	9.3065	2-s-r-u-a-αδ	8.9767	8.4896	9.2157
1-s-r-u-αδ	9.0101	8.5315	9.1833	2-s-r-u-b-αδ	8.9688	8.4091	9.2246
1-d-r-ξ-δ	10.3273	11.7003	11.1736	2-d-r-ξ-δ	10.4798	11.8571	11.2378
3-s-w-η-γ	10.5489	12.0443	11.5954	4-s-w-η-γ	10.8455	12.4383	11.8686
3-s-r-γε	10.5219	11.5154	12.0313	4-s-r-a-γε	10.5259	11.5694	12.0678
3-s-r-b-γε	10.5064	11.6215	12.0749	4-s-r-b-γε	10.4119	11.4888	11.9847
1-s-r-βδ	11.5602	12.5381	12.8348	2-s-r-a-βδ	11.5437	12.5002	12.7916
1-s-r-βδ	11.5602	12.5381	12.8348	2-s-r-b-βδ	11.4859	12.3804	12.8659
1-d-r-b-δ	12.4581	13.9842	13.6519	2-d-r-δ	12.4964	14.1215	13.6027
1-d-w-ξ-α	12.7524	13.4798	13.7472	2-d-w-ξ-α	12.8989	13.5932	13.8252
1-d-w-η-α	13.1572	13.9279	13.9766	2-d-w-η-α	13.8807	15.2904	13.9971
3-s-w-γ	12.5754	14.2318	13.9899	4-s-w-γ	12.5393	14.2836	14.0726
1-d-w-u-η-α	15.4795	15.1379	15.2303	2-d-w-u-η-α	16.3892	16.7274	15.2641
3-s-w-η-u-τ	15.2688	15.2406	15.7472	4-s-w-u-η-τ	15.9070	15.9382	15.6294
1-s-w-u-η-α	16.5636	16.6234	16.3870	2-s-w-u-η-α	17.2421	17.7025	18.3159
1-d-r-u-b-δ	16.6171	17.1811	16.8861	2-d-r-u-δ	16.5753	17.2488	16.8873
3-d-w-η-τ	16.5426	17.6572	18.6644	4-d-w-η-τ	15.6105	16.5531	17.2674
3-d-w-ξ-τ	17.1062	18.5303	19.2834	4-d-w-ξ-τ	15.8319	17.6186	17.4668
1-s-w-η-β	16.2860	17.3093	17.7503	2-s-w-η-β	16.4055	17.3093	17.4782
3-d-w-u-η-τ	17.6398	17.6661	18.9549	4-d-w-u-η-τ	17.0874	16.9623	17.6715
1-s-r-u-δ	17.3410	17.8787	17.8873	2-s-r-u-b-δ	17.3588	17.7872	18.0168
3-d-r-ξ-ε	17.1206	18.1408	18.3595	4-d-r-ξ-ε	17.3845	18.3894	18.9490
1-s-w-u-a-α	18.1246	18.6021	18.9152	2-s-w-u-a-α	18.6787	19.3197	20.5679
1-s-w-β	18.2414	19.5177	20.2229	2-s-w-a-β	17.9341	19.3124	19.7416
3-s-w-u-a-τ	17.5923	17.8487	18.8511	4-s-w-u-a-τ	18.3888	19.0776	–
3-w-ξ-γτ	16.9014	17.6648	20.7340	4-w-ξ-γτ	16.6961	17.6619	20.6825
3-d-r-b-ε	19.1743	20.3618	20.7350	4-d-r-ε	19.3939	20.6685	21.3578
1-d-w-η	22.5801	24.2017	23.1482	2-d-w-η	21.5385	23.4237	22.2956
1-r-ξ-βδ	21.2174	22.2692	25.0941	2-r-ξ-βδ	20.3235	21.3558	24.2020
3-s-r-u-b-ε	21.6422	21.9520	–	4-s-r-u-b-ε	21.6026	21.8345	–
1-d-w-u-η	22.1174	23.7542	22.7227	2-d-w-u-η	23.7754	24.5323	23.2891
1-d-w-ξ	21.9923	23.5416	22.7404	2-d-w-ξ	22.3379	23.8765	22.8871
1-s-w-u-η	–	–	23.0548	2-s-w-u-η	23.7082	24.4540	23.3787
3-d-w-η	23.3455	24.9720	24.0045	4-d-w-η	22.9417	24.5711	23.5982
3-d-w-ξ	23.2021	24.7659	23.8842	4-d-w-ξ	22.9764	24.4531	23.6187

3-d-r-u-b-ε	22.3306	22.4702	23.1199	4-d-r-u-ε	22.8092	23.1476	23.9884
1-r-ξ-βδ	21.2174	22.2692	25.0941	2-r-ξ-βδ	20.3235	21.3558	24.2020
3-r-ξ-γε	21.6273	22.7035	25.5930	4-r-ξ-γε	21.3780	22.6368	25.5121

Table S 3
Parameters of the intramolecular hydrogen bonds in the calculated conformers of arzanol, *in vacuo*.
HF/6-31G(d,p) and DFT for DFT/B3LYP/6-31+G(d,p). The conformers are listed in order of increasing energy referred to the DFT/B3LYP/6-31+G(d,p) results.

conformer	IHB considered	parameters of the IHB					
		HF/6-31G(d,p) results			DFT/B3LYP/6-31+G(d,p) results		
		H···O (Å)	O···O (Å)	$O\hat{H}O$	H···O (Å)	O···O (Å)	$O\hat{H}O$
1-d-r-ξ-αδ	H15···O14	1.633	2.498	147.0	1.482	2.441	152.8
	H27···O8	1.876	2.811	165.9	1.760	2.740	170.8
	H16···O23	1.805	2.752	169.8	1.697	2.683	170.2
2-d-r-ξ-αδ	H15···O14	1.633	2.498	147.0	1.482	2.441	152.8
	H27···O8	1.879	2.813	166.0	1.764	2.743	170.6
	H16···O23	1.807	2.754	169.4	1.701	2.687	169.9
3-s-w-η-γτ	H17···O14	1.658	2.512	146.0	1.528	2.465	151.6
	H15···O23	1.795	2.744	170.9	1.697	2.682	170.5
	H27···O10	1.897	2.826	164.8	1.797	2.769	169.4
4-s-w-η-γτ	H17···O14	1.658	2.512	146.0	1.528	2.466	151.6
	H15···O23	1.796	2.745	170.6	1.700	2.684	170.3
	H27···O10	1.912	2.832	161.8	1.794	2.766	168.9
2-d-r-αδ	H15···O14	1.631	2.496	147.0	1.483	2.441	152.8
	H27···O8	1.880	2.815	166.1	1.765	2.744	170.4
	H16···O23	1.806	2.752	169.1	1.701	2.686	169.7
1-d-r-b-αδ	H15···O14	1.631	2.496	147.0	1.482	2.441	152.8
	H27···O8	1.881	2.816	166.1	1.767	2.746	170.6
	H16···O23	1.806	2.753	169.6	1.701	2.687	169.9
4-s-w-a-γτ	H17···O14	1.663	2.514	145.6	1.533	2.468	151.4
	H15···O23	1.792	2.742	170.8	1.691	2.677	170.6
	H27···O10	1.917	2.840	163.1	1.820	2.790	169.3
3-s-w-a-γτ	H17···O14	1.662	2.514	145.7	1.533	2.468	151.4
	H15···O23	1.790	2.739	170.8	1.690	2.675	170.5
	H27···O10	1.912	2.836	163.3	1.822	2.791	169.1
2-d-r-u-αδ	H15···O14	1.662	2.516	145.7	1.502	2.448	170.4
	H27···O8	1.884	2.818	166.1	1.774	2.751	152.0
	H16···O23	1.809	2.755	169.0	1.703	2.687	169.4
1-d-r-u-b-αδ	H15···O14	1.657	2.512	146.1	1.501	2.448	152.0
	H27···O8	1.887	2.821	165.8	1.774	2.752	170.5
	H16···O23	1.811	2.757	169.5	1.702	2.687	169.6
1-s-r-u-αδ	H17···O14	1.723	2.556	144.0	1.581	2.496	149.8
	H27···O8	1.908	2.837	165.1	1.813	2.784	170.0
	H16···O23	1.809	2.757	170.9	1.703	2.686	170.0
2-s-r-u-a-αδ	H17···O14	1.722	2.556	143.9	1.576	2.493	150.1
	H27···O8	1.909	2.838	165.1	1.814	2.785	170.2
	H16···O23	1.809	2.758	171.2	1.702	2.686	170.4

2-s-r-u-b-αδ	H17···O14	1.724	2.557	143.7	1.582	2.496	149.8
	H27···O8	1.907	2.836	165.1	1.813	2.784	170.0
	H16···O23	1.807	2.756	170.7	1.704	2.687	170.1
1-s-r-u-a-αδ	H17···O14	1.722	2.556	143.9	1.578	2.494	150.0
	H27···O8	1.909	2.837	165.0	1.814	2.786	170.4
	H16···O23	1.808	2.756	170.9	1.704	2.686	169.7
1-d-r-ξ-δ	H15···O14	1.672	2.525	146.1	1.543	2.477	151.6
	H16···O23	1.826	2.747	161.0	1.684	2.658	166.4
2-d-r-ξ-δ	H15···O14	1.673	2.524	146.0	1.543	2.477	151.7
	H16···O23	1.828	2.748	160.7	1.685	2.658	166.1
3-s-w-η-γ	H17···O14	1.662	2.516	146.1	1.530	2.468	151.8
	H15···O23	1.809	2.736	162.7	1.679	2.655	167.3
4-s-w-η-γ	H17···O14	1.661	2.515	146.1	1.530	2.468	151.8
	H15···O23	1.812	2.737	162.3	1.682	2.657	167.1
4-s-r-b-γε	H17···O14	1.675	2.524	145.5	1.548	2.478	151.1
	H15···O23	1.831	2.771	168.1	1.730	2.708	170.9
	H16···O26	2.031	2.849	163.9	1.954	2.906	166.8
3-s-r-γε	H17···O14	1.673	2.523	145.7	1.545	2.477	151.3
	H15···O23	1.832	2.772	168.1	1.732	2.710	170.9
	H16···O26	2.035	2.950	163.1	1.958	2.908	166.1
4-s-r-a-γε	H17···O14	1.673	2.523	145.7	1.545	2.477	151.4
	H15···O23	1.835	2.775	168.1	1.734	2.712	171.0
	H16···O26	2.038	2.955	163.8	1.954	2.908	167.8
3-s-r-b-γε	H17···O14	1.676	2.525	145.5	1.548	2.478	151.1
	H15···O23	1.833	2.773	168.0	1.733	2.711	170.9
	H16···O26	2.040	2.955	162.9	1.952	2.904	166.8
2-s-r-a-βδ	H17···O14	1.677	2.525	145.5	1.553	2.481	151.1
	H15···O26	2.017	2.937	164.3	1.939	2.892	166.9
	H16···O23	1.850	2.789	167.9	1.752	2.727	170.3
1-s-r-βδ	H17···O14	1.680	2.527	145.3	1.557	2.483	150.9
	H15···O26	2.020	2.938	164.0	1.939	2.891	166.8
	H16···O23	1.851	2.790	167.8	1.751	2.726	170.2
2-s-r-b-βδ	H17···O14	1.680	2.527	145.3	1.557	2.483	150.9
	H15···O26	2.015	2.933	164.1	1.936	2.889	167.0
	H16···O23	1.847	2.787	168.8	1.745	2.721	170.6
2-d-r-δ	H15···O14	1.670	2.522	146.0	1.543	2.477	151.7
	H16···O23	1.824	2.744	160.7	1.689	2.661	165.7
1-d-r-b-δ	H15···O14	1.669	2.522	146.0	1.542	2.476	151.7
	H16···O23	1.823	2.745	161.4	1.680	2.654	166.6
1-d-w-ξ-α	H15···O14	1.655	2.511	146.0	1.507	2.454	152.0
	H27···O8	1.812	2.847	167.3	1.769	2.740	169.2
2-d-w-ξ-α	H15···O14	1.653	2.510	146.1	1.506	2.453	152.8
	H27···O8	1.903	2.837	166.8	1.766	2.737	169.0
1-d-w-η-α	H15···O14	1.645	2.502	146.1	1.498	2.448	152.1
	H27···O8	1.898	2.832	166.6	1.761	2.732	168.7
3-s-w-γ	H17···O14	1.665	2.518	145.9	1.535	2.471	151.6
	H15···O23	1.808	2.736	162.7	1.676	2.653	167.7
2-d-w-η-α	H15···O14	1.638	2.499	146.7	1.501	2.449	152.0
	H27···O8	1.942	2.802	149.7	1.776	2.747	168.1

4-s-w-γ	H17···O14	1.667	2.519	145.8	1.537	2.472	151.5
	H15···O23	1.812	2.738	162.6	1.684	2.659	167.0
1-d-w-u-η-α	H15···O14	1.677	2.524	145.1	1.523	2.460	151.2
	H27···O8	1.913	2.845	166.5	1.884	2.788	168.5
2-d-w-u-η-α	H15···O14	1.671	2.521	145.5	1.524	2.459	151.2
	H27···O8	1.953	2.807	148.7	1.786	2.755	168.8
4-s-w-u-η-τ	H17···O14	1.713	2.551	144.3	1.555	2.479	150.5
	H27···O10	1.995	2.827	145.5	1.884	2.788	167.7
3-s-w-η-u-τ	H17···O14	1.540	2.512	144.2	1.553	2.477	150.6
	H27···O10	1.951	2.883	167.1	1.820	2.786	168.8
1-s-w-u-η-α	H17···O14	1.706	2.544	144.2	1.557	2.480	150.5
	H27···O8	1.983	2.911	165.6	1.842	2.806	168.6
1-d-r-u-b-δ	H15···O14	1.700	2.543	145.0	1.556	2.481	151.1
	H16···O23	1.827	2.749	161.3	1.680	2.655	166.4
2-d-r-u-δ	H15···O14	1.705	2.545	144.6	1.554	2.480	151.1
	H16···O23	1.826	2.746	160.8	1.688	2.660	165.7
4-d-w-η-τ	H15···O14	1.693	2.528	143.7	1.564	2.483	149.7
	H27···O10	1.961	2.888	145.4	1.842	2.804	168.0
4-d-w-ξ-τ	H15···O14	1.700	2.535	143.7	1.571	2.489	149.7
	H27···O10	1.977	2.904	165.6	1.857	2.818	167.8
2-s-w-η-β	H17···O14	1.665	2.518	146.0	1.538	2.471	151.5
	H15···O26	2.037	2.930	157.1	1.937	2.868	159.9
4-d-w-u-η-τ	H15···O14	1.736	2.558	142.3	1.586	2.494	148.8
	H27···O10	1.952	2.881	166.1	1.833	2.796	168.3
4-d-w-u-η-τ′	H15···O14	1.738	2.560	142.3	1.584	2.493	148.9
	H27···O10	1.970	2.897	165.3	1.868	2.828	167.5
1-s-w-η-β	H17···O14	1.668	2.520	145.9	1.539	2.472	151.5
	H15···O26	2.039	2.886	148.2	1.947	2.835	151.0
1-s-r-u-δ	H17···O14	1.723	2.557	144.0	1.582	2.498	149.9
	H16···O23	1.832	2.755	161.9	1.694	2.667	167.0
2-s-r-u-b-δ	H17···O14	1.724	2.558	144.0	1.582	2.497	149.9
	H16···O23	1.827	2.753	162.8	1.684	2.660	167.7
2-s-w-u-η-α	H17···O14	1.704	2.543	144.5	1.559	2.481	150.5
	H27···O8	2.062	2.836	137.8	1.874	2.764	150.6
3-d-r-ξ-ε	H15···O14	1.676	2.524	145.5	1.547	2.477	151.1
	H16···O26	2.033	2.933	158.9	1.926	2.865	162.1
3-d-w-η-τ	H15···O14	1.685	2.527	145.6	1.555	2.478	150.3
	H27···O10	2.031	2.833	141.3	1.852	2.752	151.8
3-s-w-u-a-τ	H17···O14	1.705	2.543	144.5	1.560	2.482	156.3
	H27···O10	1.973	2.904	167.0	1.852	2.814	168.0
1-s-w-u-a-α	H17···O14	1.705	2.542	144.1	1.562	2.483	150.3
	H27···O8	1.951	2.875	164.4	1.837	2.802	168.7
4-d-r-ξ-ε	H15···O14	1.692	2.532	144.3	1.562	2.485	150.3
	H16···O26	2.070	2.889	144.2	1.963	2.830	147.4
3-d-w-u-η-τ	H15···O14	1.730	2.561	143.5	1.576	2.489	149.5
	H27···O10	2.016	2.829	142.8	1.840	2.748	153.3
3-d-w-ξ-τ	H15···O14	1.688	2.530	144.8	1.560	2.483	150.4
	H27···O10	2.049	2.843	140.5	1.931	2.787	145.4
2-s-w-a-β	H17···O14	1.672	2.522	145.6	1.543	2.475	151.3

	H15···O26	2.050	2.937	156.0	1.947	2.876	159.4
1-s-w-β	H17···O14	1.671	2.521	145.6	1.542	2.474	151.3
	H15···O26	2.032	2.880	148.4	1.934	2.828	151.9
2-s-w-u-a-α	H17···O14	1.713	2.548	144.0	1.565	2.485	150.2
	H27···O8	2.072	2.845	137.8	1.885	2.771	149.7
4-d-w-u-τ	H15···O14	–[a]	–[a]	–[a]	1.574	2.487	149.5
	H27···O10				1.916	2.785	147.3
4-w-ξ-γτ	H15···O23	1.817	2.767	172.6	1.720	2.705	172.6
	H27···O10	1.902	2.832	165.2	1.805	2.778	170.4
3-w-ξ-γτ	H15···O23	1.823	2.769	169.9	1.733	2.715	171.0
	H27···O10	1.910	2.832	162.7	1.810	2.782	169.8
4-s-w-u-a-τ	H17···O14	1.715	2.551	144.1	–[b]	–[b]	–[b]
	H27···O10	2.017	2.842	144.5			
3-d-r-b-ε	H15···O14	1.673	2.521	145.4	1.546	2.476	151.1
	H16···O26	2.032	2.934	159.2	1.924	2.875	162.3
4-d-r-a-ε	H15···O14	1.690	2.530	144.3	1.561	2.485	153.3
	H16···O26	2.078	2.892	143.3	1.981	2.841	146.4
3-d-w-u-a-τ	H15···O14	1.729	2.560	143.6	1.574	2.487	149.5
	H27···O10	2.046	2.845	141.2	1.916	2.785	147.3
2-d-w-η	H15···O14	1.671	2.522	146.0	1.547	2.478	151.4
4-s-r-u-b-ε	H17···O14	1.722	2.557	144.1	–[b]	–[b]	–[b]
	H16···O26	2.035	2.936	159.1			
3-s-r-u-b-ε	H17···O14	1.722	2.557	144.1	–[b]	–[b]	–[b]
	H16···O26	2.042	2.940	158.3			
3-s-r-u-ε	H17···O14	1.721	2.557	144.2	–[b]	–[b]	–[b]
	H16···O26	2.036	2.936	158.8			
1-d-w-u-η	H15···O14	1.713	2.550	144.4	1.565	2.485	150.4
1-d-w-ξ	H15···O14	1.678	2.527	145.8	1.554	2.482	151.1
2-d-w-ξ	H15···O14	1.678	2.528	145.8	1.554	2.482	151.1
4-s-w-u-η	H17···O14	1.713	2.552	144.5	–[b]	–[b]	–[b]
3-s-w-u-η	H17···O14	–[a]	–[a]	–[a]	1.555	2.480	150.8
1-s-w-u-η	H17···O14	1.678	2.527	145.8	1.554	2.482	151.1
3-d-r-u-b-ε	H15···O14	1.713	2.546	143.6	1.560	2.481	150.3
	H16···O26	2.032	2.932	158.7	1.926	2.864	161.8
1-d-w-η	H15···O14	1.670	2.520	145.8	1.546	2.476	151.2
2-d-w-u-η	H15···O14	1.708	2.547	144.6	1.567	2.488	150.6
2-s-w-u-η	H17···O14	1.703	2.544	144.7	1.554	2.479	150.9
4-d-w-η	H15···O14	1.688	2.529	144.4	1.558	2.482	150.4
4-d-w-ξ	H15···O14	1.697	2.536	144.4	1.566	2.489	150.3
3-d-w-ξ	H15···O14	1.683	2.529	145.3	1.554	2.481	151.0
1-d-w-u-η	H15···O14	1.717	2.553	144.4	1.564	2.485	150.5
4-d-r-u-ε	H15···O14	1.726	2.553	142.9	1.574	2.489	149.5
	H16···O26	2.007	2.889	143.1	1.982	2.838	145.7
3-d-w-η	H15···O14	1.679	2.525	145.3	1.546	2.475	151.0
2-r-ξ-βδ	H15···O26	2.013	2.939	167.1	1.937	2.895	169.8
	H16···O23	1.854	2.793	168.3	1.752	2.729	171.7
4-d-w-u-η	H15···O14	1.727	2.554	143.0	1.578	2.492	149.7
1-r-ξ-βδ	H15···O26	2.063	2.969	160.7	1.969	2.921	167.0
	H16···O23	1.874	2.808	166.4	1.760	2.735	171.1

3-s-w-u	H17···O14	1.716	2.554	144.3	–[b]	–[b]	–[b]
1-s-w-u	H17···O14	1.703	2.543	144.4	1.557	2.480	150.5
4-r-ξ-γε	H15···O23	1.860	2.797	167.4	1.754	2.732	172.6
	H16···O26	2.036	2.957	165.0	1.954	2.909	168.1
3-r-ξ-γε	H15···O23	1.872	2.804	166.8	1.767	2.742	170.8
	H16···O26	2.056	2.970	162.9	1.957	2.912	168.5
1-d-w-u	H15···O14	1.717	2.553	144.4	1.564	2.485	150.5

[a] During HF optimisation of this input, O10H16 rotates ‘to the right’ yielding the corresponding r conformer

[b] During DFT optimisation of this input, O12H17 rotates ‘downwards’ yielding the corresponding non-u conformer.

Table S 4

Distance between the H atom of the phenol OH engaged in the O–H···π interaction with the C29=C30 double bond in the prenyl chain and the two C atoms forming the double bond.

The H atom is H16 for η-conformers and H17 for ξ-conformers.

HF/6-31G(d,p) and DFT/B3LYP/6-31+G(d,p) results in vacuo, respectively denoted as HF and DFT in the column headings.

conformer	H16···C29 (η) or H17···C29 (ξ) (Å)		H16···C30 (η) or H17···C30 (ξ) (Å)	
	HF	DFT	HF	DFT
1-d-r-ξ-αδ	2.278	2.104	2.604	2.477
2-d-r-ξ-αδ	2.264	2.086	2.595	2.460
1-d-r-ξ-δ	2.288	2.116	2.612	2.490
2-d-r-ξ-δ	2.276	2.100	2.605	2.488
1-d-w-ξ-α	2.338	2.130	2.677	2.510
2-d-w-ξ-α	2.335	2.116	2.679	2.508
4-d-w-ξ-τ	2.355	2.132	2.698	2.525
3-d-r-ξ-ε	2.291	2.111	2.621	2.491
4-d-r-ξ-ε	2.277	2.105	2.618	2.500
3-d-w-ξ-τ	2.356	2.132	2.700	2.525
4-w-ξ-γτ	2.422	2.220	2.735	2.556
3-w-ξ-γτ	2.415	2.185	2.719	2.538
1-d-w-ξ	2.356	2.165	2.698	2.539
2-d-w-ξ	2.351	2.143	2.690	2.527
4-d-w-ξ	2.363	2.162	2.710	2.537
3-d-w-ξ	2.366	2.171	2.712	2.537
2-r-ξ-βδ	2.345	2.216	2.637	2.507
1-r-ξ-βδ	2.321	2.143	2.624	2.495
4-r-ξ-γε	2.363	2.184	2.654	2.514
3-r-ξ-γε	2.350	2.155	2.653	2.511
3-s-w-η-γτ	2.253	2.071	2.565	2.426
4-s-w-η-γτ	2.241	2.054	2.573	2.429
3-s-w-η-γ	2.320	2.138	2.640	2.502
4-s-w-η-γ	2.314	2.135	2.656	2.523
1-d-w-η-α	2.398	2.152	2.716	2.509
2-d-w-η-α	2.393	2.157	2.742	2.513
1-d-w-u-η-α	2.303	2.121	2.626	2.476
2-d-w-u-η-α	2.306	2.111	2.652	2.466
4-s-w-u-η-τ	2.262	2.058	2.577	2.421
3-s-w-u-η-τ	2.254	2.067	2.577	2.435
1-s-w-u-η-α	2.310	2.112	2.617	2.469
4-d-w-η-τ	2.341	2.110	2.669	2.471
2-s-w-η-β	2.316	2.126	2.647	2.492

4-d-w-u-η-τ	2.255	2.073	2.585	2.432
4-d-w-u-η-τ’	2.264		2.596	
1-s-w-η-β	2.321	2.130	2.622	2.486
3-d-w-u-η-τ	2.283	2.078	2.586	2.428
2-d-w-η	2.397	2.183	2.748	2.560
1-d-w-u-η	2.317	2.134	2.648	2.491
4-s-w-u-η	2.317		2.652	
3-s-w-u-η		2.118		2.491
2-s-w-u-η-α	2.314	2.098	2.645	2.461
3-d-w-η-τ	2.354	2.122	2.675	2.472
1-d-w-η	2.416	2.196	2.719	2.538
2-d-w-u-η	2.311	2.132	2.661	2.498
2-s-w-u-η	2.320	2.112	2.654	2.484
4-d-w-η	2.397	2.194	2.734	2.567
1-s-w-u-η	2.322	2.126	2.622	2.484
1-d-w-η’	2.409	2.185	2.740	2.545
3-d-w-η	2.403	2.200	2.758	2.580
4-d-w-u-η	2.303	2.126	2.637	2.500

Table S 5

Torsion angle between the two ring systems (C3-C9-C17-C18 torsion angle) for the calculated conformers of arzanol.

HF/6-31G(d,p) and DFT/B3LYP/6-31+G(d,p) results in vacuo, respectively denoted as HF and DFT in the column headings.

conformer	C3-C9-C17 angle		conformer	C3-C9-C17 angle	
	HF	DFT		HF	DFT
1-d-r-ξ-αδ	-89.2	-90.3	2-s-r-u-b-δ	70.5	91.9
2-d-r-ξ-αδ	89.4	90.4	2-s-w-u-η-α	77.8	81.7
3-s-w-η-γτ	88.4	90.0	3-d-r-ξ-ε	74.5	77.2
4-s-w-η-γτ	-87.1	-89.9	3-d-w-η-τ	78.2	82.7
2-d-r-αδ	89.7	90.4	3-s-w-u-a-τ	114.9	114.8
1-d-r-b-αδ	-89.5	-90.2	1-s-w-u-a-α	-114.6	-115.7
4-s-w-a-γτ	-87.5	-89.9	4-d-r-ξ-ε	-116.9	-117.9
3-s-w-a-γτ	88.0	89.9	3-d-w-u-η-τ	78.2	82.8
2-d-r-u-αδ	90.2	90.8	3-d-w-ξ-τ	77.4	79.7
1-d-r-u-b-αδ	-89.8	-90.7	2-s-w-a-β	73.9	75.9
1-s-r-u-αδ	-89.9	-91.4	1-s-w-β	-115.2	-114.7
2-s-r-u-a-αδ	90.0	91.4	2-s-w-u-a-α	77.0	81.0
2-s-r-u-b-αδ	70.5	91.9	4-d-w-u-τ	-116.4	-118.2
1-s-r-u-a-αδ	-89.9	-91.9	4-w-ξ-γτ	-88.0	-91.1
1-d-r-ξ-δ	-69.9	-69.9	3-w-ξ-γτ	87.7	90.5
2-d-r-ξ-δ	70.0	72.9	4-s-w-u-a-τ	-77.7	–[a]
3-s-w-η-γ	70.0	71.9	3-d-r-b-ε	74.7	77.2
4-s-w-η-γ	-69.6	-72.1	4-d-r-a-ε	-116.0	-117.0
4-s-r-b-γε	-78.9	-81.5	3-d-w-u-a-τ	77.4	80.4
3-s-r-γε	78.9	81.4	2-d-w-η	62.2	59.2
4-s-r-a-γε	-78.7	-81.9	4-s-r-u-b-ε	-74.1	–[a]
3-s-r-b-γε	78.7	81.9	3-s-r-u-b-ε	73.9	–[a]
2-s-r-a-βδ	80.3	83.3	3-s-r-u-ε	74.1	–[a]
1-s-r-βδ	-79.9	-83.3	1-d-w-u-η	64.1	59.8
2-s-r-b-βδ	80.4	83.7	1-d-w-ξ	-117.5	-122.2
1-s-r-βδ’	-79.9	-83.5	2-d-w-ξ	118.3	121.7
4-s-w-η-γ’		-72.6	4-s-w-u-η		-72.6
2-d-r-δ	70.5	72.8	3-s-w-u-η		123.6
1-d-r-b-δ	111.8	-73.6	1-s-w-u-η	-115.7	-123.4
1-d-w-ξ-α	-114.3	-113.6	3-d-r-u-b-ε	74.5	76.9
2-d-w-ξ-α	113.9	113.2	1-d-w-η′	-118.2	-122.4
1-d-w-η-α	-113.2	-112.6	2-d-w-u-η	-119.1	58.5
3-s-w-γ	70.1	72.7	2-s-w-u-η	61.9	-126.7
2-d-w-η-α	79.7	115.0	4-d-w-η	-120.9	-128.1
4-s-w-γ	-69.7	-71.4	4-d-w-ξ	-121.0	-127.2
1-d-w-u-η-α	-114.2	-113.9	3-d-w-ξ	60.5	51.1

2-d-w-u-η-α	79.6	115.5	1-d-w-u-η′	-118.6	
4-s-w-u-η-τ	-78.3	-114.5	4-d-r-u-ε	-116.6	-117.3
3-s-w-u-η-τ	114.7	114.6	3-d-w-η	54.8	50.9
1-s-w-u-η-α	-115.5	-116.9	2-r-ξ-βδ	79.9	82.8
1-d-r-u-b-δ	-70.6	-74.1	4-d-w-u-η	-120.6	-127.6
2-d-r-u-δ	70.6	73.5	1-r-ξ-βδ	-77.5	-127.6
4-d-w-η-τ	-116.9	-118.6	3-s-w-u	61.5	72.7
4-d-w-ξ-τ	-117.3	-119.1	1-s-w-u	-118.5	-123.4
2-s-w-η-β	74.5	76.6	4-r-ξ-γε	-79.4	-82.0
4-d-w-u-η-τ	-116.6	-117.8	3-r-ξ-γε	78.3	82.2
1-s-w-η-β	-115.7	-115.7	1-d-w-u	-118.0	-121.0
1-s-r-u-δ	-69.9	-72.9			

[a] During DFT optimisation of this input, O12H17 rotates ‘downwards’ yielding the corresponding non-u conformer.

Table S 6
Bond angle of the methylene bridge (C3-C9-C17) for the calculated conformers of arzanol.
HF/6-31G(d,p) and DFT/B3LYP/6-31+G(d,p) results in vacuo, respectively denoted as HF and DFT in the column headings.

conformer	C3-C9-C17 angle		conformer	C3-C9-C17 angle	
	HF	DFT		HF	DFT
1-d-r-ξ-αδ	117.0	116.5	2-s-r-u-b-δ	115.9	116.7
2-d-r-ξ-αδ	117.0	116.2	2-s-w-u-η-α	115.8	116.7
3-s-w-η-γτ	117.0	116.9	3-d-r-ξ-ε	114.9	115.5
4-s-w-η-γτ	117.1	116.8	3-d-w-η-τ	115.8	116.8
2-d-r-αδ	117.0	116.3	3-s-w-u-a-τ	115.9	116.1
1-d-r-b-αδ	117.0	116.3	1-s-w-u-a-α	115.6	116.0
4-s-w-a-γτ	117.1	116.9	4-d-r-ξ-ε	116.0	119.5
3-s-w-a-γτ	117.1	116.9	3-d-w-u-η-τ	115.7	116.8
2-d-r-u-αδ	117.0	116.5	3-d-w-ξ-τ	115.9	116.8
1-d-r-u-b-αδ	117.0	117.0	2-s-w-a-β	114.7	115.1
1-s-r-u-αδ	117.1	116.8	1-s-w-β	116.0	116.6
2-s-r-u-a-αδ	117.0	116.9	2-s-w-u-a-α	115.4	116.5
2-s-r-u-b-αδ	117.0	116.8	4-d-w-u-τ	–	116.0
1-s-r-u-a-αδ	117.1	116.9	4-w-ξ-yτ	116.8	116.6
1-d-r-ξ-δ	115.4	116.2	3-w-ξ-γτ	116.9	116.6
2-d-r-ξ-δ	115.4	116.1	4-s-w-u-a-τ	115.3	–[a]
3-s-w-η-γ	115.6	116.4	3-d-r-b-ε	114.8	115.4
4-s-w-η-γ	115.7	116.4	4-d-r-a-ε	116.0	116.6
4-s-r-b-γε	116.1	116.6	3-d-w-u-a-τ	115.7	116.5
3-s-r-γε	116.1	116.6	2-d-w-η	114.0	113.9
4-s-r-a-γε	116.1	116.7	4-s-r-b-ε	114.2	–[a]
3-s-r-b-γε	116.1	116.7	3-s-r-u-b-ε	114.3	–[a]
2-s-r-a-βδ	116.6	116.9	3-s-r-u-ε	114.3	–[a]
1-s-r-βδ	116.6	117.0	1-d-w-η	115.2	115.7
2-s-r-b-βδ	116.6	116.9	1-d-w-ξ	115.3	115.7
1-s-r-βδ’	116.5	117.0	2-d-w-ξ	115.3	115.5
2-d-r-δ	115.5	116.0	4-s-w-u-η		116.4
1-d-r-b-δ	115.5	116.3	3-s-w-u-η		115.7
1-d-w-ξ-α	115.8	116.4	1-s-w-u-η	116.0	115.5
2-d-w-ξ-α	115.8	116.3	3-d-r-u-b-ε	114.7	115.2
1-d-w-η-α	115.8	116.1	1-d-w-η′	115.1	115.6
3-s-w-γ	115.7	116.5	2-d-w-u-η	114.2	114.2
2-d-w-η-α	115.7	116.4	2-s-w-u-η	114.7	115.1
4-s-w-γ	115.7	116.4	4-d-w-η	115.2	116.0
1-d-w-u-η-α	115.7	116.2	4-d-w-ξ	115.2	115.9
2-d-w-u-η-α	115.9	116.3	3-d-w-ξ	114.8	115.6
4-s-w-u-η-τ	115.4	116.3	1-d-w-u-η′	115.1	115.5

3-s-w-u-η-τ	115.9	116.2	4-d-r-u-ε	116.0	116.7
1-s-w-u-η-α	115.7	115.9	3-d-w-η	114.8	115.7
1-d-r-u-b-δ	115.6	116.4	2-r-ξ-βδ	115.6	115.9
2-d-r-u-δ	115.6	116.3	4-d-w-u-η	115.2	116.0
4-d-w-η-τ	115.5	116.1	1-r-ξ-βδ	115.8	116.4
4-d-w-ξ-τ	115.5	116.0	3-s-w-u	114.1	–[a]
2-s-w-η-β	114.9	115.3	1-s-w-u	115.0	115.6
4-d-w-u-η-τ	115.6	116.2	4-r-ξ-γε	115.9	116.4
1-s-w-η-β	116.0	116.6	3-r-ξ-γε	115.9	116.4
1-s-r-u-δ	115.8	116.7	1-d-w-u	115.2	115.5

[a] During DFT optimisation of this input, O12H17 rotates ‘downwards’ yielding the corresponding non-u conformer.

Table S 7

Calculated vibrational frequencies (harmonic approximation) of the O—H bonds in the arzanol molecule.

DFT/B3LYP/6-31+G(d,p) results in vacuo. The frequency values have been scaled by the factor 0.964, recommended for DFT/B3LYP/6-31+G(d,p) calculations [21]. When the same two values are present for O10—H16 and O26—H27, they correspond to symmetric and asymmetric vibrations of the two groups.

conformer	vibrational frequencies (cm^{-1})			
	O8—H15	O10—H16	O12—H17	O26—H27
1-d-r-ξ-αδ	2583.26	3117.48	3529.19	3273.01
2-d-r-ξ-αδ	2588.21	3121.83	3472.68	3275.33
3-s-w-η-γτ	3144.66	3329.79<br>3374.52	2828.07	3329.79<br>3374.52
4-s-w-η-γτ	3148.69	3414.40<br>3339.53	2827.76	3414.40<br>3339.53
2-d-r-αδ	2592.88	3119.99	3673.72	3281.18
1-d-r-b-αδ	2587.99	3121.45	3673.01	3282.67
4-s-w-a-γτ	3136.83	3669.33	2840.52	3406.24
3-s-w-a-γτ	3137.47	3671.45	2842.14	3414.64
2-d-r-u-αδ	2706.56	3128.77	3737.93	3304.15
1-d-r-u-b-αδ	2702.04	3126.07	3737.48	3301.60
1-s-r-u-αδ	3707.06	3165.67	3036.81	3411.43
2-s-r-u-a-αδ	3705.30	3160.98	3029.02	3414.57
2-s-r-u-b-αδ	3705.83	3165.12	3037.32	3412.83
1-s-r-u-a-αδ	3706.50	3166.22	3033.90	3410.63
1-d-r-ξ-δ	2877.93	3191.10	3496.88	3698.00
2-d-r-ξ-δ	2880.92	3192.54	3494.55	3697.30
3-s-w-η-γ	3193.80	3507.29	2832.39	3696.16
4-s-w-η-γ	3201.69	3522.23	2831.96	3696.90
4-s-r-b-γε	3287.71	3618.93	2900.46	3685.22
3-s-r-γε	3289.40	3621.02	2894.79	3686.65
4-s-r-a-γε	3291.27	3616.60	2913.36	3686.87
3-s-r-b-γε	3291.09	3617.67	2902.76	3686.35
2-s-r-a-γε	3609.40	3327.25	2931.13	3686.28
1-s-r-γε	3609.29	3326.62	2939.49	3685.91
2-s-r-b-γε	3609.78	3322.51	2941.65	3686.58
1-s-r-γε	3608.56	3330.54	2934.51	3686.34
4-s-w-η-γ ′	3198.77	3503.89	2831.03	3695.50
2-d-r-δ	2879.96	3195.05	3676.66	3696.08
1-d-r-b-δ	2878.49	3185.71	3676.22	3697.15
1-d-w-ξ-α	2710.08	3696.99	3510.32	3362.71
2-d-w-ξ-α	2706.11	3696.73	3504.64	3358.17
1-d-w-η-α	2681.69	3544.74	3682.07	3352.54
3-s-w-a-γ	3191.02	3677.88	2845.44	3696.98

2-d-w-η-α	2691.69	3541.13	3683.39	3371.08
4-s-w-γ	3201.93	3682.63	2855.17	3695.30
1-d-w-u-η-α	2805.91	3502.21	3732.36	3377.85
2-d-w-u-η-α	2810.47	3494.63	3731.36	3391.18
4-s-w-u-η-τ	3736.78	3445.26<br>3690.90	2950.91	3445.26<br>3690.90
3-s-w-u-η-τ	3738.17	3418.79	2950.55	3473.37
1-s-w-u-η-α	3710.79	3483.45	2963.69	3485.47
1-d-r-u-b-δ	2956.21	3188.49	3739.76	3696.98
2-d-r-u-δ	2953.85	3197.11	3739.26	3696.08
4-d-w-η-τ	2960.44	3512.80<br>3482.06	3688.20	3512.80<br>3482.06
4-d-w-ξ-τ	2976.74	3687.62	3520.74	3505.34
2-s-w-η-β	3592.87	3510.51	2869.77	3690.03
4-d-w-u-η-τ	3054.79	3436.94	3729.27	3493.07
1-s-w-η-β	3594.18	3499.97	2870.69	3692.55
1-s-r-u-δ	3728.38	3221.98	3029.16	3695.82
2-s-r-u-b-δ	3729.21	3209.72	3030.13	3695.89
2-s-w-u-η-α	3712.79	3491.16	2969.08	3532.13
3-d-r-ξ-ε	2901.68	3585.07	3505.94	3690.89
3-d-w-η-τ	2942.05	3505.32<br>3480.50	3689.96	3505.32<br>3480.50
3-s-w-u-a-τ	3737.82	3674.71	2970.03	3493.76
1-s-w-u-a-α	3710.19	3681.84	2975.75	3480.85
4-d-r-ξ-ε	2940.52	3600.89	3502.40	3694.37
3-d-w-u-η-τ	3041.74	3433.44	3730.39	3491.44
3-d-w-ξ-τ	2951.82	3691.11	3529.66	3568.24
2-s-w-a-β	3596.76	3693.11	2884.22	3690.91
1-s-w-β	3590.00	3682.63	2879.35	3691.28
2-s-w-u-a-α	3712.03	3693.12	2987.51	3537.58
4-d-w-u-τ	3053.58	3678.69	3729.43	3511.11
4-w-ξ-yτ	3210.13	3689.11	3576.81	3396.08
3-w-ξ-γτ	3229.96	3690.29	3566.78	3399.00
3-d-r-b-ε	2897.53	3583.46	3681.72	3689.86
4-d-r-ε	2939.74	3602.37	3681.49	3691.58
3-d-w-u-a-τ	3032.82	3676.58	3728.95	3559.27
2-d-w-η	2907.16	3568.67	3686.22	3701.45
1-d-w-η	2914.63	3559.26	3687.51	3699.56
1-d-w-ξ	2932.14	3700.09	3536.51	3698.37
2-d-w-ξ	2933.95	3698.37<br>3698.07	3531.95	3698.37<br>3698.07
3-s-w-u-η	3736.22	3499.96	2950.92	3697.29
1-s-w-u-η	3740.51	3498.89	2953.22	3695.76
3-d-r-u-b-ε	2981.58	3584.55	3736.01	3689.10
1-d-w-η′	2912.48	3565.67	3687.38	3698.98

2-d-w-u-η	2992.42	3522.51	3734.63	3691.85
2-s-w-u-η	3740.14	3504.83	2956.81	3700.68
4-d-w-η	2943.78	3563.53	3687.48	3697.07
4-d-w-ξ	2963.58	3700.07	3541.50	3698.39
3-d-w-ξ	2931.87	3547.21	3695.17	3701.42
1-d-w-u-η	2998.88	3521.73	3734.03	3698.62
4-d-r-u-ε	3014.72	3605.72	3736.96	3692.09
3-d-w-η	2913.83	3568.74	3691.36	3702.00
2-r-ξ-βδ	3623.01	3331.55	3533.36	3685.45
4-d-w-u-η	3028.86	3515.87	3733.25	3696.80
1-r-ξ-βδ	3633.84	3342.17	3526.81	3688.78
4-r-ξ-γε	3341.70	3624.15	3556.07	3688.59
3-r-ξ-γ-ε	3356.51	3626.56	3549.69	3687.47
1-s-w-u	3740.92	3681.23	2961.39	3695.47
1-d-w-u	3003.97	3688.93	3736.63	3697.37

Table S 8

Red shifts in the calculated vibrational frequencies (harmonic approximation) of the O—H bonds when they are engaged in intramolecular hydrogen bonds (IH).

DFT/B3LYP/6-31+G(d,p) results in vacuo. The frequency values have been scaled by the factor 0.964, recommended for DFT/B3LYP/6-31+G(d,p) calculations [21]. The red shifts are evaluated with reference to the average frequency of the same OH when not engaged in IHBs, taken from the conformers in which it is free.

a) Red-shifts of O8—H15

Average value of the frequency of O8—H15 when not engaged in IHBs (s-r-u and s-w-u-conformers): 3807.265 cm^{-1}

for the H15···O14 IHB		for the H15···O23 IHB		for the H15···O26 IHB	
conformer	red shift (cm^{-1})	conformer	red shift (cm^{-1})	conformer	red shift (cm^{-1})
1-d-r-ξ-αδ	1165.86	3-s-w-η-γτ	591.82	2-s-r-a-βδ	116.62
2-d-r-ξ-αδ	1160.79	4-s-w-η-γτ	587.70	1-s-r-βδ	116.73
2-d-r-αδ	1156.02	4-s-w-a-γτ	333.69	2-s-r-b-βδ	116.24
1-d-r-b-αδ	1161.02	3-s-w-a-γτ	599.17	1-s-r-βδ′	117.48
2-d-r-u-αδ	1039.79	3-s-w-η-γ	541.58	2-s-w-η-β	133.53
1-d-r-u-b-αδ	1044.40	4-s-w-η-γ	533.50	1-s-w-η-β	132.19
1-d-r-ξ-δ	864.56	4-s-r-b-γε	445.55	2-s-w-a-β	129.54
2-d-r-ξ-δ	861.50	3-s-r-γε	443.83	1-s-w-β	136.45
2-d-r-δ	862.48	4-s-r-a-γε	441.91	2-r-ξ-βδ	102.70
1-d-r-b-δ	863.98	3-s-r-b-γε	442.10	1-r-ξ-βδ	91.63
1-d-w-ξ-α	1036.18	4-s-w-η-γ ′	536.49		
2-d-w-ξ-α	1040.24	3-s-w-γ	544.42		
1-d-w-η-α	1065.21	4-s-w-γ	533.27		
2-d-w-η-α	1054.99	4-w-ξ-yτ	524.88		
1-d-w-u-η-α	938.20	3-w-ξ-γτ	504.60		
2-d-w-u-η-α	933.53	4-r-ξ-γε	390.35		
1-d-r-u-b-δ	784.51	3-r-ξ-γ-ε	375.20		
2-d-r-u-δ	786.93				
4-d-w-η-τ	780.19				
4-d-w-ξ-τ	763.53				
4-d-w-u-η-τ	683.71				
3-d-r-ξ-ε	840.27				
3-d-w-η-τ	798.99				
4-d-r-ξ-ε	800.56				
3-d-w-u-η-τ	697.06				
3-d-w-ξ-τ	789.01				
4-d-w-u-η-τ	684.96				
3-d-r-b-ε	844.51				
4-d-r-ε	801.36				
3-d-w-u-a-τ	706.18				

2-d-w-η	834.66				
1-d-w-ξ	809.13				
2-d-w-ξ	807.27				
3-d-r-u-b-ε	758.57				
1-d-w-η′	829.23				
2-d-w-u-η	747.49				
4-d-w-η	797.23				
4-d-w-ξ	776.98				
3-d-w-ξ	809.40				
1-d-w-u-η	740.89				
4-d-r-u-ε	724.69				
3-d-w-η	827.84				
4-d-w-u	710.23				
1-d-w-u	735.68				

b) Red-shifts of O10—H16

Average value of the frequency of when not engaged in IHBs (w conformers without O–H16···π interaction): 3769.815 cm^{-1}

for the H16···O23 IHB		for the H16···O26 IHB		for the H16···π IHB	
conformer	red shift (cm^{-1})	conformer	red shift (cm^{-1})	conformer	red shift (cm^{-1})
1-d-r-ξ-αδ	582.16	4-s-r-b-γε	69.42	3-s-w-η-γτ	365.07
2-d-r-ξ-αδ	577.72	3-s-r-γε	67.28	4-s-w-η-γτ	278.56
2-d-r-αδ	579.60	4-s-r-a-γε	71.80	3-s-w-η-γ	183.58
1-d-r-b-αδ	578.10	3-s-r-b-γε	70.70	4-s-w-η-γ	168.30
2-d-r-u-αδ	570.62	3-d-r-ξ-ε	104.05	4-s-w-η-γ ′	187.06
1-d-r-u-b-αδ	573.38	4-d-r-ξ-ε	87.88	1-d-w-η-α	145.28
1-s-r-u-αδ	532.89	3-d-r-b-ε	105.70	2-d-w-η-α	148.98
2-s-r-u-a-αδ	537.69	4-d-r-ε	86.36	1-d-w-u-η-α	188.77
2-s-r-u-b-αδ	533.45	3-d-r-u-b-ε	104.58	2-d-w-u-η-α	196.52
1-s-r-u-a-αδ	532.33	4-d-r-u-ε	82.93	4-s-w-u-η-τ	247.01
1-d-r-ξ-δ	506.89	4-r-ξ-γε	64.09	3-s-w-u-η-τ	274.07
2-d-r-ξ-δ	505.42	3-r-ξ-γ-ε	61.63	1-s-w-u-η-α	207.95
2-s-r-a-βδ	367.68	3-d-r-u-b-ε	84.796	4-d-w-η-τ	177.95
1-s-r-βδ	368.32	4-d-r-u-ε	63.156	2-s-w-η-β	180.29
2-s-r-b-βδ	372.52			4-d-w-u-η-τ	255.51
1-s-r-βδ’	364.31			1-s-w-η-β	191.07
2-d-r-δ	502.85			1-s-w-u-η	192.17
1-d-r-b-δ	512.40			2-s-w-u-η-α	200.07
1-d-r-u-b-δ	509.56			3-d-w-η-τ	185.60
2-d-r-u-δ	500.75			3-d-w-u-η-τ	259.09

1-s-r-u-δ	475.31			2-d-w-η	120.82
2-s-r-u-b-δ	487.84			3-s-w-u-η	191.08
2-r-ξ-βδ	363.28			1-d-w-η′	123.88
1-r-ξ-βδ	352.42			2-d-w-u-η	168.01
				2-s-w-u-η	186.09
				4-d-w-η	126.07
				1-d-w-u-η	168.81
				3-d-w-η	120.75
				4-d-w-u-η	174.80

c) Red-shifts of O12—H17

Average value of the frequency of when not engaged in IHBs (d conformers without O–H17···π interaction): 3765.676 for non-u conformers and 3818.290 cm^{-1} for u conformers.

for the H17···O14 IHB non-u conformers		for the H17···O14 IHB u-conformers		for the H17···π IHB	
conformer	red shift (cm^{-1})	conformer	red shift (cm^{-1})	conformer	red shift (cm^{-1})
3-s-w-η-γτ	873.95	1-s-r-u-αδ	713.12	1-d-r-ξ-αδ	157.04
4-s-w-η-γτ	874.27	2-s-r-u-a-αδ	721.08	2-d-r-ξ-αδ	214.82
4-s-w-a-γτ	861.22	2-s-r-u-b-αδ	712.60	1-d-r-ξ-δ	190.08
3-s-w-a-γτ	859.56	1-s-r-u-a-αδ	716.09	2-d-r-ξ-δ	192.47
3-s-w-η-γ	869.54	4-s-w-u-η-τ	800.95	1-d-w-ξ-α	176.34
4-s-w-η-γ	869.97	3-s-w-u-η-τ	801.32	2-d-w-ξ-α	182.15
4-s-r-b-γε	799.93	1-s-w-u-η-α	787.88	4-d-w-ξ-τ	165.69
3-s-r-γε	805.73	1-s-w-u-η	798.59	3-d-r-ξ-ε	180.82
4-s-r-a-γε	786.74	1-s-r-u-δ	720.94	4-d-r-ξ-ε	184.44
3-s-r-b-γε	797.58	2-s-r-u-b-δ	719.95	3-d-w-ξ-τ	156.57
2-s-r-a-βδ	768.57	2-s-w-u-η-α	782.37	4-w-ξ-yτ	108.36
1-s-r-βδ	760.02	3-s-w-u-a-τ	781.41	3-w-ξ-γτ	118.61
2-s-r-b-βδ	757.82	1-s-w-u-a-α	775.55	1-d-w-ξ	149.56
1-s-r-βδ′	765.11	2-s-w-u-a-α	763.53	2-d-w-ξ	154.22
4-s-w-η-γ ′	870.93	3-s-w-u-η	800.94	4-d-w-ξ	144.46
3-s-w-γ	856.19	2-s-w-u-η	794.92	3-d-w-ξ	138.62
4-s-w-γ	846.24	3-s-w-u	908.95	2-r-ξ-βδ	152.78
2-s-w-η-β	831.31	1-s-w-u	790.24	1-r-ξ-βδ	159.48
1-s-w-η-β	830.37			4-r-ξ-γε	129.56
2-s-w-a-β	816.53			3-r-ξ-γ-ε	136.09
1-s-w-β	821.51				

d) Red-shifts of O26—H27

Average value of the frequency of O26—H27 when not engaged in IHBs: 3776.882 cm^{-1}

for the H27···O8 IHB		for the H27···O10 IHB	
conformer	red shift (cm^{-1})	conformer	red shift (cm^{-1})
1-d-r-ξ-αδ	430.19	3-s-w-η-γτ	372.13
2-d-r-ξ-αδ	427.82	4-s-w-η-γτ	285.62
2-d-r-αδ	421.84	4-s-w-a-γτ	293.97
1-d-r-b-αδ	420.32	3-s-w-a-γτ	285.37
2-d-r-u-αδ	398.35	4-s-w-u-η-τ	254.07
1-d-r-u-b-αδ	400.96	3-s-w-u-η-τ	225.32
1-s-r-u-αδ	288.66	4-d-w-η-τ	185.01
2-s-r-u-a-αδ	285.45	4-d-w-ξ-τ	192.64
2-s-r-u-b-αδ	287.23	4-d-w-u-η-τ	205.18
1-s-r-u-a-αδ	289.47	3-d-w-η-τ	192.66
1-d-w-ξ-α	338.47	3-s-w-u-a-τ	204.48
2-d-w-ξ-α	343.12	3-d-w-u-η-τ	206.84
1-d-w-η-α	348.87	3-d-w-ξ-τ	128.32
2-d-w-η-α	329.92	4-d-w-u-τ	186.73
1-d-w-u-η-α	323.00	4-w-ξ-γτ	304.36
2-d-w-u-η-α	309.36	3-w-ξ-γτ	301.37
1-s-w-u-η-α	212.95	3-d-w-u-a-τ	137.49
2-s-w-u-η-α	165.24		
1-s-w-u-a-α	217.68		
2-s-w-u-a-α	159.67		

Table S 9
Relative energies (ΔE) corrected for ZPE, and ZPE corrections, for the conformers of arzanol in vacuo.
Values from DFT/B3LYP/6-31+G(d,p) frequency calculations.

conformer	ΔE corrected for ZPE (kcal/mol)	ZPE correction (kcal/mol)	conformer	ΔE corrected for ZPE (kcal/mol)	ZPE correction (kcal/mol)
1-d-r-ξ-αδ	0.0000	280.2894	4-d-w-u-η-τ	17.7296	280.3478
2-d-r-ξ-αδ	0.6099	280.8944	1-s-w-η-β	17.5797	280.1188
3-s-w-η-γτ	2.6807	280.9954	1-s-r-u-δ	18.0779	280.4802
4-s-w-η-γτ	2.8131	281.0079	2-s-r-u-b-δ	18.1061	280.3792
2-d-r-αδ	2.8332	280.6484	2-s-w-u-η-α	18.3245	280.2989
1-d-r-b-αδ	2.9022	280.6898	3-d-r-ξ-ε	18.3095	280.2399
4-s-w-a-γτ	5.8471	280.8780	3-d-w-η-τ	18.5121	280.1376
3-s-w-a-γτ	5.8151	280.8071	3-s-w-u-a-τ	18.8918	280.3309
2-d-r-u-αδ	6.3931	280.6710	1-s-w-u-a-α	18.9815	280.3566
1-d-r-u-b-αδ	6.4238	280.6929	4-d-r-ξ-ε	18.7086	280.0491
1-s-r-u-αδ	9.8588	280.9653	3-d-w-u-η-τ	18.9483	280.2832
2-s-r-u-a-αδ	9.7245	280.7990	3-d-w-ξ-τ	19.0355	280.0416
2-s-r-u-b-αδ	9.7822	280.8473	2-s-w-a-β	19.6222	280.1269
1-s-r-u-a-αδ	9.9190	280.9025	1-s-w-β	19.8261	279.8935
1-d-r-ξ-δ	11.4313	280.5474	2-s-w-u-a-α	20.3482	280.0704
2-d-r-ξ-δ	11.4357	280.4877	4-d-w-u-τ	20.6670	280.3616
3-s-w-η-γ	11.7752	280.4695	4-w-ξ-yτ	20.3438	279.9506
4-s-w-η-γ	12.0871	280.5084	3-w-ξ-γτ	20.4173	279.9726
4-s-r-b-γε	12.4943	280.7996	3-d-r-b-ε	20.6607	280.2160
3-s-r-γε	12.5364	280.7946	4-d-r-a-ε	20.9475	279.8797
4-s-r-a-γε	12.5182	280.7406	3-d-w-u-a-τ	21.7714	280.0378
3-s-r-b-γε	12.5376	280.7525	2-d-w-η	21.7972	279.7912
2-s-r-a-βδ	13.2988	280.7971	1-d-w-u-η	22.3067	279.8734
1-s-r-βδ	13.3615	280.8166	1-d-w-ξ	22.1291	279.6783
2-s-r-b-βδ	13.4048	280.8285	2-d-w-ξ	22.3142	279.7172
1-s-r-βδ′	13.4306	280.7306	3-s-w-u-η	22.4222	279.7918
4-s-w-η-γ ′	13.6496	280.3453	1-s-w-u-η	22.7133	279.9481
2-d-r-δ	13.6351	280.3221	3-d-r-u-b-ε	23.0365	280.2066
1-d-r-b-δ	13.7694	280.4068	1-d-w-η′	22.6211	279.7617
1-d-w-ξ-α	13.7211	280.2637	2-d-w-u-η	22.9380	279.9387
2-d-w-ξ-α	13.7996	280.2643	2-s-w-u-η	22.8156	279.7266
1-d-w-η-α	13.9282	280.2411	4-d-w-η	22.9963	279.6877
3-s-w-γ	14.1133	280.4131	4-d-w-ξ	23.0729	279.7435
2-d-w-η-α	13.9181	280.2104	3-d-w-ξ	23.1701	279.5753
4-s-w-γ	14.0725	280.2901	1-d-w-u-η′	23.5328	279.8671
1-d-w-u-η-α	15.4041	280.4639	4-d-r-u-ε	23.5529	279.8546

2-d-w-u-η-α	15.3859	280.4118	3-d-w-η	23.3433	279.6287
4-s-w-u-η-τ	15.9431	280.6032	2-r-ξ-βδ	23.8736	279.9613
3-s-w-u-η-τ	15.9092	280.4520	4-d-w-u-η	23.7813	279.7868
1-s-w-u-η-α	16.6510	280.5536	1-r-ξ-βδ	24.7897	279.9857
1-d-r-u-b-δ	16.9892	280.3930	1-s-w-u	24.9598	279.6425
2-d-r-u-δ	16.8310	280.2336	4-r-ξ-γε	25.1769	279.9550
4-d-w-η-τ	17.2402	280.2625	3-r-ξ-γε	25.2566	279.9531
4-d-w-ξ-τ	17.6286	280.4513	1-d-w-u	25.4706	279.6513
2-s-w-η-β	17.5571	280.3685			

Table S 10

Relative Gibbs free energies (ΔG, sum of electronic and thermal free energies) and thermal corrections to the Gibbs free energy, for the calculated conformers of arzanol in vacuo.

Values from DFT/B3LYP/6-31+G(d,p) frequency calculations.

conformer	sum of electronic and thermal free energies (kcal/mol)	thermal correction to free energy (kcal/mol)	conformer	sum of electronic and thermal free energies for (kcal/mol)	thermal correction to free energy (kcal/mol)
1-d-r-ξ-αδ	0.0000	242.6006	4-d-w-u-η-τ	16.0253	240.9547
2-d-r-ξ-αδ	0.5585	243.1541	1-s-w-η-β	15.9996	240.8505
3-s-w-η-γτ	2.1574	242.7832	1-s-r-u-δ	16.5989	241.3117
4-s-w-η-γτ	2.0871	242.5931	2-s-r-u-b-δ	16.2901	240.8743
2-d-r-αδ	1.8512	241.9775	2-s-w-u-η-α	16.3786	240.6635
1-d-r-b-αδ	1.9804	242.0792	3-d-r-ξ-ε	16.8643	241.1053
4-s-w-a-γτ	5.0797	242.4212	3-d-w-η-τ	16.8775	240.8141
3-s-w-a-γτ	4.7747	242.0779	3-s-w-u-a-τ	16.5625	240.3121
2-d-r-u-αδ	5.0684	241.6569	1-s-w-u-a-α	17.0149	240.7012
1-d-r-u-b-αδ	5.3690	241.9493	4-d-r-ξ-ε	17.0720	240.7237
1-s-r-u-αδ	8.7845	242.2022	3-d-w-u-η-τ	17.2151	240.8612
2-s-r-u-a-αδ	7.9261	241.3111	3-d-w-ξ-τ	17.3412	240.6585
2-s-r-u-b-αδ	8.2768	241.6537	2-s-w-a-β	17.6123	240.4721
1-s-r-u-a-αδ	8.6916	241.9857	1-s-w-β	17.7284	240.1069
1-d-r-ξ-δ	10.4323	241.8595	2-s-w-u-a-α	17.8332	239.8666
2-d-r-ξ-δ	10.3338	241.6970	4-d-w-u-τ	18.4676	240.4734
3-s-w-η-γ	12.7271	241.3381	4-w-ξ-yτ	18.5736	240.4922
4-s-w-η-γ	11.0253	241.7579	3-w-ξ-γτ	18.5787	240.4451
4-s-r-b-γε	11.1057	241.7215	3-d-r-b-ε	19.0142	240.8800
3-s-r-γε	11.4251	241.9945	4-d-r-ε	18.6759	239.9187
4-s-r-a-γε	11.0925	241.6255	3-d-w-u-a-τ	19.3417	239.9199
3-s-r-b-γε	11.3303	241.8564	2-d-w-η	19.8092	240.1144
2-s-r-a-βδ	11.9497	241.7591	1-d-w-η	20.3922	240.2701
1-s-r-βδ	12.0877	241.8539	1-d-w-ξ	20.0633	239.9237
2-s-r-b-βδ	12.2672	242.0020	2-d-w-ξ	20.2271	239.9412
1-s-r-βδ′	11.8919	241.5025	3-s-w-u-η	20.1895	239.8703
4-s-w-η-γ ′	12.2559	241.2634	1-s-w-u-η	20.7442	240.2901
2-d-r-δ	11.7884	240.7865	3-d-r-u-b-ε	20.9852	240.4665
1-d-r-b-δ	12.2753	241.2245	1-d-w-η′	20.5886	240.0410
1-d-w-ξ-α	12.4253	241.2791	2-d-w-u-η	20.9130	240.2249
2-d-w-ξ-α	12.3136	241.0890	2-s-w-u-η	20.0659	239.2880
1-d-w-η-α	12.6794	241.3042	4-d-w-η	20.5603	239.5629
3-s-w-γ	12.7271	241.3381	4-d-w-ξ	21.0397	240.0216
2-d-w-η-α	12.4454	241.0494	3-d-w-ξ	20.6344	239.3508
4-s-w-γ	12.3864	240.9151	1-d-w-u-η′	21.3849	240.0297

1-d-w-u-η-α	14.2049	241.5759	4-d-r-u-ε	21.0040	239.6162
2-d-w-u-η-α	14.1585	241.4950	3-d-w-η	20.8816	239.4775
4-s-w-u-η-τ	14.1585	241.4950	2-r-ξ-βδ	22.2138	240.6133
3-s-w-u-η-τ	14.4666	241.3205	4-d-w-u-η	21.3592	239.6752
1-s-w-u-η-α	15.1989	241.4128	1-r-ξ-βδ	23.2636	240.7708
1-d-r-u-b-δ	15.2071	240.9220	1-s-w-u	21.8994	238.8927
2-d-r-u-δ	14.4685	240.1822	4-r-ξ-γε	23.4601	240.5487
4-d-w-η-τ	14.6203	241.5916	3-r-ξ-γε	23.3728	240.3805
4-d-w-ξ-τ	16.1452	241.2791	1-d-w-u	21.9559	238.4478
2-s-w-η-β	16.2456	241.3688			

Table S 11

HOMO-LUMO energy difference for the calculated conformers of arzanol, *in vacuo*.

HF/6-31G(d,p) and DFT/B3LYP/6-31+G(d,p) results, respectively denoted as HF and DFT in the column headings.

conformer	HOMO-LUMO energy difference (kcal/mol)		conformer	HOMO-LUMO energy difference (kcal/mol)	
	HF	DFT		HF	DFT
1-d-r-ξ-αδ	256.714	101.757	1-s-r-u-δ	244.490	88.705
2-d-r-ξ-αδ	256.614	101.594	2-s-r-u-b-δ	244.245	88.328
3-s-w-η-γτ	251.524	98.055	2-s-w-u-η-α	248.795	93.411
4-s-w-η-γτ	251.437	97.929	3-d-r-ξ-ε	248.500	95.149
2-d-r-αδ	256.475	101.544	3-d-w-η-τ	253.714	98.023
1-d-r-b-αδ	256.080	101.142	3-s-w-u-a-τ	244.170	89.702
4-s-w-a-γτ	251.123	97.691	1-s-w-u-a-α	244.496	88.811
3-s-w-a-γτ	251.041	97.540	4-d-r-ξ-ε	252.390	99.554
2-d-r-u-αδ	248.977	93.806	3-d-w-u-η-τ	247.314	92.043
1-d-r-u-b-αδ	248.663	93.442	3-d-w-ξ-τ	253.639	98.732
1-s-r-u-αδ	249.046	93.455	2-s-w-a-β	246.009	93.938
2-s-r-u-a-αδ	249.083	93.693	1-s-w-β	250.276	97.320
2-s-r-u-b-αδ	248.481	93.210	2-s-w-u-a-α	248.293	93.147
1-s-r-u-a-αδ	248.701	93.649	4-d-w-u-τ	–[a]	87.443
1-d-r-ξ-δ	247.326	92.922	4-w-ξ-yτ	257.122	102.315
2-d-r-ξ-δ	247.170	92.903	3-w-ξ-γτ	255.892	101.399
3-s-w-η-γ	244.697	89.552	4-s-w-u-a-τ	247.941	–[b]
4-s-w-η-γ	244.296	89.251	3-d-r-b-ε	248.908	96.141
4-s-r-b-γε	232.944	78.696	4-d-r-ε	252.723	100.521
3-s-r-γε	232.505	78.470	3-d-w-u-a-τ	247.590	92.445
4-s-r-a-γε	232.260	78.194	2-d-w-η	254.411	101.625
3-s-r-b-γε	232.749	78.533	4-s-r-u-b-ε	245.733	–[b]
2-s-r-a-βδ	231.532	78.206	3-s-r-u-b-ε	245.187	–[b]
1-s-r-βδ	231.595	78.031	1-d-w-η	253.150	100.351
2-s-r-b-βδ	231.563	78.024	1-d-w-ξ	253.137	100.395
1-s-r-βδ′	231.400	77.968	2-d-w-ξ	253.689	100.521
4-s-w-η-γ ′	–[a]	89.288	4-s-w-u-η	249.403	–[b]
2-d-r-δ	247.282	93.668	3-s-w-u-η	249.385	95.576
1-d-r-b-δ	247.013	93.053	1-s-w-u-η	250.665	95.375
1-d-w-ξ-α	246.222	89.514	3-d-r-u-b-ε	248.343	95.030
2-d-w-ξ-α	246.661	89.721	1-d-w-η′	253.783	100.571
1-d-w-η-α	246.442	89.640	2-d-w-u-η	249.598	96.128
3-s-w-γ	244.189	89.414	2-s-w-u-η	249.378	95.934
2-d-w-η-α	249.799	89.301	4-d-w-η	253.846	100.132
4-s-w-γ	244.070	89.527	4-d-w-ξ	253.890	100.395
1-d-w-u-η-α	240.556	84.419	3-d-w-ξ	255.798	101.889

2-d-w-u-η-α	244.107	84.174	1-d-w-u-η′	247.841	94.603
4-s-w-u-η-τ	247.508	89.765	4-d-r-u-ε	248.249	95.300
3-s-w-u-η-τ	243.938	89.652	3-d-w-η	256.162	101.933
1-s-w-u-η-α	243.618	89.138	2-r-ξ-βδ	237.801	83.264
1-d-r-u-b-δ	245.525	91.447	4-d-w-u-η	248.180	94.748
2-d-r-u-δ	245.507	91.987	1-r-ξ-βδ	236.991	82.448
4-d-w-η-τ	248.286	93.129	3-s-w-u	249.385	
4-d-w-ξ-τ	248.638	93.599	1-s-w-u	248.330	94.810
2-s-w-η-β	247.132	94.064	4-r-ξ-γε	238.868	83.013
4-d-w-u-η-τ	242.181	87.437	3-r-ξ-γε	237.481	81.972
1-s-w-η-β	250.671	97.383	1-d-w-u	247.408	94.214

Table S 12

Dipole moments of the calculated conformers of arzanol *in vacuo*.

HF/6-31G(d,p) and DFT/B3LYP/6-31+G(d,p) results, respectively denoted as HF and DFT in the columns' headings.

conformer	dipole moment (debye)		conformer	dipole moment (debye)	
	HF	DFT		HF	DFT
1-d-r-ξ-αδ	1.7108	2.1025	2-s-r-u-b-δ	11.5920	11.8833
2-d-r-ξ-αδ	2.9129	3.0603	2-s-w-u-η-α	3.4050	2.9836
3-s-w-η-γτ	7.2738	7.8278	3-d-r-ξ-ε	9.8493	10.3311
4-s-w-η-γτ	7.6634	8.0550	3-d-w-η-τ	9.8671	10.7005
2-d-r-αδ	2.1080	2.1350	3-s-w-u-a-τ	3.5296	3.7141
1-d-r-b-αδ	2.2600	2.3361	1-s-w-u-a-α	3.8097	4.2539
4-s-w-a-γτ	6.7387	6.9159	4-d-r-ξ-ε	9.7651	10.1112
3-s-w-a-γτ	6.7724	7.0008	3-d-w-u-η-τ	7.1638	7.9406
2-d-r-u-αδ	3.5875	3.7991	3-d-w-ξ-τ	9.9443	10.5139
1-d-r-u-b-αδ	3.9327	3.5714	2-s-w-a-β	8.6189	8.4310
1-s-r-u-αδ	7.9566	8.0117	1-s-w-β	7.7431	7.9090
2-s-r-u-a-αδ	7.7994	7.8135	2-s-w-u-a-α	4.0500	3.9502
2-s-r-u-b-αδ	7.7869	7.8363	4-d-w-u-τ	–[a]	8.5385
1-s-r-u-a-αδ	7.8461	7.8750	4-w-ξ-yτ	9.2131	9.0651
1-d-r-ξ-δ	4.2235	4.5007	3-w-ξ-γτ	10.6631	10.4943
2-d-r-ξ-δ	5.0076	5.0288	4-s-w-u-a-τ	3.3675	–[b]
3-s-w-η-γ	10.9660	11.6523	3-d-r-b-ε	9.9323	10.2885
4-s-w-η-γ	11.2852	11.8949	4-d-r-ε	9.1633	9.4507
4-s-r-b-γε	12.6917	13.2242	3-d-w-u-a-τ	6.7712	7.1098
3-s-r-γε	12.4199	12.7802	2-d-w-η	0.7483	1.5592
4-s-r-a-γε	12.7236	13.0892	4-s-r-u-b-ε	8.5046	–[b]
3-s-r-b-γε	12.8051	13.2462	3-s-r-u-b-ε	8.5322	–[b]
2-s-r-a-βδ	13.3486	13.3906	3-s-r-u-ε	8.2079	–[b]
1-s-r-βδ	13.5859	13.7612	1-d-w-η	7.3426	8.3494
2-s-r-b-βδ	13.2923	13.4686	1-d-w-ξ	7.4757	8.4906
1-s-r-βδ′	13.3053	13.4492	2-d-w-ξ	8.8431	9.4594
4-s-w-η-γ ′	–	11.0979	4-s-w-u-η	7.3230	–[b]
2-d-r-δ	5.0448	5.3734	3-s-w-u-η	–[c]	5.9198
1-d-r-b-δ	5.1367	5.6984	1-s-w-u-η	7.3289	5.7635
1-d-w-ξ-α	6.9361	7.6963	3-d-r-u-b-ε	7.1795	7.0833
2-d-w-ξ-α	7.5064	8.0724	1-d-w-η′	8.5829	9.0338
1-d-w-η-α	6.7927	7.1008	2-d-w-u-η	2.1792	1.5104
3-s-w-γ	10.6327	11.0743	2-s-w-u-η	6.9507	6.6891
2-d-w-η-α	4.6254	7.2181	4-d-w-η	10.0581	10.3488
4-s-w-γ	10.6291	11.0877	4-d-w-ξ	11.1413	11.1192
1-d-w-u-η-α	6.1661	6.3321	3-d-w-ξ	10.4380	10.3359
2-d-w-u-η-α	3.8876	6.4702	1-d-w-u-η′	7.7719	7.7735

4-s-w-u-η-τ	4.0640	4.5612	4-d-r-u-ε	7.1760	7.3951
3-s-w-u-η-τ	3.7958	4.3414	3-d-w-η	10.7380	10.6377
1-s-w-u-η-α	3.0833	2.8830	2-r-ξ-βδ	7.1226	6.8674
1-d-r-u-b-δ	7.0173	6.9740	4-d-w-u-η	8.0997	8.6583
2-d-r-u-δ	6.8221	7.1323	1-r-ξ-βδ	10.0231	9.7534
4-d-w-η-τ	10.5404	11.4429	3-s-w-u	6.7308	–[b]
4-d-w-ξ-τ	10.8449	11.5386	1-s-w-u	6.0670	6.4040
2-s-w-η-β	8.2669	7.9869	4-r-ξ-γε	12.1682	12.3460
4-d-w-u-η-τ	8.2358	9.0342	3-r-ξ-γε	14.2111	14.2080
1-s-w-η-β	7.3270	7.4687	1-d-w-u	7.2873	7.4628
1-s-r-u-δ	11.7303	12.0566			

[a] During HF optimisation of this input, the prenyl rotate to form the O-H···π interaction, yielding the corresponding η conformer

[b] During DFT optimisation of this input, O12H17 rotates 'downwards' yielding the corresponding non-u conformer.

[c] During HF optimisation of this input, O26H27 rotates to the right yielding the corresponding τ conformer.

Table S 13
Relative energies of the conformers of arzanol in the media considered.
DFT/ B3LYP/6-31+G(d,p) results.

a) **Considering results from single-point calculations in solution**
The results in vacuo are from full optimization DFT/ B3LYP /6-31+G(d,p) calculations. The results in solution are from PCM single point calculations on the in-vacuo-optimised geometries.

conformer	relative energy (kcal/mol)			
	vac	chlrf	actn	aq
1-d-r-ξ-αδ	0.0000	0.0011	0.0088	0.0054
2-d-r-ξ-αδ	0.0056	0.0000	0.0000	0.0000
3-s-w-η-γτ	1.9751	1.4733	1.2760	1.0033
4-s-w-η-γτ	2.0946	1.5644	1.3699	1.0176
2-d-r-αδ	2.4743	2.2187	1.9404	0.4011
1-d-r-b-αδ	2.5019	2.3291	2.0467	0.4158
4-s-w-a-γτ	5.2593	4.2920	3.7223	1.5230
3-s-w-a-γτ	5.2980	4.2787	3.6637	1.5291
2-d-r-u-αδ	6.0121	5.5973	5.3867	4.4888
1-d-r-u-b-αδ	6.0206	5.6836	5.4759	4.4493
1-s-r-u-αδ	9.1833	7.9192	7.3048	5.9039
2-s-r-u-a-αδ	9.2157	7.9431	7.3105	5.9548
2-s-r-u-b-αδ	9.2246	8.1235	7.5471	6.0184
1-s-r-u-a-αδ	9.3065	8.0519	7.4200	6.0298
1-d-r-ξ-δ	11.1736	9.0075	8.2110	6.1401
2-d-r-ξ-δ	11.2378	9.0659	8.2937	6.1860
3-s-w-η-γ	11.5954	9.1442	8.1259	5.9618
4-s-w-η-γ	11.8686	9.2773	8.2353	6.0768
4-s-r-b-γε	11.9847	8.6306	7.2408	4.4096
3-s-r-γε	12.0313	8.5555	7.1785	4.5112
4-s-r-a-γε	12.0678	8.3720	7.0470	4.4832
3-s-r-b-γε	12.0749	8.4086	7.0099	4.3826
2-s-r-a-βδ	12.7916	8.8873	7.3621	4.7390
1-s-r-βδ	12.8348	8.8860	7.3395	4.6684
2-s-r-b-βδ	12.8659	9.0889	7.6269	4.7874
1-s-r-βδ′	12.9901	9.0609	7.5163	4.8825
2-d-r-δ	13.6027	11.2591	10.2639	6.6475
1-d-r-b-δ	13.6519	–[a]	10.3322	6.7214
1-d-w-ξ-α	13.7472	10.6357	9.4979	6.2000
2-d-w-ξ-α	13.8252	10.5120	9.4798	6.2571
1-d-w-η-α	13.9766	11.1649	9.8170	6.6495
3-s-w-γ	13.9899	14.7756	9.8855	6.2481

2-d-w-η-α	13.9971	11.1928	–[a]	6.5405
4-s-w-γ	14.0726	11.1865	10.0940	6.5255
1-d-w-u-η-α	15.2303	13.2728	12.4259	10.5482
2-d-w-u-η-α	15.2641	13.2845	12.4552	10.5222
4-s-w-u-η-τ	15.6294	13.8110	13.1656	11.1442
3-s-w-u-η-τ	15.7472	13.9485	13.2780	11.3082
1-s-w-u-η-α	16.3870	14.5044	13.6875	11.5216
1-d-r-u-b-δ	16.8861	14.5403	13.5637	10.5733
2-d-r-u-δ	16.8873	14.4734	13.4538	10.5838
4-d-w-η-τ	17.2674	13.2434	11.4514	8.0370
4-d-w-ξ-τ	17.4668	13.2946	11.5298	7.7809
2-s-w-η-β	17.4782	14.4025	13.1885	10.1780
4-d-w-u-η-τ	17.6715	14.8979	13.7540	11.7751
1-s-w-η-β	17.7503	14.6618	13.3618	10.2440
1-s-r-u-δ	17.8873	14.7756	13.4143	10.3131
2-s-r-u-b-δ	18.0168	15.0573	13.7211	10.5100
2-s-w-u-η-α	18.3159	16.6679	15.8418	13.4009
3-d-r-ξ-ε	18.3595	14.6916	13.1191	10.2846
3-d-w-η-τ	18.6644	14.9399	13.0956	9.5963
3-s-w-u-a-τ	18.8511	16.5997	15.5642	11.8759
1-s-w-u-a-α	18.9152	16.3824	15.2019	11.5797
4-d-r-ξ-ε	18.9490	15.2772	13.6198	10.6306
3-d-w-u-η-τ	18.9549	16.4816	15.3764	13.3144
3-d-w-ξ-τ	19.2834	15.2518	13.4615	9.5356
2-s-w-a-β	19.7416	16.5547	15.1963	10.8637
1-s-w-β	20.2229	16.6488	14.8903	10.4429
2-s-w-u-a-α	20.5679	18.8270	17.7598	13.9144
4-w-ξ-γτ	20.6825	16.5020	14.7010	10.8626
3-w-ξ-γτ	20.7340	16.2652	14.5773	10.5154
3-d-r-b-ε	20.7350	16.8804	14.9249	10.6498
4-d-r-ε	21.3578	17.4158	15.5071	11.0036
1-d-w-η	22.7227	18.4404	16.4676	11.2825
1-d-w-ξ	22.7404	18.1203	16.2228	11.1104
1-s-w-u-η	23.0548	19.9529	18.5253	14.7903
3-d-r-u-b-ε	23.1199	19.6624	18.2920	14.4470
1-d-w-η′	23.1482	18.6269	16.4370	11.1641
3-d-w-ξ	23.8842	19.1479	17.0607	11.8887
4-d-r-u-ε	23.9884	20.3034	18.6783	14.8181
3-d-w-η	24.0045	19.4323	17.2400	11.9308
2-r-ξ-βδ	24.2020	19.4701	17.6329	13.9012
1-r-ξ-βδ	25.0941	19.6639	17.7128	13.8607

[a] The calculation in the given solvent does not converge for this conformer.

b) Comparison of results from full-reoptimisation and single-point calculations in solution

The cases in which full re-optimisation in solution converged for at least one solvent are reported.

conformer						
	full-reoptimisation			single-point calculations		
	chlrf		actn		aq	
	SP	reopt	SP	reopt	SP	reopt
1-d-r-ξ-αδ	0.0011	0.0000	0.0088	0.0000	0.0054	0.0420
2-d-r-ξ-αδ	0.0000		0.0000	0.0017	0.0000	0.0504
4-s-w-η-γτ	1.5644	1.5540	1.3699		1.0176	
2-d-r-αδ	2.2187	2.1970	1.9404	1.7090	0.4011	0.0000
4-s-w-a-γτ	4.2920	4.2484	3.7223	3.5750	1.5230	0.8855
3-s-w-a-γτ	4.2787		3.6637		1.5291	0.9083
2-s-r-u-a-αδ	7.9431		7.3105	6.6406	5.9548	3.2742
2-d-r-ξ-δ	9.0659		8.2937	8.0646	6.1860	5.8043
3-s-w-η-γ	9.1442		8.1259	7.8830	5.9618	
3-s-r-γε	8.5555		7.1785		4.5112	3.8526
4-s-r-a-γε	8.3720	8.2298	7.0470	6.7180	4.4832	3.7339
2-s-r-a-βδ	8.8873	8.7039	7.3621	6.9589	4.7390	3.9018
1-s-r-βδ	8.8860	8.6834	7.3395	6.9524	4.6684	3.8446
1-s-r-βδ′	9.0609		7.5163		4.8825	4.0241
2-d-r-δ	11.2591		10.2639	9.9130	6.6475	5.8752
1-d-w-ξ-α	10.6357	10.6188	9.4979	9.3198	6.2000	5.8646
2-d-w-ξ-α	10.5120		9.4798		6.2571	5.8119
1-d-w-η-α	11.1649	11.0757	9.8170		6.6495	
3-s-w-γ	14.7756		9.8855		6.2481	5.4813
2-d-w-η-α	11.1928		–[a]		6.5405	5.9714
1-s-w-u-η-α	14.5044		13.6875		11.5216	10.5369
4-d-w-η-τ	13.2434	13.0743	11.4514	11.1104	8.0370	7.2247
4-d-w-ξ-τ	13.2946	13.1601	11.5298	11.1175	7.7809	6.9910
2-s-w-η-β	14.4025	14.4062	13.1885	12.9196	10.1780	
3-d-r-ξ-ε	14.6916	14.6063	13.1191		10.2846	9.5892
3-d-w-η-τ	14.9399	14.7486	13.0956	12.7449	9.5963	
1-s-w-u-a-α	16.3824		15.2019		11.5797	10.3008
4-d-r-ξ-ε	15.2772	15.1198	13.6198	13.3009	10.6306	9.3734
3-d-w-ξ-τ	15.2518		13.4615	13.0428	9.5356	
2-s-w-a-β	16.5547		15.1963		10.8637	9.6460
1-s-w-β	16.6488		14.8903		10.4429	9.2482
2-s-w-u-a-α	18.8270		17.7598		13.9144	11.1821
4-w-ξ-γτ	16.5020	16.4358	14.7010	14.3487	10.8626	
3-w-ξ-γτ	16.2652	16.1368	14.5773	14.2725	10.5154	
3-d-r-b-ε	16.8804	16.7206	14.9249	14.7492	10.6498	
4-d-r-ε	17.4158	17.1604	15.5071	14.9369	11.0036	9.2482
2-r-ξ-βδ	19.4701		17.6329	17.2841	13.9012	
1-r-ξ-βδ	19.6639	19.4694	17.7128	17.2563	13.8607	13.1682

Table S 14
Comparison of the parameters of the intramolecular hydrogen bonds in different media for the calculated conformers of arzanol for which full re-optimisation in solution converged. DFT/B3LYP/6-31+G(d,p) results.

conformer	IHB considered	medium	parameters of the IHB		
			H···O (Å)	O···O (Å)	$O\hat{H}O$
1-d-r-ξ-αδ	H15···O14	vac	1.482	2.441	152.8
		chlrf	1.486	2.444	152.8
		actn	1.488	2.444	152.8
		aq	1.499	2.449	152.3
	H27···O8	vac	1.760	2.740	170.8
		chlrf	1.762	2.742	170.5
		actn	1.763	2.742	170.3
		aq	1.766	2.743	169.1
	H16···O23	vac	1.697	2.683	170.2
		chlrf	1.695	2.681	169.7
		actn	1.696	2.680	169.4
		aq	1.699	2.681	168.3
2-d-r-ξ-αδ	H15···O14	vac	1.482	2.441	152.8
		actn	1.487	2.444	152.9
		aq	1.498	2.449	152.4
	H27···O8	vac	1.764	2.743	170.6
		actn	1.768	2.746	170.1
		aq	1.773	2.749	169.0
	H16···O23	vac	1.701	2.687	169.9
		actn	1.701	2.685	169.3
		aq	1.704	2.686	169.2
3-s-w-η-γτ	H17···O14	vac	1.528	2.465	151.6
		actn	1.656	2.513	145.6
		aq	1.662	2.517	146.2
	H15···O23	vac	1.697	2.682	170.5
		actn	1.784	2.734	170.9
		aq	1.786	2.733	169.5
	H27···O10	vac	1.797	2.769	169.4
		actn	1.912	2.835	162.7
		aq	1.939	2.841	157.2
4-s-w-η-γτ	H17···O14	vac	1.528	2.466	151.6
		chlrf	1.528	2.467	152.1
	H15···O23	vac	1.700	2.684	170.3
		chlrf	1.688	2.674	170.1
	H27···O10	vac	1.794	2.766	168.9
		chlrf	1.801	2.772	168.4
4-s-w-a-γτ	H17···O14	vac	1.533	2.468	151.4
		chlrf	1.535	2.471	151.8

		actn	1.536	2.472	152.0
		aq	1.537	2.473	152.0
	H15···O23	vac	1.691	2.677	170.6
		chlrf	1.680	2.667	170.4
		actn	1.675	2.662	170.2
		aq	1.680	2.665	169.1
	H27···O10	vac	1.820	2.790	169.3
		chlrf	1.819	2.789	168.7
		actn	1.819	2.788	168.3
		aq	1.805	2.773	167.1
2-d-r-αδ	H17···O14	vac	1.483	2.441	152.8
		chlrf	1.485	2.443	152.8
		actn	1.485	2.442	152.9
		aq	1.492	2.445	152.5
	H27···O8	vac	1.765	2.744	170.4
		chlrf	1.766	2.744	170.1
		actn	1.768	2.746	170.0
		aq	1.770	2.746	168.9
	H16···O23	vac	1.701	2.686	169.7
		chlrf	1.707	2.691	169.3
		actn	1.709	2.692	169.2
		aq	1.715	2.694	167.9
3-s-w-a-γτ	H17···O14	vac	1.533	2.468	151.4
		aq	1.537	2.473	152.0
	H15···O23	vac	1.690	2.675	170.5
		aq	1.679	2.664	169.1
	H27···O10	vac	1.822	2.791	169.1
		aq	1.806	2.771	166.5
2-s-r-u-a-αδ	H17···O14	vac	1.576	2.493	150.1
		actn	1.564	2.490	151.0
		aq	1.561	2.488	151.1
	H27···O8	vac	1.814	2.785	170.2
		actn	1.795	2.763	167.1
		aq	1.765	2.735	166.7
	H16···O23	vac	1.702	2.686	170.4
		actn	1.688	2.674	170.4
		aq	1.700	2.683	168.8
2-d-r-ξ-δ	H15···O14	vac	1.543	2.477	151.7
		actn	1.541	2.477	152.3
		aq	1.540	2.476	152.2
	H16···O23	vac	1.685	2.658	166.1
		actn	1.635	2.618	167.8
		aq	1.624	2.608	167.2
3-s-w-η-γ	H17···O14	vac	1.538	2.471	151.5
		actn	1.527	2.468	152.6
		aq	1.527	2.468	152.5
	H15···O23	vac	1.937	2.868	159.9
		actn	1.932	2.617	168.4

		aq	1.922	2.607	167.8
3-s-r-γε	H17···O14	vac	1.545	2.477	151.3
		aq	1.540	2.475	152.1
	H15···O23	vac	1.732	2.710	170.9
		aq	1.777	2.761	169.5
	H16···O26	vac	1.958	2.908	166.1
		aq	1.898	2.856	166.7
4-s-r-a-γε	H17···O14	vac	1.545	2.477	151.4
		chlrf	1.542	2.476	151.9
		actn	1.540	2.476	152.2
		aq	1.539	2.475	152.2
	H15···O23	vac	1.734	2.712	171.0
		chlrf	1.696	2.679	171.2
		actn	1.683	2.668	171.0
		aq	1.674	2.659	169.8
	H16···O26	vac	1.954	2.908	167.8
		chlrf	1.926	2.887	169.4
		actn	1.916	2.878	169.6
		aq	1.894	2.855	168.0
3-s-r-b-γε	H17···O14	vac	1.548	2.478	151.1
		actn	1.669	2.523	146.3
		aq	1.675	2.527	145.9
	H15···O23	vac	1.733	2.711	170.9
		actn	1.790	2.737	169.8
		aq	1.786	2.732	169.1
	H16···O26	vac	1.952	2.904	166.8
		actn	1.994	2.923	167.0
		aq	1.978	2.903	165.1
2-s-r-a-βδ	H17···O14	vac	1.553	2.481	151.1
		chlrf	1.545	2.478	151.8
		actn	1.541	2.476	152.1
		aq	1.540	2.476	152.2
	H15···O26	vac	1.939	2.892	166.9
		chlrf	1.917	2.877	168.9
		actn	1.905	2.867	169.4
		aq	1.882	2.842	167.9
	H16···O23	vac	1.752	2.727	170.3
		chlrf	1.712	2.692	170.3
		actn	1.699	2.680	170.0
		aq	1.689	2.670	168.9
1-s-r-βδ	H17···O14	vac	1.557	2.483	150.9
		chlrf	1.545	2.478	150.9
		actn	1.541	2.476	152.1
		aq	1.539	2.475	152.1
	H15···O26	vac	1.939	2.891	166.8
		chlrf	1.913	2.873	166.8
		actn	1.902	2.864	169.2
		aq	1.881	2.842	167.7

	H16···O23	vac	1.751	2.726	170.2
		chlrf	1.713	2.692	170.2
		actn	1.701	2.681	169.7
		aq	1.691	2.671	168.5
1-s-r-βδ′	H17···O14	vac	1.553	2.481	151.1
		aq	1.541	2.476	152.1
	H15···O26	vac	1.937	2.891	167.5
		aq	1.880	2.842	168.4
	H16···O23	vac	1.752	2.726	169.7
		aq	1.887	2.667	168.6
2-d-r-δ	H15···O14	vac	1.543	2.477	151.7
		actn	1.538	2.475	152.3
		aq	1.533	2.472	152.4
	H16···O23	vac	1.687	2.667	165.7
		actn	1.640	2.621	167.3
		aq	1.628	2.610	166.9
1-d-w-ξ-α	H15···O14	vac	1.507	2.454	152.0
		chlrf	1.499	2.450	152.4
		actn	1.496	2.449	152.6
		aq	1.502	2.452	152.3
	H27···O8	vac	1.769	2.740	169.2
		chlrf	1.747	2.721	169.5
		actn	1.739	2.714	169.5
		aq	1.730	2.705	168.9
2-d-w-ξ-α	H15···O14	vac	1.506	2.453	152.8
		aq	1.501	2.452	152.4
	H27···O8	vac	1.766	2.737	169.0
		aq	1.731	2.705	168.6
1-d-w-η-α	H15···O14	vac	1.498	2.448	152.1
		chlrf	1.491	2.444	152.6
	H27···O8	vac	1.761	2.732	168.7
		chlrf	1.746	2.719	169.1
3-s-w-γ	H17···O14	vac	1.535	2.471	151.6
		aq	1.536	2.473	152.2
	H15···O23	vac	1.676	2.653	167.7
		aq	1.627	2.612	167.8
2-d-w-η-α	H15···O14	vac	1.501	2.449	152.0
		aq	1.489	2.443	152.6
	H27···O8	vac	1.776	2.747	168.1
		aq	1.732	2.707	168.7
1-s-w-u-η-α	H17···O14	vac	1.557	2.480	150.5
		aq	1.571	2.493	150.4
	H27···O8	vac	1.842	2.806	168.6
		aq	1.835	2.793	164.4
4-d-w-η-τ	H15···O14	vac	1.564	2.483	149.7
		chlrf	1.549	2.477	150.9
		actn	1.541	2.474	151.5
		aq	1.536	2.471	151.8

	H27···O10	vac	1.842	2.804	168.0
		chlrf	1.809	2.775	168.8
		actn	1.793	2.761	170.0
		aq	1.777	2.743	167.6
4-d-w-ξ-τ	H15···O14	vac	1.571	2.489	149.7
		chlrf	1.557	2.483	150.8
		actn	1.549	2.480	151.4
		aq	1.548	2.480	151.6
	H27···O10	vac	1.857	2.818	167.8
		chlrf	1.823	2.788	168.7
		actn	1.806	2.774	168.9
		aq	1.778	2.747	168.1
2-s-w-η-β	H17···O14	vac	1.538	2.471	151.5
		chlrf	1.530	2.468	152.2
		actn	1.527	2.467	152.5
	H15···O26	vac	1.937	2.868	159.9
		chlrf	1.907	2.846	161.5
		actn	1.897	2.837	161.7
3-d-r-ξ-ε	H15···O14	vac	1.547	2.477	151.1
		chlrf	1.538	2.474	152.0
		aq	1.532	2.471	152.5
	H16···O26	vac	1.926	2.865	162.1
		chlrf	1.895	2.840	163.1
		aq	1.868	2.810	161.5
3-d-w-η-τ	H15···O14	vac	1.555	2.478	150.3
		chlrf	1.541	2.472	151.5
		actn	1.534	2.469	152.0
	H27···O10	vac	1.852	2.752	151.8
		chlrf	1.816	2.727	153.6
		actn	1.807	2.720	153.8
1-s-w-u-a-α	H17···O14	vac	1.562	2.483	150.3
		aq	1.583	2.502	150.0
	H27···O8	vac	1.837	2.802	168.7
		aq	1.840	2.798	164.7
4-d-r-ξ-ε	H15···O14	vac	1.562	2.485	150.3
		chlrf	1.553	2.482	151.2
		actn	1.547	2.480	151.8
		aq	1.543	2.478	152.0
	H16···O26	vac	1.963	2.830	147.4
		chlrf	1.941	2.812	147.7
		actn	1.936	2.804	147.2
		aq	2.727	3.119	104.6
3-d-w-ξ-τ	H15···O14	vac	1.560	2.483	150.4
		actn	1.541	2.476	152.0
	H27···O10	vac	1.931	2.787	145.4
		actn	1.883	2.748	146.2
2-s-w-a-β	H17···O14	vac	1.543	2.475	151.3
		aq	1.535	2.472	152.2

	H15···O26	vac	1.947	2.876	159.4
		aq	1.866	2.803	160.5
1-s-w-β	H17···O14	vac	1.542	2.474	151.3
		aq	1.535	2.471	152.1
	H15···O26	vac	1.934	2.828	151.9
		aq	2.586	3.051	109.4
2-s-w-u-a-α	H17···O14	vac	1.565	2.485	150.2
		aq	1.564	2.489	150.9
	H27···O8	vac	1.885	2.771	149.7
		aq	2.120	2.812	127.4
4-w-ξ-γτ	H15···O23	vac	1.720	2.705	172.6
		chlrf	1.707	2.693	171.9
		actn	1.701	2.687	171.4
	H27···O10	vac	1.805	2.778	170.4
		chlrf	1.796	2.770	170.2
		actn	1.793	2.767	169.9
3-w-ξ-γτ	H15···O23	vac	1.733	2.715	171.0
		chlrf	1.716	2.701	171.9
		actn	1.709	2.695	172.1
	H27···O10	vac	1.810	2.782	169.8
		chlrf	1.804	2.776	169.6
		actn	1.802	2.774	169.4
3-d-r-b-ε	H15···O14	vac	1.546	2.476	151.1
		chlrf	1.535	2.472	152.0
		actn	1.530	2.469	152.5
	H16···O26	vac	1.924	2.875	162.3
		chlrf	1.903	2.848	163.3
		actn	1.892	2.838	163.4
4-d-r-ε	H15···O14	vac	1.561	2.485	153.3
		chlrf	1.550	2.480	151.3
		actn	1.543	2.477	151.8
		aq	1.536	2.472	152.1
	H16···O26	vac	1.981	2.841	146.4
		chlrf	1.958	2.821	146.6
		actn	1.968	2.821	145.1
		aq	2.657	3.075	106.2
2-r-ξ-βδ	H15···O26	vac	1.937	2.895	169.8
		actn	1.928	2.893	171.5
	H16···O23	vac	1.752	2.729	171.7
		actn	1.705	2.688	172.0
1-r-ξ-βδ	H15···O26	vac	1.969	2.921	167.0
		chlrf	1.934	2.897	170.7
		actn	1.919	2.885	171.9
		aq	1.892	2.858	170.6
	H16···O23	vac	1.760	2.735	171.1
		chlrf	1.720	2.701	171.6
		actn	1.707	2.690	171.5
		aq	1.697	2.679	170.2

Table S 15

Comparison of the distance between the H atom of the phenol OH engaged in the O–H···π interaction with the C29=C30 double bond in the prenyl chain and the two C atoms forming the double bond in different media, for the calculated conformers of arzanol for which full re-optimisation in solution converged. DFT/B3LYP/6-31+G(d,p) results.

The H atom is H16 for η-conformers and H17 for ξ-conformers.

conformer	medium	H16···C29 (η) or H17···C29 (ξ) (Å)	H16···C30 (η) or H17···C30 (ξ) (Å)
1-d-r-ξ-αδ	vacuum	2.104	2.477
	chloroform	2.079	2.459
	acetonitrile	2.071	2.456
	water	2.054	2.448
2-d-r-ξ-αδ	vacuum	2.086	2.460
	acetonitrile	2.061	2.438
	water	2.050	2.436
2-d-r-ξ-δ	vacuum	2.100	2.488
	acetonitrile	2.072	2.464
	water	2.060	2.455
1-d-w-ξ-α	vacuum	2.130	2.510
	chloroform	2.082	2.480
	acetonitrile	2.064	2.462
	water	2.067	2.467
2-d-w-ξ-α	vacuum	2.116	2.508
	water	2.055	2.472
4-d-w-ξ-τ	vacuum	2.132	2.525
	chloroform	2.081	2.488
	acetonitrile	2.062	2.473
	water	2.055	2.468
3-d-r-ξ-ε	vacuum	2.111	2.491
	chloroform	2.085	2.475
	water	2.057	2.458
4-d-r-ξ-ε	vacuum	2.105	2.500
	chloroform	2.079	2.479
	acetonitrile	2.071	2.467
	water	2.056	2.449
3-d-w-ξ-τ	vacuum	2.132	2.525
	acetonitrile	2.073	2.475
4-w-ξ-γτ	vacuum	2.220	2.556
	chloroform	2.147	2.514
	acetonitrile	2.127	2.501
3-w-ξ-γτ	vacuum	2.185	2.538
	chloroform	2.131	2.486
	acetonitrile	2.116	2.486
2-r-ξ-βδ	vacuum	2.216	2.507
	acetonitrile	2.133	2.482

1-r-ξ-βδ	vacuum	2.143	2.495
	chloroform	2.111	2.474
	acetonitrile	2.103	2.468
3-s-w-η-γτ	vacuum	2.071	2.426
	acetonitrile	2.245	2.572
	water	2.270	2.611
4-s-w-η-γτ	vacuum	2.054	2.429
	chloroform	2.033	2.413
3-s-w-η-γ	vacuum	2.138	2.502
	acetonitrile	2.110	2.487
	water	2.088	2.464
1-d-w-η-α	vacuum	2.152	2.509
	chloroform	2.112	2.499
2-d-w-η-α	vacuum	2.157	2.513
	water	2.067	2.465
1-s-w-u-η-α	vacuum	2.112	2.469
	water	2.081	2.469
4-d-w-η-τ	vacuum	2.110	2.471
	chloroform	2.370	2.424
	acetonitrile	2.019	2.411
	water	2.033	2.430
2-s-w-η-β	vacuum	2.126	2.492
	chloroform	2.107	2.499
	acetonitrile	2.104	2.507
3-d-w-η-τ	vacuum	2.122	2.472
	chloroform	2.055	2.431
	acetonitrile	2.037	2.422

Table S 16
Solvent effect (free energy of solvation, ΔG_{solv}) and its electrostatic component (G_{el}) for the conformers of arzanol in the three solvents considered.

a) Results from single point DFT/B3LYP/6-31+G(d,p) calculations in solution on the in-vacuo-optimised conformers

conformer	ΔG_{solv}			G_{el}		
	in chloroform	in acetonitrile	in water	in chloroform	in acetonitrile	in water
1-d-r-ξ-αδ	1.49	8.05	-2.90	-3.81	-5.30	-11.80
2-d-r-ξ-αδ	1.61	8.12	-2.82	-3.82	-5.31	-11.81
3-s-w-η-γτ	1.37	7.65	-3.51	-4.32	-6.00	-12.78
4-s-w-η-γτ	1.55	7.83	-3.33	-4.34	-6.03	-12.88
2-d-r-αδ	1.67	7.98	-4.42	-4.07	-5.84	-13.88
1-d-r-b-αδ	1.99	8.23	-4.20	-3.99	-5.76	-13.89
4-s-w-a-γτ	0.80	6.86	-6.21	-4.78	-6.84	-15.54
3-s-w-a-γτ	1.14	7.04	-5.93	-4.83	-6.94	-15.58
2-d-r-u-αδ	1.56	7.93	-3.85	-4.23	-5.93	-13.33
1-d-r-u-b-αδ	1.86	8.17	-3.67	-4.15	-5.85	-13.38
1-s-r-u-αδ	0.71	6.68	-5.60	-5.08	-7.18	-15.09
2-s-r-u-a-αδ	0.64	6.60	-5.63	-5.09	-7.21	-15.07
2-s-r-u-b-αδ	1.40	7.29	-5.03	-4.92	-6.98	-15.01
1-s-r-u-a-αδ	0.87	6.77	-5.48	-5.07	-7.19	-15.08
1-d-r-ξ-δ	-0.27	5.55	-7.41	-5.98	-8.27	-16.84
2-d-r-ξ-δ	-0.13	5.64	-7.35	-5.99	-8.25	-16.86
3-s-w-η-γ	-0.19	5.29	-7.73	-6.27	-8.77	-17.44
4-s-w-η-γ	-0.18	5.24	-7.77	-6.41	-8.94	-17.60
4-s-r-b-γε	-0.85	4.25	-9.40	-7.17	-10.05	-19.38
3-s-r-γε	-1.14	4.00	-9.50	-7.29	-10.16	-19.33
4-s-r-a-γε	-1.76	3.54	-9.88	-7.51	-10.33	-19.39
3-s-r-b-γε	-1.52	3.66	-9.81	-7.48	-10.37	-19.50
2-s-r-a-βδ	-1.90	3.18	-10.31	-7.72	-10.73	-19.86
1-s-r-βδ	-1.94	3.11	-10.42	-7.76	-10.80	-19.97
2-s-r-b-βδ	-1.27	3.77	-9.84	-7.59	-10.54	-19.88
1-s-r-βδ′	-1.74	3.27	-10.20	-7.74	-10.78	-19.91
2-d-r-δ	-0.13	5.50	-8.94	-6.16	-8.64	-18.76
1-d-r-b-δ	–[a]	5.79	-8.55	–[a]	-8.62	-18.74
1-d-w-ξ-α	-0.94	4.55	-9.54	-6.93	-9.55	-19.35
2-d-w-ξ-α	-0.98	4.55	-9.47	-7.13	-9.65	-19.37
1-d-w-η-α	-0.18	4.97	-8.96	-6.63	-9.46	-19.13
3-s-w-γ		4.89	-9.56		-9.41	-19.55

2-d-w-η-α	-0.36	–[a]	-9.27	-6.62	–[a]	-19.26
4-s-w-γ	-0.80	4.78	-9.62	-6.70	-9.28	-19.35
1-d-w-u-η-α	0.74	6.38	-6.28	-5.77	-8.11	-16.49
2-d-w-u-η-α	0.57	6.25	-6.50	-5.79	-8.11	-16.55
4-s-w-u-η-τ	0.97	6.87	-5.83	-5.63	-7.77	-16.29
3-s-w-u-η-τ	0.83	6.67	-6.06	-5.61	-7.77	-16.25
1-s-w-u-η-α	0.75	6.47	-6.47	-5.70	-8.00	-16.67
1-d-r-u-b-δ	0.14	5.76	-7.98	-6.16	-8.63	-18.12
2-d-r-u-δ	-0.21	5.38	-8.34	-6.23	-8.74	-18.11
4-d-w-η-τ	-1.56	3.24	-10.92	-7.84	-11.12	-21.04
4-d-w-ξ-τ	-1.66	3.16	-11.34	-7.99	-11.24	-21.49
2-s-w-η-β	-0.40	4.92	-8.85	-6.89	-9.59	-19.11
4-d-w-u-η-τ	-0.25	5.19	-7.56	-6.59	-9.22	-17.70
1-s-w-η-β	-0.54	4.70	-9.21	-6.90	-9.69	-19.31
1-s-r-u-δ	-0.91	4.36	-9.59	-6.93	-9.78	-19.38
2-s-r-u-b-δ	-0.29	4.91	-9.06	-6.77	-9.60	-19.31
2-s-w-u-η-α	0.75	6.48	-6.77	-5.46	-7.78	-16.72
3-d-r-ξ-ε	-1.17	3.84	-9.78	-7.48	-10.55	-19.88
3-d-w-η-τ	-0.59	4.05	-10.05	-7.54	-10.87	-20.87
3-s-w-u-a-τ	0.87	6.38	-7.99	-6.07	-8.59	-18.78
1-s-w-u-a-α	0.37	5.76	-8.57	-6.35	-9.02	-19.14
4-d-r-ξ-ε	-1.34	3.59	-10.22	-7.49	-10.63	-20.12
3-d-w-u-η-τ	0.65	6.02	-6.67	-6.29	-8.88	-17.45
3-d-w-ξ-τ	-1.82	3.01	-11.73	-7.85	-11.13	-21.55
2-s-w-a-β	-0.06	5.14	-9.84	-7.00	-9.85	-20.68
1-s-w-β	-0.82	4.03	-11.16	-7.39	-10.64	-21.59
2-s-w-u-a-α	1.07	6.58	-7.98	-5.56	-8.11	-18.46
4-w-ξ-yτ	-1.68	3.19	-11.35	-8.00	-11.29	-21.63
3-w-ξ-γτ	-1.67	3.25	-11.48	-8.28	-11.46	-22.03
3-d-r-b-ε	-0.14	4.29	-10.65	-7.67	-11.12	-21.89
4-d-r-ε	-1.49	3.28	-12.00	-7.76	-11.16	-22.16
1-d-w-η	-1.27	3.29	-12.58	-8.10	-11.56	-23.25
1-d-w-ξ	-1.94	2.79	-13.02	-8.44	-11.82	-23.44
1-s-w-u-η	0.04	5.10	-9.33	-6.92	-9.83	-20.07
3-d-r-u-b-ε	0.40	5.43	-9.04	-7.27	-10.13	-20.48
1-d-w-η′	-0.96	3.30	-12.59	-8.34	-12.02	-23.80
3-d-w-ξ	-1.97	2.55	-13.31	-8.55	-12.13	-23.79
4-d-r-u-ε	-1.19	3.85	-10.79	-7.50	-10.62	-20.98
3-d-w-η	2.56	3.47	-12.28	-8.39	-12.07	-23.88
2-r-ξ-βδ	-1.89	2.89	-11.53	-8.55	-11.87	-22.11
1-r-ξ-βδ	-2.64	2.04	-12.51	-9.24	-12.69	-23.04

[a] The calculation in the given solvent does not converge for this conformer.

b) Results from DFT/B3LYP/6-31+G(d,p) calculations with full re-optimisation in solution

conformer	ΔG_{solv} (kcal/mol)			G_{el} (kcal/mol)		
	in chloroform	in acetonitrile	in water	in chloroform	in acetonitrile	in water
1-d-r-ξ-αδ	1.33	7.77	-3.63	-3.98	-5.59	-12.59
2-d-r-ξ-αδ		7.86	-3.50		-5.59	-12.57
4-s-w-η-γτ	1.34			-4.54		
2-d-r-αδ		7.32	-6.14		-6.43	-15.57
4-s-w-a-γτ	0.52	6.25	-8.36	-5.06	-7.44	-17.73
3-s-w-a-γτ			-8.13			-17.73
2-s-r-u-a-αδ		4.45	-10.80		-9.40	-20.44
2-d-r-ξ-δ		4.89	-9.09		-8.95	-18.53
3-s-w-η-γ		4.51	-9.47		-9.51	-19.16
3-s-r-γε			-12.07			-21.70
4-s-r-a-γε	-2.34	2.46	-12.47	-7.99	-11.28	-21.82
2-s-r-a-βδ	-2.55	1.95	-13.14	-8.28	-11.84	-22.52
1-s-r-βδ	-2.57	1.96	-13.15	-8.32	-11.85	-22.59
1-s-r-βδ′			-13.04			-22.60
2-d-r-δ		4.52	-11.52		-9.56	-21.30
1-d-w-ξ-α	-1.22	3.88	-11.03	-7.20	-10.20	-20.85
2-d-w-ξ-α			-11.05			-21.01
1-d-w-η-α	-0.58			-7.00		
3-s-w-γ			-12.05			-22.02
2-d-w-η-α			-11.18			-21.17
1-s-w-u-η-α			-10.01			-20.33
4-d-w-η-τ	-2.14	2.14	-13.37	-8.36	-12.13	-23.42
4-d-w-ξ-τ	-2.22	1.97	-13.94	-8.45	-12.31	-23.94
2-s-w-η-β	2.12	3.99		-7.20	-10.45	
3-d-r-ξ-ε	-1.64		-11.67	-7.89		-22.26
3-d-w-η-τ	-1.25	2.96		-8.09	-11.89	
1-s-w-u-a-α			-12.86			-23.63
4-d-r-ξ-ε	-1.87	2.59	-13.92	-7.96	-11.57	-24.29
3-d-w-ξ-τ		1.81			-12.22	
2-s-w-a-β			-13.32			-24.01
1-s-w-β			-15.12			-25.93
2-s-w-u-a-α			-13.77			-24.52
4-w-ξ-γτ	-2.10	2.17		-8.37	-12.22	
3-w-ξ-γτ	-2.13	2.37		-8.71	-12.32	
3-d-r-b-ε	-0.58	3.56		-8.20	-11.98	
4-d-r-ε	-2.12	1.97	-16.57	-8.35	-12.44	-27.06
2-r-ξ-βδ		1.77			-12.89	
1-r-ξ-βδ	-3.28	0.77		-9.81	-13.84	

Table S 17
Dipole moment of the conformers of arzanol in the media considered.
DFT/B3LYP/6-31+G(d,p) results.

a) **Results in vacuo from DFT/B3LYP/6-31+G(d,p) calculations with full optimisation (fully relaxed geometries). Results in solution from single point DFT/B3LYP/6-31+G(d,p) calculations on the in-vacuo-optimised geometries.**

conformer	dipole moment (debye)			
	in vacuo	in chloroform	in acetonitrile	in water
1-d-r-ξ-αδ	2.1025	2.4418	2.5581	2.6539
2-d-r-ξ-αδ	3.0603	3.4770	3.6095	3.5706
3-s-w-η-γτ	7.8278	9.4420	10.0828	10.8118
4-s-w-η-γτ	8.0550	9.6686	10.2921	10.9545
2-d-r-αδ	2.1350	2.4976	2.6763	2.8685
1-d-r-b-αδ	2.3361	2.6230	2.6943	2.6820
4-s-w-a-γτ	6.9159	8.4843	9.1938	10.1526
3-s-w-a-γτ	7.0008	8.5418	9.2283	10.1309
2-d-r-u-αδ	3.7991	4.5150	4.8266	5.1181
1-d-r-u-b-αδ	3.5714	4.2854	4.5987	4.8716
1-s-r-u-αδ	8.0117	9.4973	10.1097	10.8689
2-s-r-u-a-αδ	7.8135	9.2975	9.9191	10.6865
2-s-r-u-b-αδ	7.8363	9.3284	9.9516	10.7310
1-s-r-u-a-αδ	7.8750	9.3704	9.9996	10.7377
1-d-r-ξ-δ	4.5007	5.0422	5.2230	5.4479
2-d-r-ξ-δ	5.0288	5.6629	5.8692	6.0130
3-s-w-η-γ	11.6523	13.8912	14.7599	15.6589
4-s-w-η-γ	11.8949	14.1428	15.0061	15.8790
4-s-r-b-γε	13.2242	15.5241	16.4135	17.2720
3-s-r-γε	12.7802	14.9004	15.7007	16.5722
4-s-r-a-γε	13.0892	15.1228	15.8761	16.7096
3-s-r-b-γε	13.2462	15.4862	16.3397	17.2313
2-s-r-a-βδ	13.3906	15.4372	16.1940	17.0181
1-s-r-βδ	13.7612	15.8659	16.6389	17.4392
2-s-r-b-βδ	13.4686	15.6426	16.4507	17.2773
1-s-r-βδ′	13.4492	15.5327	16.3043	17.0974
2-d-r-δ	5.3734	5.9572	6.1328	6.2537
1-d-r-b-δ	5.6984	–[a]	6.3814	6.3656
1-d-w-ξ-α	7.6963	8.6721	9.0054	9.2214
2-d-w-ξ-α	8.0724	9.1163	9.4748	9.6219
1-d-w-η-α	7.1008	8.2224	8.7100	9.1400
3-s-w-γ	11.0743		14.0703	15.0932
2-d-w-η-α	7.2181	8.3426	–[a]	9.2441
4-s-w-γ	11.0877	13.1913	14.0395	15.1277

1-d-w-u-η-α	6.3321	7.3559	7.8033	8.2233
2-d-w-u-η-α	6.4702	7.5011	7.9627	8.3819
4-s-w-u-η-τ	4.5612	5.4972	5.8753	6.1892
3-s-w-u-η-τ	4.3414	5.2095	5.5791	5.9749
1-s-w-u-η-α	2.8830	3.7653	4.2256	4.8897
1-d-r-u-b-δ	6.9740	7.9851	8.3497	8.6668
2-d-r-u-δ	7.1323	8.1318	8.4987	8.8412
4-d-w-η-τ	11.4429	13.7044	14.6625	15.7133
4-d-w-ξ-τ	11.5386	13.7902	14.7330	15.7379
2-s-w-η-β	7.9869	9.5768	10.2296	11.1083
4-d-w-u-η-τ	9.0342	10.7770	11.5170	12.3126
1-s-w-η-β	7.4687	8.8058	9.3554	10.0576
1-s-r-u-δ	12.0566	14.0644	14.8441	15.7205
2-s-r-u-b-δ	11.8833	13.9141	14.7083	15.6127
2-s-w-u-η-α	2.9836	4.0378	4.5938	5.4301
3-d-r-ξ-ε	10.3311	12.5755	13.5281	14.6449
3-d-w-η-τ	10.7005	12.9246	13.8825	14.8863
3-s-w-u-a-τ	3.7141	4.5234	4.8827	5.2872
1-s-w-u-a-α	4.2539	5.1057	5.4236	5.7855
4-d-r-ξ-ε	10.1112	12.3415	13.3287	14.4160
3-d-w-u-η-τ	7.9406	9.5733	10.2905	11.0255
3-d-w-ξ-τ	10.5139	12.7011	13.6725	14.7341
2-s-w-a-β	8.4310	9.9560	10.5662	11.3634
1-s-w-β	7.9090	9.2755	9.8225	10.3900
2-s-w-u-a-α	3.9502	4.9478	5.4234	6.0762
4-w-ξ-yτ	9.0651	11.0370	11.9234	12.9657
3-w-ξ-γτ	10.4943	12.6192	13.5373	14.6148
3-d-r-b-ε	10.2885	12.5657	13.6108	14.6599
4-d-r-ε	9.4507	11.6280	12.6583	13.8821
1-d-w-η	8.3494	10.0764	10.8767	11.7766
1-d-w-ξ	8.4906	10.1779	10.9156	11.7212
1-s-w-u-η	5.7635	7.0600	7.6975	8.5313
3-d-r-u-b-ε	7.0833	8.8455	9.6409	10.5494
1-d-w-η′	9.0338	10.8152	11.6397	12.4532
3-d-w-ξ	10.3359	12.6239	13.6176	14.7955
4-d-r-u-ε	7.3951	9.0542	9.8542	10.8419
3-d-w-η	10.6377	12.8468	13.7721	14.7652
2-r-ξ-βδ	6.8674	7.7053	7.9749	8.2760
1-r-ξ-βδ	9.7534	11.0643	11.5344	11.9505

b) Results in vacuo from DFT/B3LYP/6-31+G(d,p) full optimization calculations. Results in solution from PCM DFT/B3LYP/6-31+G(d,p) with full re-optimization, for the conformers for which full re-optimisation in solution converged in at least one solvent.

conformer	dipole moment (debye)			
	in vacuo	in chloroform	in acetonitrile	in water
1-d-r-ξ-αδ	2.1025	2.5109	2.6714	2.7637
2-d-r-ξ-αδ	3.0603		3.7615	3.7331
4-s-w-η-γτ	8.0550	10.0346		
2-d-r-αδ	2.1350	2.6717	2.9176	3.3608
4-s-w-a-γτ	6.9159	8.8655	9.8129	11.1960
3-s-w-a-γτ	7.0008			11.0567
2-s-r-u-a-αδ	7.8135		10.0430	11.0559
2-d-r-ξ-δ	5.0288		6.4041	6.6636
3-s-w-η-γ	11.6523		15.5606	16.7822
3-s-r-γε	12.7802			17.7693
4-s-r-a-γε	13.0892	15.7551	16.7922	17.9492
2-s-r-a-βδ	13.3906	15.9979	16.9766	18.0871
1-s-r-βδ	13.7612	16.4285	17.4170	18.4311
1-s-r-βδ′	13.4492			18.1364
2-d-r-δ	5.3734		6.7915	7.0692
1-d-w-ξ-α	7.6963	9.1215	9.6829	9.8869
2-d-w-ξ-α	8.0724			10.4624
1-d-w-η-α	7.1008	8.7488		
3-s-w-γ	11.0743			16.3590
2-d-w-η-α	7.2181			10.1763
1-s-w-u-η-α	2.8830			5.2267
4-d-w-η-τ	11.4429	14.3164	15.5958	16.9559
4-d-w-ξ-τ	11.5386	14.2940	15.5344	16.9490
2-s-w-η-β	7.9869	9.9353	10.8073	
3-d-r-ξ-ε	10.3311	13.0480		15.8367
3-d-w-η-τ	10.7005	13.6210	14.9059	
1-s-w-u-a-α	4.2539			5.6884
4-d-r-ξ-ε	10.1112	12.8276	14.1051	15.0410
3-d-w-ξ-τ	10.5139		14.6291	
2-s-w-a-β	8.4310			12.0624
1-s-w-β	7.9090			9.8507
2-s-w-u-a-α	3.9502			6.5281
4-w-ξ-yτ	9.0651	11.5098	12.7061	
3-w-ξ-γτ	10.4943	13.0515	14.2559	
3-d-r-b-ε	10.2885	12.9878	14.2076	
4-d-r-ε	9.4507	12.1255	13.4786	14.7643
2-r-ξ-βδ	6.8674		8.8148	
1-r-ξ-βδ	9.7534	11.5721	12.3200	12.9420

Table S 18

HOMO-LUMO energy difference of the conformers of arzanol in the media considered.

DFT/B3LYP/6-31+G(d,p) results from full optimization in vacuo and full re-optimization in solution.

Values are reported for those conformers for which PCM re-optimisation converged in at least one of the solvents. The HF values in vacuo are reported as reference to more realistic values of the HOMO-LUMO energy gap.

conformer	HOMO-LUMO energy difference (kcal/mol)				
	in vacuo		in solution, DFT		
	HF	DFT	chlrf	actn	aq
1-d-r-ξ-αδ	256.714	101.757	100.790	100.445	99.818
2-d-r-ξ-αδ	256.614	101.594		100.276	99.692
4-s-w-η-γτ	251.437	97.929	99.128		
2-d-r-αδ	256.475	101.544		100.037	99.347
4-s-w-a-γτ	251.123	97.691	98.883	98.506	97.873
3-s-w-a-γτ	251.041	97.540			97.804
2-s-r-u-a-αδ	249.083	93.693		99.611	100.245
2-d-r-ξ-δ	247.170	92.903		99.121	99.636
3-s-w-η-γ	244.697	89.552		98.356	99.479
3-s-r-γε	232.505	78.470			95.714
4-s-r-a-γε	232.260	78.194	88.454	91.955	95.745
2-s-r-a-βδ	231.532	78.206	88.767	92.470	96.298
1-s-r-βδ	231.595	78.031	88.880	92.558	96.348
1-s-r-βδ′	231.400	77.968			96.009
2-d-r-δ	247.282	93.668		99.090	99.460
1-d-w-ξ-α	246.222	89.514	94.478	96.072	97.841
2-d-w-ξ-α	246.661	89.721			97.804
1-d-w-η-α	246.442	89.640	93.944		
3-s-w-γ	244.189	89.414			99.328
2-d-w-η-α	249.799	89.301			97.804
1-s-w-u-η-α	243.618	89.138			96.731
4-d-w-η-τ	248.286	93.129	96.906	97.998	98.607
4-d-w-ξ-τ	248.638	93.599	97.032	97.917	98.513
2-s-w-η-β	247.132	94.064	98.983	99.899	
3-d-r-ξ-ε	248.500	95.149	99.328		99.523
3-d-w-η-τ	253.714	98.023	98.958	99.109	
1-s-w-u-a-α	244.496	88.811			96.875
4-d-r-ξ-ε	252.390	99.554	100.627	99.918	99.441
3-d-w-ξ-τ	253.639	98.732		99.586	
1-s-w-β	250.276	97.320			98.808
2-s-w-u-a-α	248.293	93.147			98.902
4-w-ξ-γτ	257.122	102.315	103.457	103.890	
3-w-ξ-γτ	255.892	101.399	103.031	103.746	
3-d-r-b-ε	248.908	96.141	99.561	99.818	
4-d-r-ε	252.723	100.521	100.577	99.717	
1-d-w-η	253.150	100.351			99.159

2-r-ξ-βδ	237.801	83.264		94.578	
1-r-ξ-βδ	236.991	82.448	91.485	94.766	
